# Heterometallic Ni–Pt Chini-Type Carbonyl Clusters: An Example of Molecular Random Alloy Clusters

**DOI:** 10.1021/acs.inorgchem.1c00752

**Published:** 2021-06-04

**Authors:** Cristiana Cesari, Beatrice Berti, Marco Bortoluzzi, Cristina Femoni, Maria Carmela Iapalucci, Stefano Zacchini

**Affiliations:** † Dipartimento di Chimica Industriale “Toso Montanari”, Università di Bologna, Viale Risorgimento 4, 40136 Bologna, Italy; ‡ Dipartimento di Scienze Molecolari e Nanosistemi, Ca’ Foscari University of Venice, Via Torino 155, 30175 Mestre (Ve), Italy

## Abstract

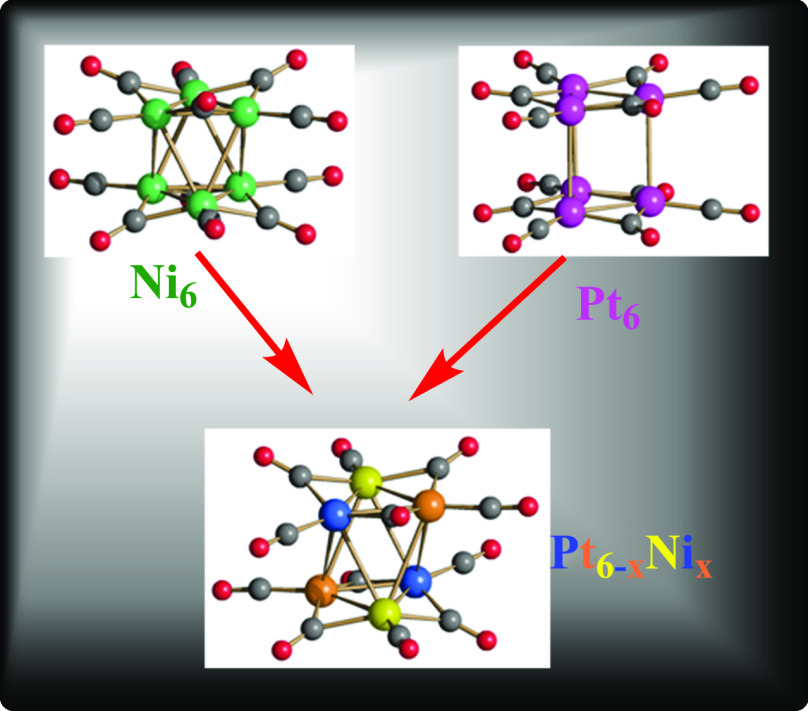

The direct reactions of homometallic [Ni_6_(CO)_12_]^2–^ and [Pt_6_(CO)_12_]^2–^ Chini carbonyl clusters result in heterometallic Ni–Pt Chini-type clusters of the general formula [Pt_6–*x*_Ni_*x*_(CO)_12_]^2–^ (*x* = 0–6). Their molecular structures have been determined by single-crystal X-ray diffraction (SC-XRD), showing a common octahedral (staggered, *D*_3*d*_) structure analogous to that of [Ni_6_(CO)_12_]^2–^, whereas [Pt_6_(CO)_12_]^2–^ displays a trigonal-prismatic (eclipsed, *D*_3*h*_) structure. This structural change after replacing one single Pt with Ni may be classified as an alloying effect, and it has been theoretically investigated by DFT methods. Spectroscopic (IR and ^195^Pt and ^13^C NMR) and ESI-MS studies indicate that mixtures of [Pt_6–*x*_Ni_*x*_(CO)_12_]^2–^ (*x* = 0–6) clusters are actually present in solution, whose compositions may be varied in an almost continuous way. Thus, they may be viewed as random alloy clusters whose overall compositions depend on the stoichiometry of the reagents.

## Abstract 2

Molecular Pt−Ni random alloy clusters adopting a Chini-type structure have been obtained upon mixing related homometallic clusters and investigated by structural, spectroscopic, and theoretical methods.

## 1 Introduction

Platinum carbonyl Chini clusters of the type [Pt_3*n*_(CO)_6*n*_]^2–^ (*n* = 1–10) have greatly contributed to cluster chemistry and inorganic chemistry in general.^1^ These are composed of Pt_3_(μ-CO)_3_(CO)_3_ units stacked along a pseudo-*C*_3_ axis, and these triangles are nearly eclipsed in the solid state, resulting in trigonal-prismatic structures. A bond analysis of Chini clusters gives major insights into the different types of M–M bonds in metal clusters.^2−5^ Thus, intratriangular Pt–Pt bonds are shorter (2.65–2.68 Å) and are almost localized, whereas intertriangular Pt–Pt bonds are longer (3.02–3.24 Å) and are highly delocalized. As a consequence, higher nuclearity Chini clusters (*n* ≥ 5) form discontinuous, semicontinuous, or continuous chains in the solid state, whose electric conductivity increases with the nuclearity (and thus the intertriangular delocalization).^6,7^ Triangle exchange has been also observed in solution by ^195^Pt NMR.^8,9^

Related nickel clusters are limited to the lower nuclearity species [Ni_6_(CO)_12_]^2–^ and [Ni_9_(CO)_18_]^2–^, probably because Ni–Ni bonds are weaker than Pt–Pt bonds; therefore, they cannot support the stacks of further triangles.^10−12^ Moreover, in the solid state, [Ni_6_(CO)_12_]^2–^ displays an octahedral (staggered, *D*_3*d*_) structure rather than the trigonal-prismatic (eclipsed, *D*_3*h*_) structure of [Pt_6_(CO)_12_]^2–^. This structural difference has been explained by Dahl, Chini and Longoni on the basis of the smaller size of Ni compared to Pt: “*The marked difference between the much longer intertriangular Pt–Pt distances of 3.04 Å in the prismatic platinum cluster vs the corresponding Ni–Ni distances of 2.77 Å in the antiprismatic nickel cluster is in accord with the premise that repulsive forces between the two halves of the dianion sufficiently increase at the smaller Ni–Ni distance to give the staggered conformation*”.^10^

It must be remarked that liquid X-ray scattering studies point out a staggered (*D*_3_) structure for both [Ni_6_(CO)_12_]^2–^ and [Pt_6_(CO)_12_]^2–^ in solution, somewhat intermediate between the solid-state structures.^13^ In addition, theoretical studies indicate that the intertriangular rotation energy barrier is rather small. [Ni_9_(CO)_18_]^2–^ displays a mixed structure, where two triangles are eclipsed as in [Pt_9_(CO)_18_]^2–^, whereas the other two are staggered, resulting in a Ni_9_ metal core composed of a trigonal prism and an octahedron fused together by a triangular face.

It was briefly reported by Longoni et al. that, upon mixing equimolar amounts of [Ni_6_(CO)_12_]^2–^ and [Pt_6_(CO)_12_]^2–^, a purported heterometallic [Pt_3_Ni_3_(CO)_6_]^2–^ cluster is formed, but it was not possible to structurally characterize it.^9,13^ In view of the renewed interest in alloy molecular clusters and nanoclusters,^14−23^ we decided to reinvestigate the chemistry of heterometallic Ni–Pt Chini-type carbonyl clusters. Herein, we report the synthesis and structural characterization by single-crystal X-ray diffraction (SC-XRD) of random alloy [Pt_6–*x*_Ni_*x*_(CO)_12_]^2–^ (*x* = 0–6) clusters, as well as spectroscopic studies (IR, ^195^Pt and ^13^C NMR, ESI-MS) in solution and theoretical investigations.

## 2 Results and Discussion

### 2.1 Synthesis and Molecular Structures of [Pt_6–*x*_Ni_*x*_(CO)_12_]^2–^ (*x* = 1.25, 2.53, 3.24, 4.15, 4.16, 4.41, 5.78, 5.90)

**Figure 1:**
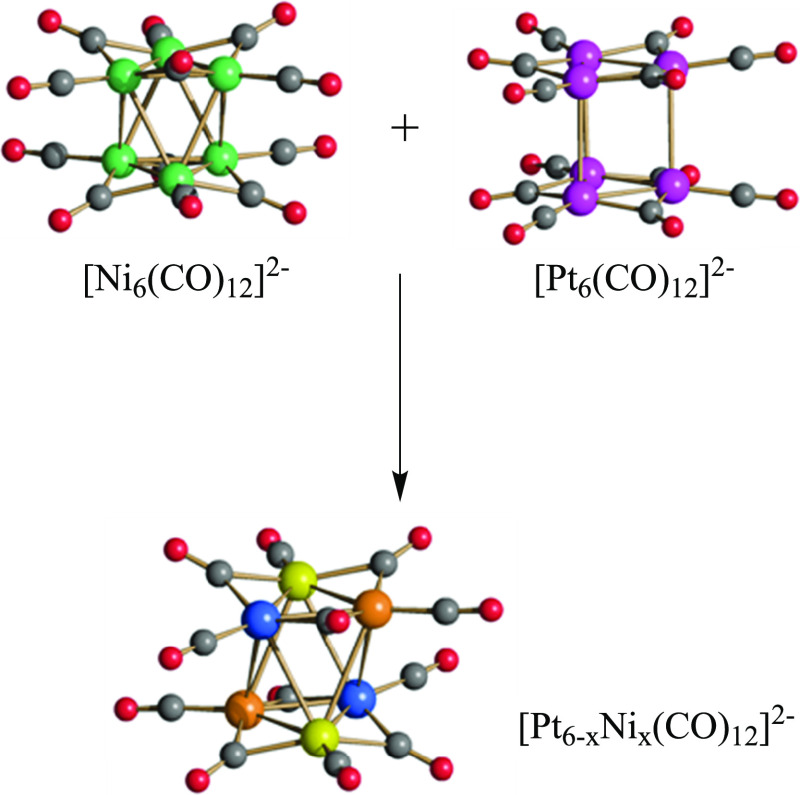
Molecular structure and synthesis of [Pt_6–*x*_Ni_*x*_(CO)_12_]^2–^ (*x* = 1.25, 2.53, 3.24, 4.15, 4.16, 4.41, 5.78, 5.90) from [Ni_6_(CO)_12_]^2–^ and [Pt_6_(CO)_12_]^2–^. Color code: green, Ni; purple, Pt; yellow, blue, and orange, disordered Ni/Pt with different occupancy factors; red, O; gray, C. The composition of [Pt_6–*x*_Ni_*x*_(CO)_12_]^2–^ is controlled by the stoichiometry of the reagents.

The direct reactions of pure samples of [Ni_6_(CO)_12_]^2–^ and [Pt_6_(CO)_12_]^2–^ in different stoichiometric amounts result in heterometallic [Pt_6–*x*_Ni_*x*_(CO)_12_]^2–^ (*x* = 0–6) clusters, whose composition can be varied in an almost continuous way just by controlling the stoichiometry of the reagents (Figure 1). Pt-rich clusters can be alternatively obtained by the reduction of [Pt_9_(CO)_18_]^2–^ with [Ni_6_(CO)_12_]^2–^. The heterometallic nature of these [Pt_6–*x*_Ni_*x*_(CO)_12_]^2–^ clusters has been fully unraveled by SC-XRD on salts of different compositions (*x* = 1.25, 2.53, 3.24, 4.15, 4.16, 4.41, 5.78, 5.90), mainly with [NBu_4_]^+^ as the counterion. The fractionary indices found by SC-XRD indicate that mixtures of clusters are actually present in the solid state. The Ni–Pt composition of the samples has been confirmed by microwave plasma-atomic emission spectrometry (MP-AES), and the nature of the different clusters composing such mixtures has been further investigated by ESI-MS (see below). The samples for MP-AES analyses have been mineralized with HNO_3_/HCl (aqua regia) and diluted with H_2_O (see the Experimental Section for details). The resulting Pt/Ni compositions are in good agreement with those determined by SC-XRD.

Experiments have been performed by mixing [Pt_6_(CO)_12_]^2–^ and [Ni_6_(CO)_12_]^2–^ in different stoichiometric ratios (1:5, 1:2, 1:1, 2:1, 5:1) in thf, and the resulting [Pt_6–*x*_Ni_*x*_(CO)_12_]^2–^ clusters have been analyzed by IR spectroscopy and MP-AES after workup (see the Experimental Section for details) of the reaction mixtures (Table 1). The Pt/Ni content found in the isolated [Pt_6–*x*_Ni_*x*_(CO)_12_]^2–^ clusters is in good agreement with that calculated on the basis of the stoichiometric ratio of the reagents. These experiments corroborate the results obtained by joint SC-XRD and MP-AES analyses (see above) and support the conclusion that the composition of the [Pt_6–*x*_Ni_*x*_(CO)_12_]^2–^ clusters can be controlled by the stoichiometry of the reaction and varied in an almost continuous way.

**Table 1:** MP-AES Study of the Reaction between [NBu_4_]_2_[Pt_6_(CO)_12_] and [NBu_4_]_2_[Ni_6_(CO)_12_]

[Pt_6_(CO)_12_]^2–^:[Ni_6_(CO)_12_]^2–^ stoichiometry	Pt:Ni calcd	Pt:Ni by MP-AES	IR (thf, 293 K) ν_CO_, cm^–1^
1:5	0.20	0.20	1983(vs), 1810(m), 1787(m)
1:2	0.50	0.56	2003(s), 1984(vs), 1809(m)
1:1	1.00	1.24	2004(vs), 1984(vs), 1802(m)
2:1	2.00	2.32	2004(vs), 1985(sh), 1802(m)
5:1	5.00	5.44	2005(vs), 1802 (m)

All of the structurally characterized [Pt_6–*x*_Ni_*x*_(CO)_12_]^2–^ clusters display an octahedral (staggered, *D*_3*d*_) structure (Figure 1 and Table 2), as was previously found in [Ni_6_(CO)_12_]^2–^. Thus, it is enough to replace one Pt with one Ni atom in order to invert the solid-state structure from trigonal prismatic to octahedral. Indeed, [NBu_4_]_2_[Pt_6_(CO)_12_] displays in the solid state a trigonal-prismatic structure (as previously found with other cations),^1^ ruling out the possibility that the cation may influence the solid-state structures of such clusters. Thus, the structural change observed on passing from [Pt_6_(CO)_12_]^2–^ to [Pt_5_Ni(CO)_12_]^2–^ may be classified as an alloying effect. Moreover, SC-XRD analyses indicate that the six metal positions of [Pt_6–*x*_Ni_*x*_(CO)_12_]^2–^ (*x* = 1.25, 2.53, 3.24, 4.15, 4.16, 4.41, 5.78, 5.90) can be randomly occupied by Ni and Pt, the overall composition of each sample depending solely on the stoichiometric ratio of the reagents adopted for the synthesis. The intratriangular M–M contacts are considerably shorter than the intertriangular M–M contacts (Table 2), as was previously found in the homometallic species [Pt_6_(CO)_12_]^2–^ and [Ni_6_(CO)_12_]^2–^. Moreover, both intra- and intertriangular M–M distances are significantly shortened by increasing the Ni contents of [Pt_6–*x*_Ni_*x*_(CO)_12_]^2–^ (*x* = 1.25, 2.53, 3.24, 4.15, 4.16, 4.41, 5.78, 5.90), as expected on the basis of the smaller covalent radius of Ni (1.24 Å) in comparison to Pt (1.36 Å).^24^

**Table 2:** M–M Distances (Å) of [Pt_6–*x*_Ni_*x*_(CO)_12_]^2–^ (*x* = 1.25, 2.53, 3.24, 4.15, 4.16, 4.41, 5.78, 5.90) Compared to Those of [Pt_6_(CO)_12_]^2–^ (*x* = 0) and [Ni_6_(CO)_12_]^2–^ (*x* = 6)^a^

*x*	M–M_intratriangle_	M–M_intertriangle_
0.00^b^	2.6519(5)–2.6572(4)	2.9947(4)–3.0150(6)
	av 2.6543(7)	av 3.0015(8)^b^
1.25	2.6456(8) −2.6468(10)	3.0757(9)–3.2064(8)
	av 2.6460(16)	av 3.1477(15)
2.53	2.6054(3)–2.6150(3)	3.0287(3)–3.2129(4)
	av 2.6086(5)	av 3.1226(5)
3.24	2.5813(6)–2.5842(6)	2.9847(6)–3.0871(6)
	av 2.5827(10)	av 3.0379(10)
4.15	2.5191(3)-2.5470(3)	2.9381(3)-3.0412(3)
	Average 2.5364(5)	Average 2.9820(5)
4.16	2.5217(4)–2.5491(4)	2.9423(3)–3.0472(4)
	av 2.5387(7)	av 2.9871(7)
4.41	2.5000(13)–2.5285(16)	2.8147(14)–3.0099(14)
	Average 2.518(2)	Average 2.955(2)
5.78	2.3873(4)-2.4179(5)	2.7767(4)-2.8425(4)
	av 2.4055(7)	av 2.7988(7)
5.90	2.3854(3)–2.4100(3)	2.7663(3)–2.8328(3)
	av 2.3988(5)	av 2.7899(5)
6.00^c^	2.375(2)–2.386(2)	2.740(2)–2.847(3)
	av 2.379(3)	av 2.779(3)

a All data are for [NBu_4_]^+^ salts at 100 K except for [Ni_6_(CO)_12_]^2–^ ([AsPh_4_]^+^, 153 K).

b Trigonal-prismatic (eclipsed, *D*_3*h*_) structure. All of the other entries adopt the octahedral (staggered, *D*_3*d*_) structure. Because of this, the M–M_intertriangle_ distances of [Pt_6_(CO)_12_]^2–^ (*x* = 0) are shorter than expected in comparison to the general trend observed for octahedral clusters with increasing Pt content.

c From ref (25).

### 2.2 IR and ESI-MS Studies

**Figure 2:**
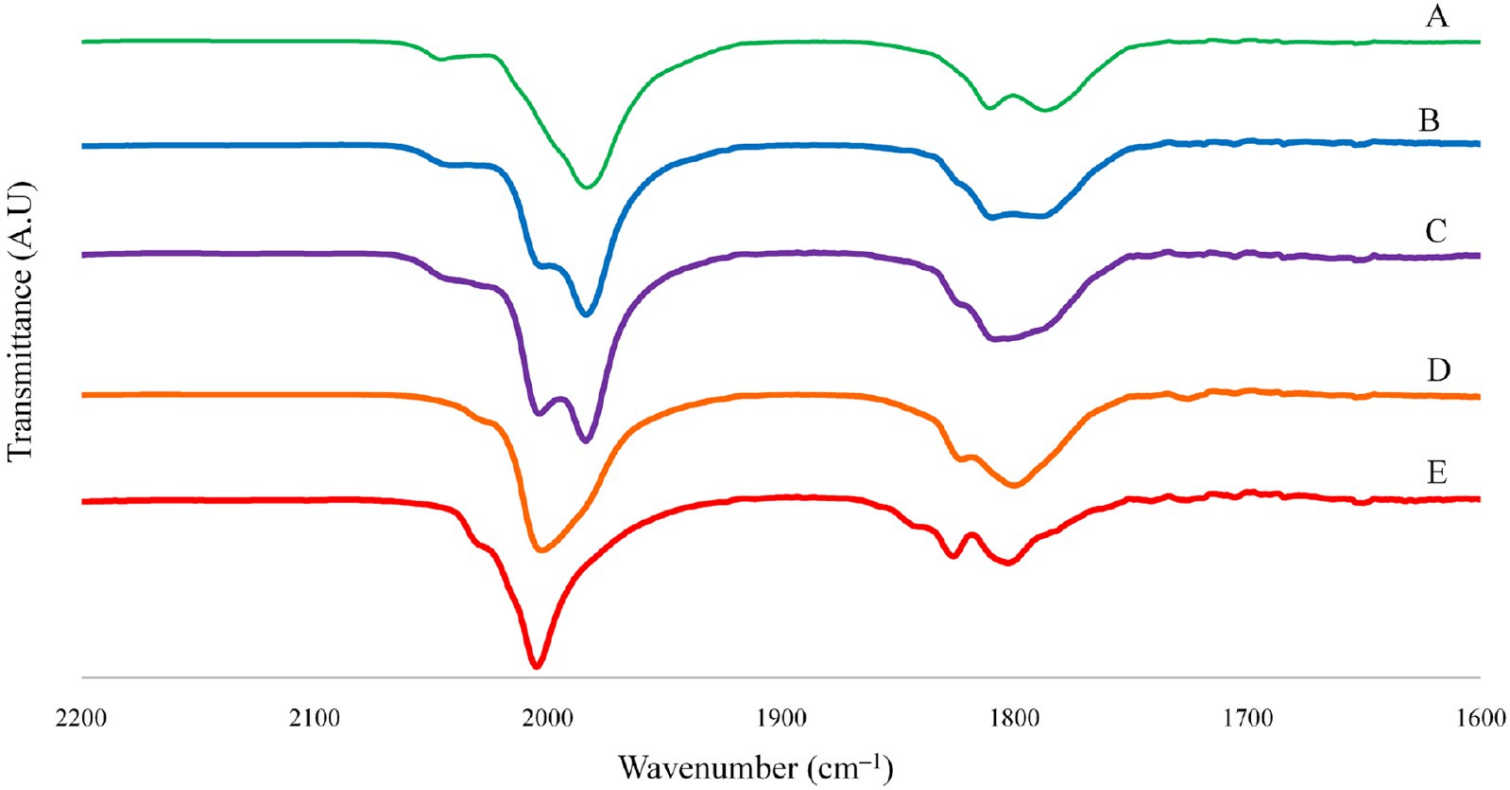
IR spectra (ν_CO_ region) recorded in thf of (A) [NBu_4_]_2_[Ni_6_(CO)_12_], (B) [NBu_4_]_2_[Pt_6_(CO)_12_] + [NBu_4_]_2_[Ni_6_(CO)_12_] (1:2 molar ratio), (C) [NBu_4_]_2_[Pt_6_(CO)_12_] + [NBu_4_]_2_[Ni_6_(CO)_12_] (1:1 molar ratio), (D) [NBu_4_]_2_[Pt_6_(CO)_12_] + [NBu_4_]_2_[Ni_6_(CO)_12_] (2:1 molar ratio), and (E) [NBu_4_]_2_[Pt_6_(CO)_12_].

The IR spectra of [Pt_6–*x*_Ni_*x*_(CO)_12_]^2–^ (*x* = 0–6) are rather broad and somewhat intermediate between those of [Ni_6_(CO)_12_]^2–^ and [Pt_6_(CO)_12_]^2–^ (Figure 2 and Figures S1–S13 in the Supporting Information). They show two main ν_CO_ bands, one in the terminal region (2003–1982 cm^–1^) and one corresponding to edge-bridging carbonyls (1809–1784 cm^–1^). The frequencies are moved to lower wavenumbers by increasing the Ni content of the [Pt_6–*x*_Ni_*x*_(CO)_12_]^2–^ (*x* = 0–6) clusters, in accordance with the lower electronegativity of Ni in comparison to Pt. In some cases two bands are present, in agreement with the formation of mixtures of products (see below).

In order to further investigate the nature of such mixtures of clusters, samples with different compositions have been studied in CH_3_CN solution by ESI-MS. The samples analyzed are crystals of [NBu_4_]_2_[Pt_6–*x*_Ni_*x*_(CO)_12_] (*x* = 1.25) and [NBu_4_]_2_[Pt_6–*x*_Ni_*x*_(CO)_12_] (mixture of *x* = 3.24, 4.15, 4.16), as well as the products obtained after workup of the reactions of [NBu_4_]_2_[Pt_9_(CO)_18_] with 1.2 mol equiv of [NBu_4_]_2_[Ni_6_(CO)_12_] and of [NBu_4_]_2_[Pt_6_(CO)_11_] with 1 mol equiv of [NBu_4_]_2_[Ni_6_(CO)_12_]. The Pt:Ni composition of these samples has been determined by MP-AES analyses, as described in the previous section and, in the case of crystalline samples, also by means of SC-XRD. The full spectra are reported in Figures S14–S43 in the Supporting Information (including calculated fits of the prominent peaks), and peak assignments are summarized in Tables S1–S4. In order to support the peak assignment, their experimental isotopic patterns have been compared with the theoretical patterns based on the formulas.

Under ESI-MS conditions, the [Pt_6–*x*_Ni_*x*_(CO)_12_]^2–^ (*x* = 0–6) clusters retain their dianionic nature, as was also corroborated by the systematic loss or addition of *m*/*z* 14 units from the molecular ions, which correspond to a CO ligand (28 amu), assuming *z* = 2. Indeed, it must be remarked that up to three CO ligands can be added or removed from [Pt_6–*x*_Ni_*x*_(CO)_12_]^2–^ (*x* = 0–6) in the gas phase. Interestingly, ^13^CO/^12^CO exchange of Chini clusters has been claimed to proceed through an associative mechanism which involves a purported [Pt_6_(CO)_13_]^2–^ species as an intermediate or transition state.^6^ Even if there is no evidence in solution for such species, the present findings show that at least in the gas phase these adducts may exist.

In some cases, also monoanionic adducts of the type {[Pt_6–*x*_Ni_*x*_(CO)_12_][NBu_4_]}^−^ (*x* = 0–6) have been observed during the ESI-MS analyses of [Pt_6–*x*_Ni_*x*_(CO)_12_]^2–^.

**Figure 3:**
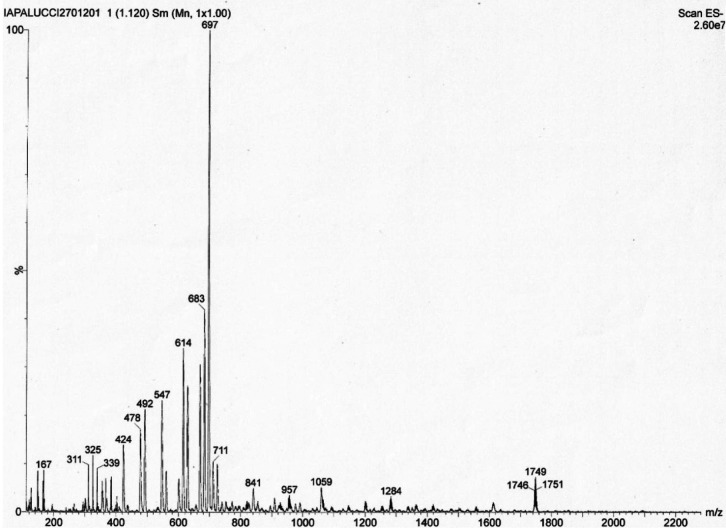
ESI-MS spectrum in CH_3_CN (ES−) of [NBu_4_]_2_[Pt_6–*x*_Ni_*x*_(CO)_12_] (*x* = 1.25).

**Figure 4:**
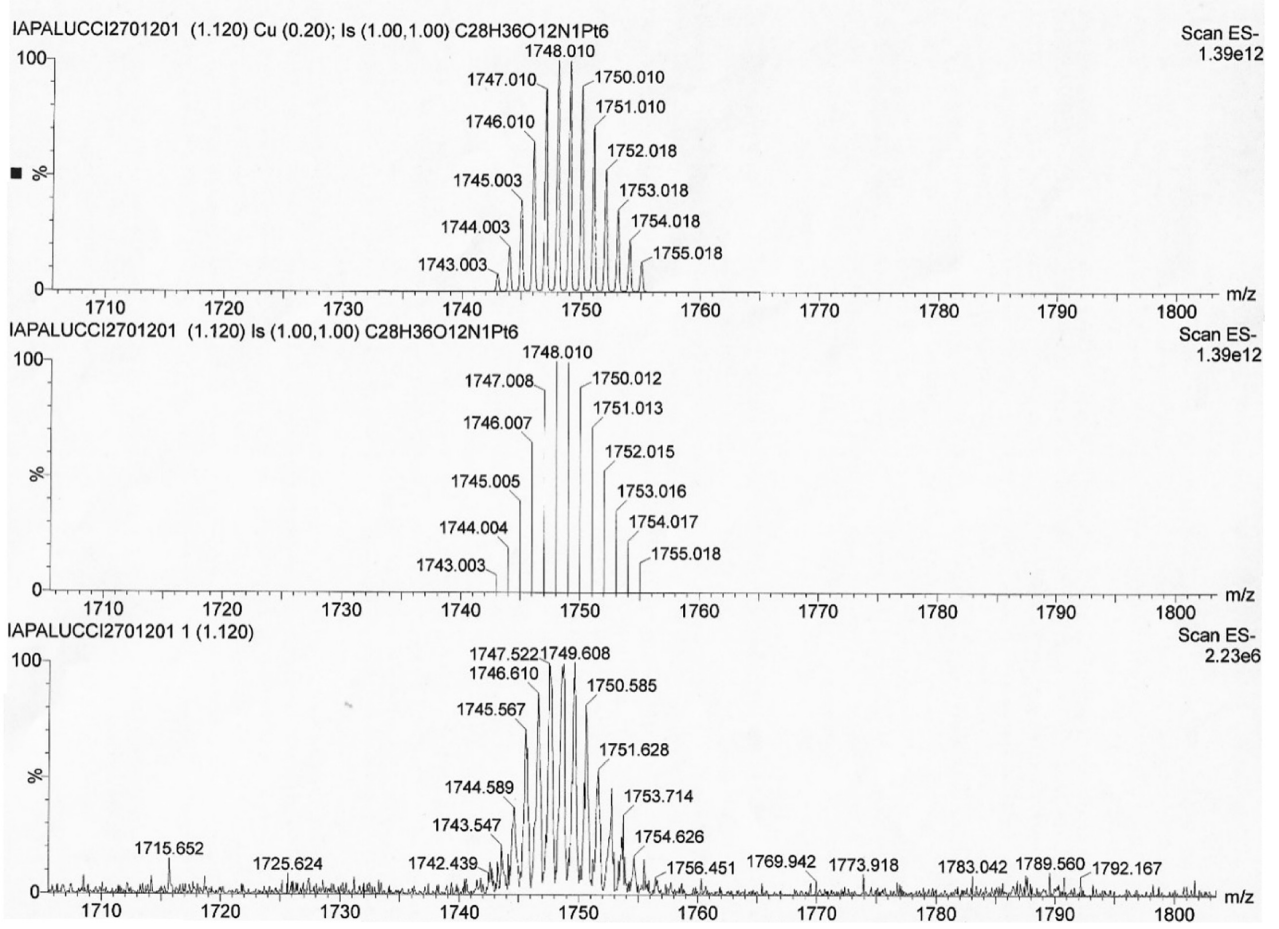
Isotopic pattern of the peak at *m*/*z* 1749 of the ESI-MS spectrum in CH_3_CN (ES−) of [NBu_4_]_2_[Pt_6–*x*_Ni_*x*_(CO)_12_] (*x* = 1.25): (top two traces) calculated isotopic patterns for {[Pt_6_(CO)_12_][NBu_4_]}^−^; (bottom trace) experimental isotopic pattern.

For instance, in the case of [NBu_4_]_2_[Pt_6–*x*_Ni_*x*_(CO)_12_] (*x* = 1.25) crystals dissolved in CH_3_CN, peaks attributable to all of the species (relative intensities in parentheses) [Pt_6_(CO)_12_]^2–^ (10), [Pt_5_Ni(CO)_12_]^2–^ (199), [Pt_4_Ni_2_(CO)_12_]^2–^ (65), [Pt_3_Ni_3_(CO)_12_]^2–^ (28), [Pt_2_Ni_4_(CO)_12_]^2–^ (35), and [PtNi_5_(CO)_12_]^2–^ (15) have been observed (Figures 3 and 4 and Table 3; further details are given in Figures S14–S21 in the Supporting Information).

**Table 3:**
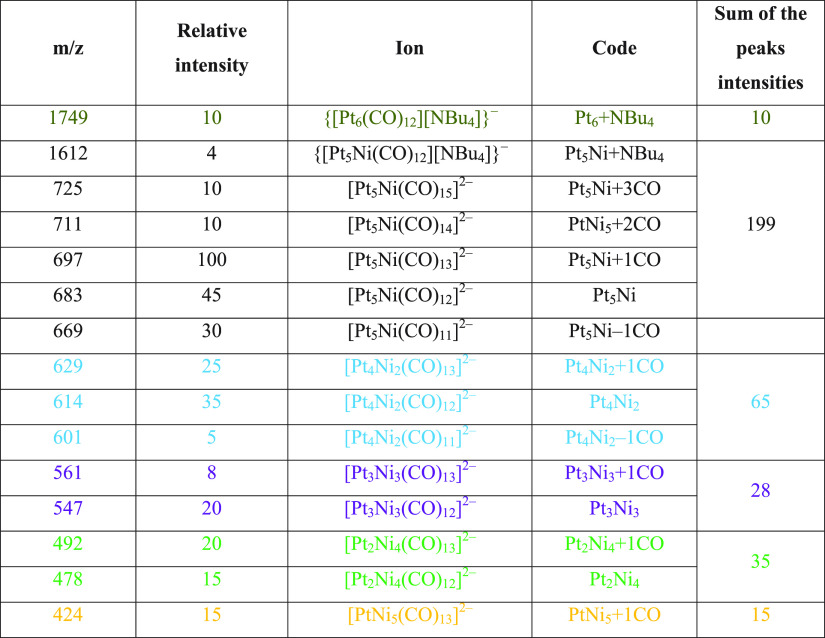
Peak Assignment of the ESI-MS Spectrum (ES−) of [NBu_4_]_2_[Pt_6–*x*_Ni_*x*_(CO)_12_] (*x* = 1.25)

**Figure 5:**
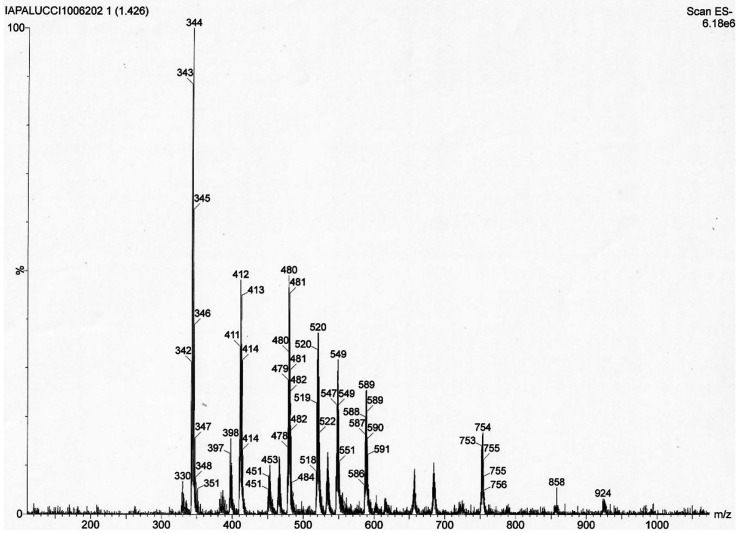
ESI-MS spectrum in CH_3_CN (ES−) of [NBu_4_]_2_[Pt_6_(CO)_11_] + [NBu_4_]_2_[Ni_6_(CO)_12_] (1:1 molar ratio) after workup.

Conversely, in the case of a sample obtained after mixing equimolar amounts of [Pt_6_(CO)_12_]^2–^ and [Ni_6_(CO)_12_]^2–^ (Figure 5 and Table 4; further details are given in Figures S37–S43 in the Supporting Information), the following clusters have been detected by ESI-MS (relative intensities in parentheses): [Pt_6_(CO)_12_]^2–^ (18), [Pt_5_Ni(CO)_12_]^2–^ (20), [Pt_4_Ni_2_(CO)_12_]^2–^ (33), [Pt_3_Ni_3_(CO)_12_]^2–^ (85), [Pt_2_Ni_4_(CO)_12_]^2–^ (72), [PtNi_5_(CO)_12_]^2–^ (70), and [Ni_6_(CO)_12_]^2–^ (108). Further examples may be found in the Supporting Information. Overall, it seems that an almost continuous distribution of [Pt_6–*x*_Ni_*x*_(CO)_12_]^2–^ (*x* = 0–6) clusters, whose composition depends on stoichiometry, can be obtained. Thus, all six positions of [Pt_6–*x*_Ni_*x*_(CO)_12_]^2–^ (*x* = 0–6) can be occupied by Ni or Pt, giving a random alloy molecular cluster, whose average composition is controlled by the stoichiometry of the reaction. Moreover, each [Pt_6–*x*_Ni_*x*_(CO)_12_]^2–^ sample is actually a complex mixture of heterometallic Chini clusters, which often comprise all of the species from *x* = 0 to *x* = 6. The predominant clusters in each sample mainly depend on the Pt:Ni ratio that, in turn, is controlled by the [Pt_6_(CO)_12_]^2–^:[Ni_6_(CO)_12_]^2–^ ratio employed for the synthesis.

**Table 4:**
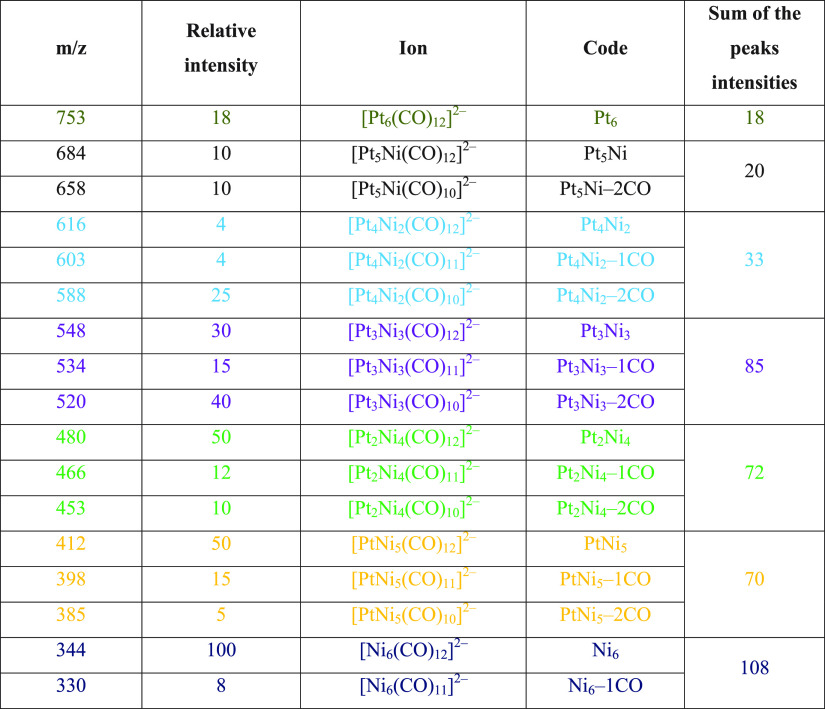
Peak Assignment of the ESI-MS Spectrum (ES−) of [NBu_4_]_2_[Pt_6_(CO)_11_] + [NBu_4_]_2_[Ni_6_(CO)_12_] (1:1 Molar Ratio) after Workup

### 2.3 NMR Studies

**Figure 6:**
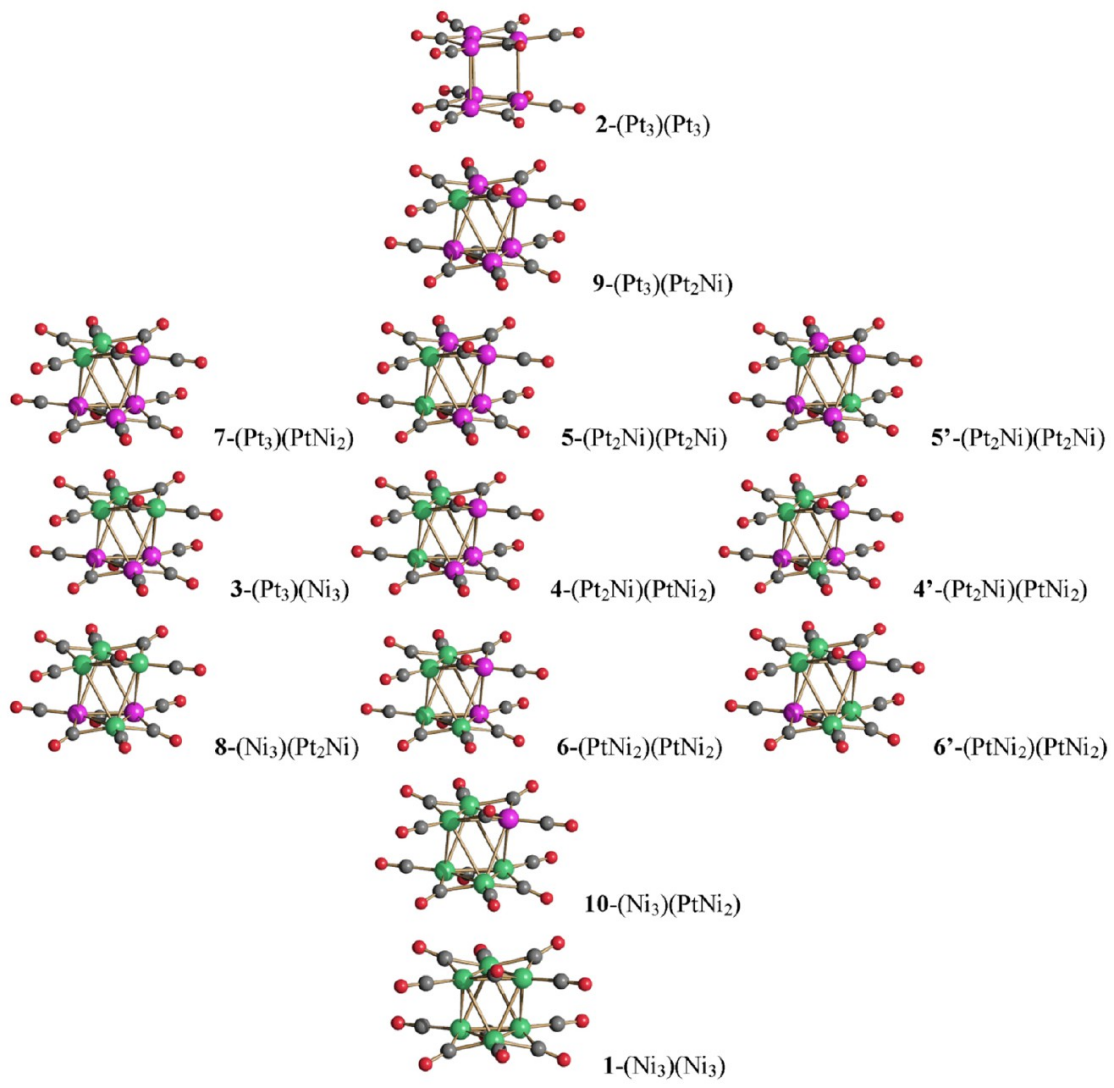
Possible isomers of [Pt_6–*x*_Ni_*x*_(CO)_12_]^2–^ (*x* = 0–6). Isomers **1**–**10** are interconverted by a combination of intermolecular triangle exchange reactions and intramolecular CO exchange. Isomers **4**/**4′**, **5**/**5′** ,and **6**/**6′** are interconverted by intramolecular triangle rotation.

**Scheme 1:**
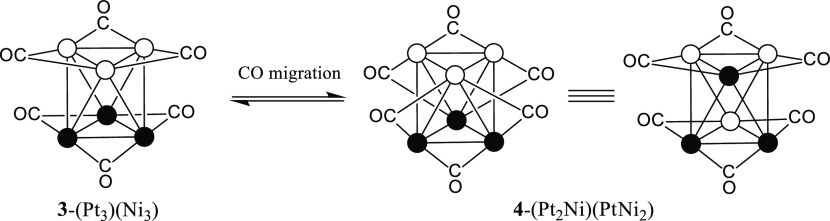
Isomerization by CO Migration of [Pt_3_Ni_3_(CO)_12_]^2–^ Only μ-CO groups are represented. Color code: white, Ni; black, Pt. Further details are given in Figures S53 and S54 in the Supporting Information.

**Scheme 2:**
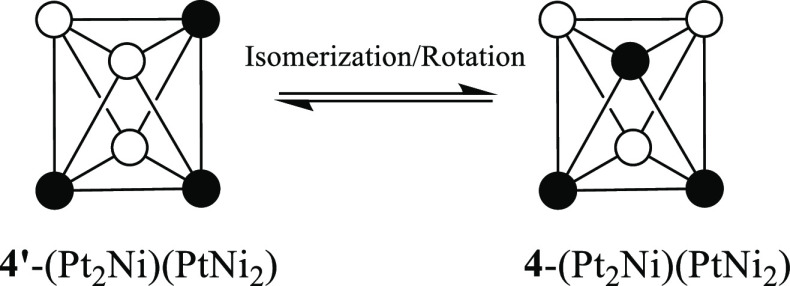
Isomerization by Triangle Rotation of [Pt_3_Ni_3_(CO)_12_]^2–^ CO groups are omitted. Color code; white, Ni; black, Pt. Further details are given in Figure S55 in the Supporting Information.

A single isomer is expected in the case of [Pt_5_Ni(CO)_12_]^2–^ and [PtNi_5_(CO)_12_]^2–^, whereas three isomers can be depicted for [Pt_4_Ni_2_(CO)_12_]^2–^, [Pt_3_Ni_3_(CO)_12_]^2–^, and [Pt_2_Ni_4_(CO)_12_]^2–^ (see Figure 6, where the isomers are labeled **1**–**10**). For each of the last species, the three isomers can be easily interconverted by a combination of intramolecular triangle rotation and CO migration processes. These are summarized in Schemes 1 and 2 for the three isomers of [Pt_3_Ni_3_(CO)_12_]^2–^ (**3**-(Pt_3_)(Ni_3_), **4**-(Pt_2_Ni)(PtNi_2_), and **4′**-(Pt_2_Ni)(PtNi_2_)). In particular, the rearrangement of the CO ligands around the octahedral metal core transforms **3**-(Pt_3_)(Ni_3_) into **4**-(Pt_2_Ni)(PtNi_2_), which in turn is transformed into **4′**-(Pt_2_Ni)(PtNi_2_) upon reciprocal rotation of the Pt_2_Ni and PtNi_2_ triangles. Details for the other species can be found in Figure S53–S55 in the Supporting Information.

CO migration has been previously observed for [Ni_6_(CO)_12_]^2–^ but not [Pt_6_(CO)_12_]^2–^.^9,26^ Variable-temperature (VT) ^195^Pt and ^13^C NMR experiments (see Figures S47–S50 in the Supporting Information) confirm that [Pt_6_(CO)_12_]^2–^ is static, whereas all other species present in the solutions of [Pt_6–*x*_Ni_*x*_(CO)_12_]^2–^ (*x* = 1–6) are somewhat fluxional, even if the spectra are too complicated to be easily interpreted. As reported by Longoni and Heaton,^26^ the CO exchange process of lowest activation energy involves a bridge–terminal carbonyl exchange, which results in randomization of the ligands of [Ni_6_(CO)_12_]^2–^. This mechanism can be extended to other [Pt_6–*x*_Ni_*x*_(CO)_12_]^2–^ (*x* = 1–5) clusters, whereas [Pt_6_(CO)_12_]^2–^ does not show a bridge–terminal carbonyl exchange.^9^ Therefore, it seems that this phenomenon is somewhat related to the octahedral (staggered, *D*_3*d*_) structure found in the solid state for [Pt_6–*x*_Ni_*x*_(CO)_12_]^2–^ (*x* = 1–6), whereas [Pt_6_(CO)_12_]^2–^ (*x* = 0) displays a trigonal-prismatic (eclipsed, *D*_3*h*_) structure. Conversely, intramolecular rotation of the two M_3_ triangles has been proposed on the basis of multinuclear VT NMR and liquid X-ray scattering studies for both [Ni_6_(CO)_12_]^2–^ and [Pt_6_(CO)_12_]^2–^.^8,9,13^

In addition to such intramolecular CO exchange processes, also intermolecular triangle exchanges are possible (see Figure S52 in the Supporting Information). Such intermolecular processes have been previously evidenced for Pt Chini clusters through VT ^195^Pt NMR experiments.^8,9^ In principle, the intermolecular exchange of M_3_(CO)_6_ units in solution may occur via either a dissociative or an associative mechanism. The latter should require the shuttling of M_3_(CO)_6_ units between [M_6_(CO)_12_]^2–^ anions. In contrast, the associative mechanism might proceed through the self-assembly of two or more [M_6_(CO)_12_]^2–^ anions into a supramolecular aggregate, following by its falling apart. Self-assembly of Pt Chini clusters has been assessed in the solid state by SC-XRD and has been partially supported in solution by dynamic light scattering (DLS) studies.^7^ Nonetheless, experimental data point out that the tendency to self-assemble increases with the nuclearity of [Pt_3*n*_(CO)_6*n*_]^2–^ (*n* = 1–10) clusters and self-assembly has not been observed for Ni Chini-type clusters. Thus, it is not possible at the moment to decide the actual mechanism, associative or dissociative, for the intermolecular triangle exchange between [Pt_6–*x*_Ni_*x*_(CO)_12_]^2–^ clusters.

**Scheme 3:**
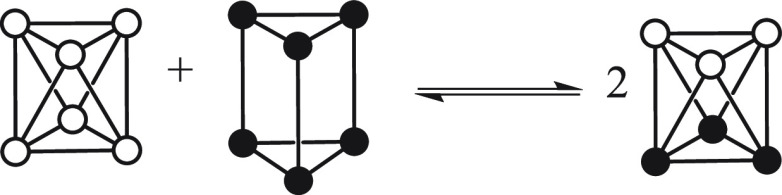
Triangle Exchange between [Ni_6_(CO)_12_]^2–^ and [Pt_6_(CO)_12_]^2–^ Color code: white, Ni; black, Pt.

**Figure 7:**
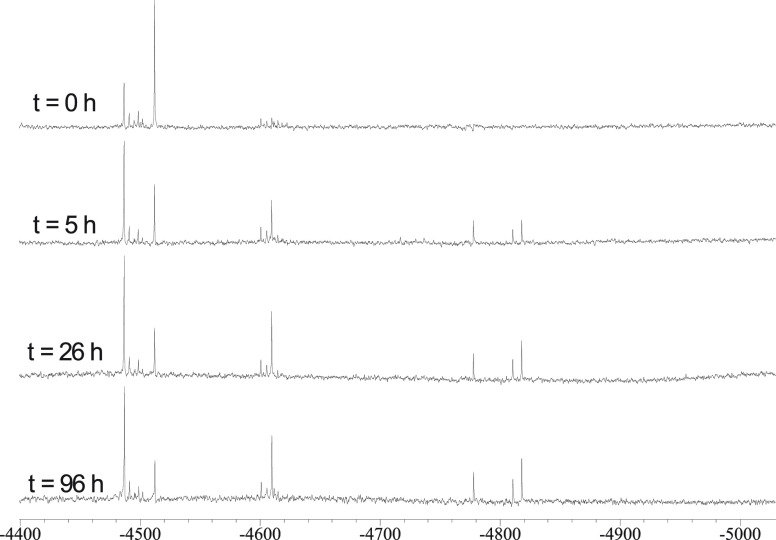
^195^Pt NMR spectra of [NBu_4_]_2_[Pt_6_(CO)_12_] + [NBu_4_]_2_[Ni_6_(CO)_12_] (1:2 molar ratio) in CD_3_COCD_3_ at 298 K recorded at different times.

Overall, the aforementioned intra- and intermolecular exchange processes are responsible for the formation of the very rich and almost continuous distribution of products observed in the reactions described in section 2.1. Indeed, we can assume that, when [Ni_6_(CO)_12_]^2–^ and [Pt_6_(CO)_12_]^2–^ are mixed, the equilibrium depicted in Scheme 3 is obtained by a statistical triangle exchange:

Indeed, two singlets at −4510 and −4485 ppm, attributable to [Pt_6_(CO)_12_]^2–^ and [Pt_3_Ni_3_(CO)_12_]^2–^ (isomer **3**-(Pt_3_)(Ni_3_), Figure 6), appear in the ^195^Pt NMR spectrum as soon as the two homometallic clusters are mixed (see Figure 7). After a few hours, the ^195^Pt NMR spectrum changes and other resonances appear, leading to very complex spectra, which depend on the stoichiometry of the reaction (see Figures S44–S46 in the Supporting Information). Even if it is not possible to assign all the resonances of these very complex spectra, they lend support to the occurrence of the intermolecular triangle exchange processes as well as intramolecular isomerization by triangle rotation and CO migration processes depicted in Schemes 1–3 and Figures S52–S55 in the Supporting Information. The overall result is the formation of complex mixtures of [Pt_6–*x*_Ni_*x*_(CO)_12_]^2–^ (*x* = 0–6) clusters differing in the composition and/or position of Ni and Pt. SC-XRD cannot help in distinguishing among the different isomers due to the presence of mixtures of clusters (as also evidenced by ESI-MS studies) and the disorder found in the solid-state structures.

**Scheme 4:**
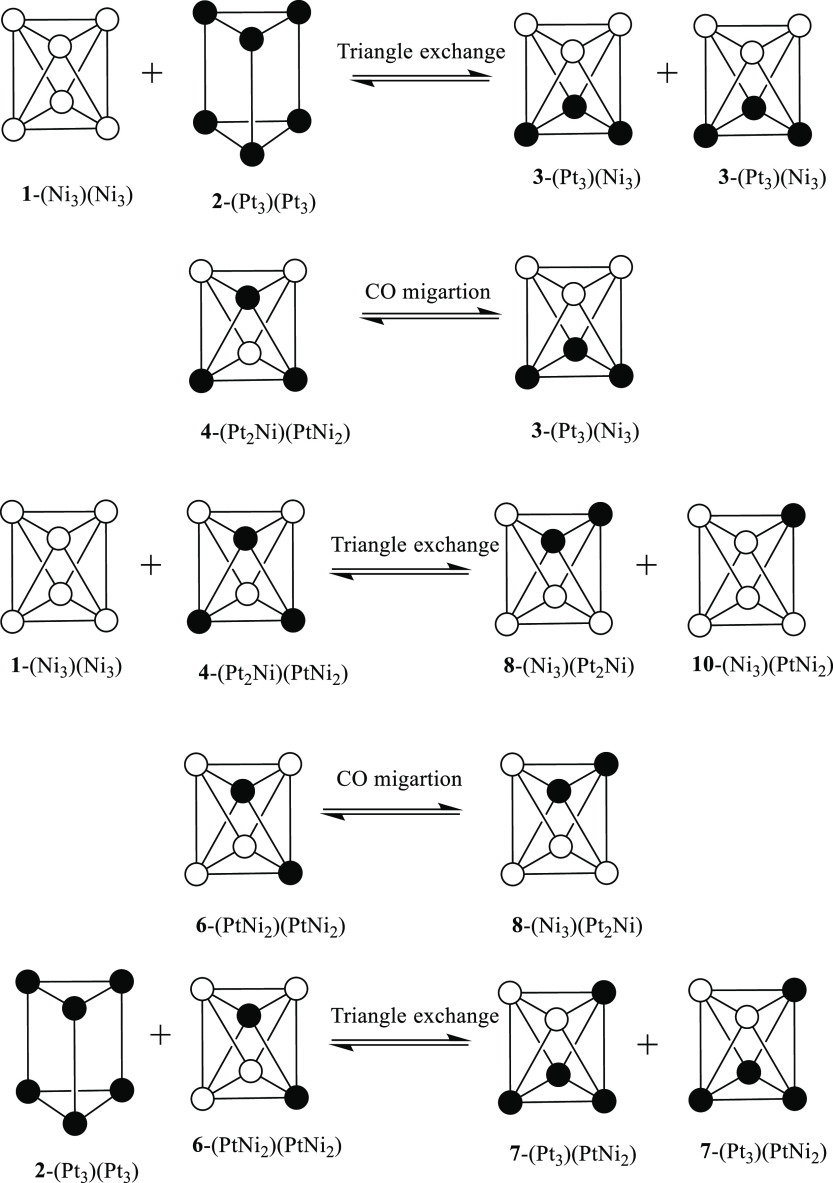
Some Representative Inter- and Intramolecular Processes for the Formation of Mixtures of [Pt_6–*x*_Ni_*x*_(CO)_12_]^2–^ (*x* = 0-6) Clusters CO groups are omitted. Color code: white, Ni; black, Pt. Further details are given in Figures S52–S55 in the Supporting Information.

Some representative inter- and intramolecular processes for the formation of mixtures of [Pt_6–*x*_Ni_*x*_(CO)_12_]^2–^ (*x* = 0–6) clusters are represented in Scheme 4. Further details can be found in Figures S52–S55 in the Supporting Information. Thus, triangle exchange between [Ni_6_(CO)_12_]^2–^ (**1**-(Ni_3_)(Ni_3_)) and [Pt_6_(CO)_12_]^2–^ (**2**-(Pt_3_)(Pt_3_)) directly results in [Pt_3_Ni_3_(CO)_12_]^2–^ (isomer **3**-(Pt_3_)(Ni_3_)), which in turn may be converted into [Pt_3_Ni_3_(CO)_12_]^2–^ (isomer **4**-(Pt_2_Ni)(PtNi_2_)) by CO migration. Subsequent exchange of a triangle between **4**-(Pt_2_Ni)(PtNi_2_) and the starting **1**-(Ni_3_)(Ni_3_) affords a mixture of [Pt_2_Ni_4_(CO)_12_]^2–^ (isomer **8**-(Ni_3_)(Pt_2_Ni)) and [PtNi_5_(CO)_12_]^2–^ (**10**-(Ni_3_)(PtNi_2_)). Migration of the CO ligands of **8**-(Ni_3_)(Pt_2_Ni) results in the isomer **6**-(PtNi_2_)(PtNi_2_), which may exchange a triangle with **2**-(Pt_3_)(Pt_3_), affording [Pt_4_Ni_2_(CO)_12_]^2–^ (isomer **7**-(Pt_3_)(PtNi_2_)). A complete list of such reactions may be found in Figures S52–S55 in the Supporting Information. Overall, independently of the stoichiometric ratio of [Ni_6_(CO)_12_]^2–^ and [Pt_6_(CO)_12_]^2–^ in the reaction, all of the [Pt_6–*x*_Ni_*x*_(CO)_12_]^2–^ (*x* = 0–6) clusters may be obtained through the triangle exchange, CO migration, and triangle rotation processes described herein. This is in keeping with the results of ESI-MS analyses and also may explain the complexity of the ^195^Pt NMR spectra.

### 2.4 Computational Studies

**Figure 8:**
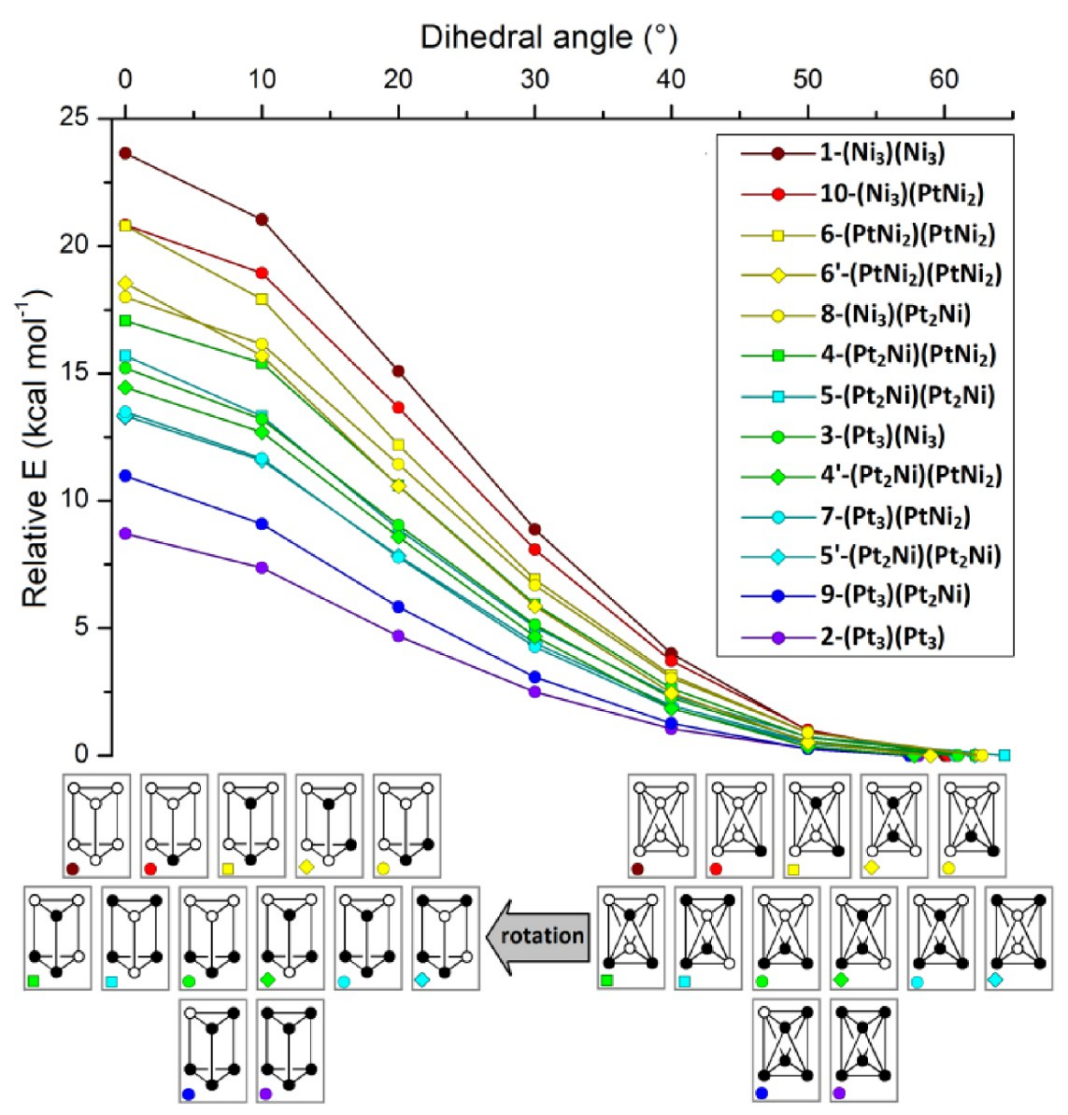
Relative energy variations of clusters **1**–**10** on changing the dihedral angle defining the relative position of the two {M_3_} triangles, from the optimized octahedral geometry to the trigonal-prismatic conformation.

The preferential formation of octahedral clusters in the presence of Ni centers was confirmed by DFT calculations. Initial attempts to optimize the trigonal-prismatic geometry of [Ni_6_(CO)_12_]^2–^ failed, as expected from the X-ray outcomes. On the other hand, the eclipsed geometry of [Pt_6_(CO)_12_]^2–^ was correctly simulated with the TPSS0 DFT functional^27^ in combination with the def2-TVZP basis set (see Figure S56 in the Supporting Information).^28^ To better investigate the role of the metallic centers on the geometry of the clusters, the ground-state octahedral structure was optimized for all of the [Pt_6–*x*_Ni_*x*_(CO)_12_]^2–^ isomers depicted in Figure 6 (see for instance the DFT-optimized structure of [Ni_6_(CO)_12_]^2–^ in Figure S56 in the Supporting Information). With the optimized staggered conformations as starting points, the dihedral angle describing the mutual position of the two {M_3_} triangles (60° for the ideal *D*_3*d*_ geometry) was varied until the trigonal-prismatic arrangement (*D*_3*h*_) was reached (0°). The energy variations associated with the process are depicted in Figure 8. The highest and lowest variations respectively correspond to [Ni_6_(CO)_12_]^2–^ and [Pt_6_(CO)_12_]^2–^, and the relative energy of the trigonal-prismatic conformations roughly grows with the Ni content. More in detail, the relative energy increases with the number of Ni–Ni intratriangular interactions. On the other hand, the *D*_3*h*_ relative energy decreases when Pt–Pt intertriangular bonds are formed, but the destabilizing effect of Ni–Ni interactions appears to be more pronounced. The relative stability of the trigonal prisms depicted in Figure 8 can be rationalized on considering the intratriangular bond strength order: Ni–Ni ≪ Ni–Pt < Pt–Pt. On the basis of the experimental outcomes, the replacement of one Pt–Pt bond with Ni–Pt is sufficient to favor the staggered disposition of the two {M_3_} triangles.

**Figure 9:**
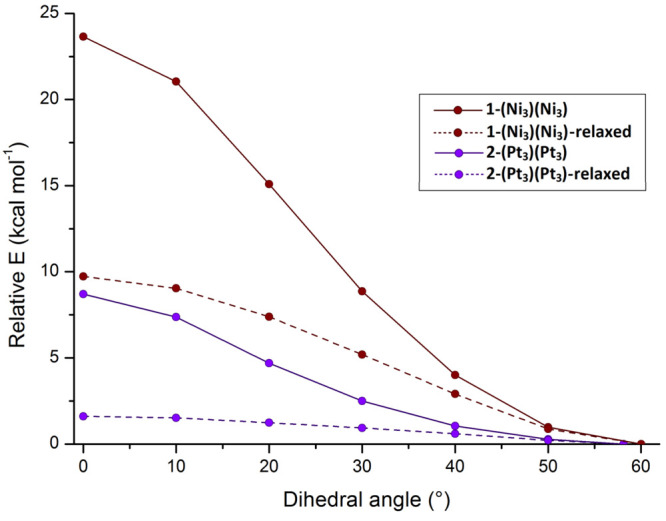
Relative energy variations of clusters **1** and **2** on changing the dihedral angle defining the relative position of the two {M_3_} triangles: (solid lines) single-point calculations; (dashed lines) geometry optimizations with constrained intertriangular dihedral angles.

The energy profiles relative to **1**-(Ni_3_)(Ni_3_) and **2**-(Pt_3_)(Pt_3_) in Figure 8 were further investigated by a geometry optimization of the structures, keeping the intertriangular dihedral angles constrained. The new profiles thus obtained (Figure 9) are characterized by lower energy variations, mainly because of the optimization of the intratriangular distances. Despite this change, the lower stability of the trigonal-prismatic arrangement for **1**-(Ni_3_)(Ni_3_) with respect to **2**-(Pt_3_)(Pt_3_) was confirmed.

**Figure 10:**
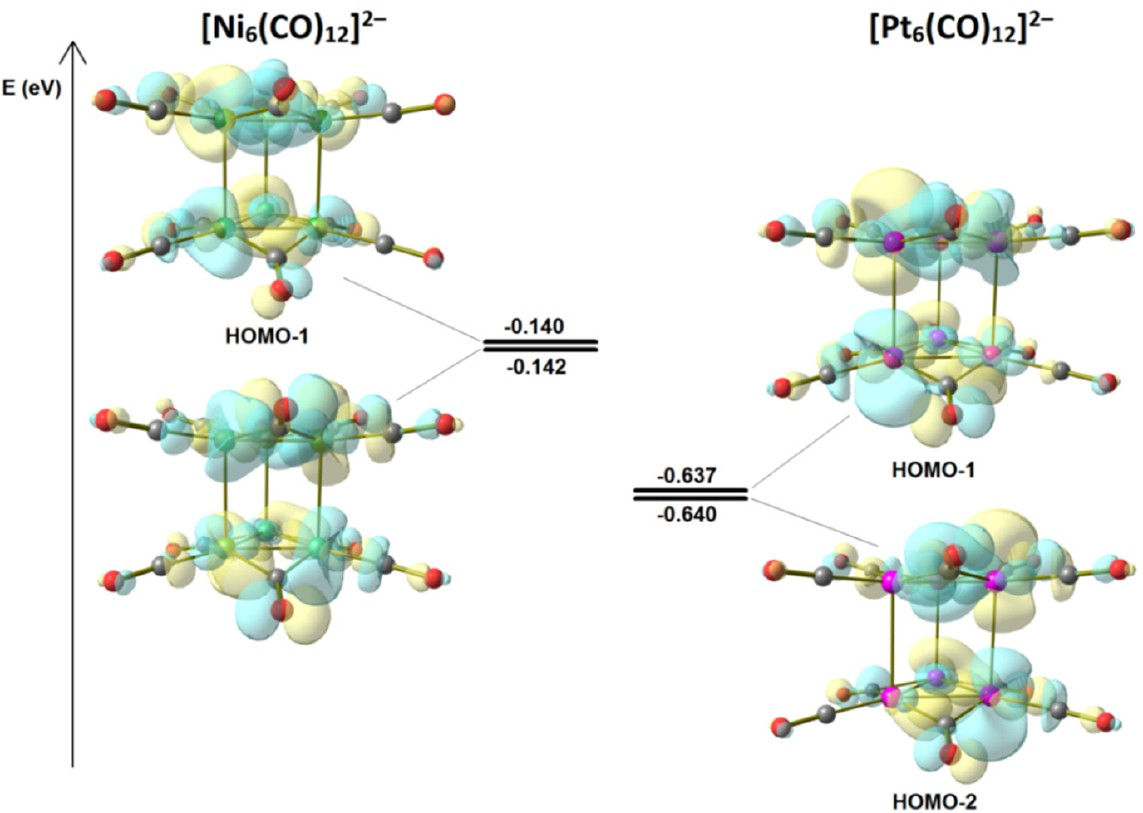
HOMO-1 and HOMO-2 orbitals and relative energies of [Ni_6_(CO)_12_]^2–^ and [Pt_6_(CO)_12_]^2–^, in constraint-optimized trigonal-prismatic configurations. Surface isovalue: 0.025 au.

The HOMO of the [Pt_6–*x*_Ni_*x*_(CO)_12_]^2–^ clusters describes in all of the cases bonding overlap between the {M_3_} triangles, in both the eclipsed and staggered dispositions. Plots of the HOMOs are provided in Figures S58 and S59 in the Supporting Information. As can be observed, there is no qualitative variation on changing the composition of the clusters. Unfortunately, the different stabilities of the conformations do not appear to be correlated to the small energy variations of the HOMO on changing the relative position of the triangles. The lower stability of the trigonal-prismatic conformation of the Ni-containing clusters can instead be associated with the reduction of the intertriangular distance caused by the replacement of Pt with Ni, as previously suggested by Dahl, Chini, and Longoni.^10^ As can be observed in Figure 10 for [Ni_6_(CO)_12_]^2–^ and [Pt_6_(CO)_12_]^2–^, the HOMO-1 and HOMO-2 molecular orbitals account for intratriangular bonds, but the same orbitals have antibonding character for the intertriangular interaction. The shortening of the intratriangular distance can cause an unfavorable overlap and therefore destabilize the eclipsed configuration. Such an assumption is corroborated almost in part by the higher energies of HOMO-1 and HOMO-2 in [Ni_6_(CO)_12_]^2–^ with respect to [Pt_6_(CO)_12_]^2–^ in a trigonal-prismatic geometry (Figure 10). Differently from the eclipsed configuration, the HOMO-1 and HOMO-2 molecular orbitals of octahedral [Ni_6_(CO)_12_]^2–^ and [Pt_6_(CO)_12_]^2–^ do not show relevant intratriangular antibonding overlaps (see Figure S60 in the Supporting Information).

An AIM analysis of the optimized octahedral [Ni_6_(CO)_12_]^2–^ and [Pt_6_(CO)_12_]^2–^ clusters and on the constraint-optimized trigonal-prismatic conformations allowed us to localize the M–M (3,–1) bond critical points (bcp.s). Electron density (ρ) values at the bcps are collected in Table 5. As can be observed, in the case of [Ni_6_(CO)_12_]^2–^ the average ρ value is lower for the trigonal-prismatic conformation with respect to the octahedral conformation, while the opposite trend was obtained for [Pt_6_(CO)_12_]^2–^. The AIM results are therefore in line with the evidence of a preferred staggered conformation of [Ni_6_(CO)_12_]^2–^ with respect to [Pt_6_(CO)_12_]^2–^.

**Table 5:** Average Electron Density Values (au) at the M–M bcp

	octahedral	trigonal prismatic
[Ni_6_(CO)_12_]^2–^	0.028	0.026
[Pt_6_(CO)_12_]^2–^	0.023	0.029

## 3 Conclusions

In conclusion, a series of [Pt_6–*x*_Ni_*x*_(CO)_12_]^2–^ (*x* = 0–6) heterometallic Ni–Pt Chini-type carbonyl clusters has been prepared by starting from the related homometallic species. These may be viewed as random alloy clusters, since all six positions of the metal cage can be occupied by Ni/Pt, generating mixtures of clusters whose overall composition depends only on the stoichiometry of the reactions. Random alloy clusters represent an alternative to site-specific doping and metal segregation that can be observed when different metals are mixed in the molecular and nanoscale domain.^14−23,29−31^

In the bulk, Ni and Pt metals both adopt a face-centered-cubic (fcc) structure (space group *Fm*3̅*m*). In the solid state, Ni and Pt are completely miscible, resulting in a continuous solid solution (random alloy), at least at higher temperatures. At lower *T* (<600 °C), some segregation is observed when the weight percent of Pt is in the range 15–100%, and the Ni_3_Pt, NiPt, and NiPt_3_ phases have been observed.^32^ In the present work, it has been shown that, at the molecular level, in the low-nuclearity [Pt_6–*x*_Ni_*x*_(CO)_12_]^2–^ (*x* = 0–6) clusters, all six metal positions can be randomly occupied by Ni and Pt, resulting in a Pt_6–*x*_Ni_*x*_ (*x* = 0–6) “continuous molecular solution”. In contrast, complete segregation has been observed in the [H_3–*n*_Ni_38_Pt_6_(CO)_44_]^*n*−^ (*n* = 3–6) molecular nanoclusters.^33,34^ Other high-nuclearity Ni–Pt molecular nanoclusters such as [Ni_35_Pt_9_(CO)_44_]^6–^ and [Ni_32_Pt_24_(CO)_56_]^6–^ display partial metal segregation as well as Ni/Pt substitutional disorder.^35,36^ Thus, it seems that the size and the composition of the molecular clusters have some effects on the Ni/Pt distribution. As more and more structures of molecular clusters and nanoclusters are determined with atomic precision, our understanding of metal segregation and substitutional and compositional disorder phenomena in alloy nanoclusters is increasing.^37,38^

As a final remark, Chini clusters were discovered by Chini and Longoni almost 50 years ago. Their molecular structures in the solid state have been disclosed for the first time, thanks to a collaboration with Larry Dahl. The fluxional behavior of such clusters in solution was disclosed by the work of Longoni and Heaton. New achievements have been obtained in the past decade with regard to the self-assembly of Chini clusters, their electrical conductivity in the solid state, and the formation of heteroleptic and heterometallic Chini-type clusters as well as their potential applications.^4−7,39−42^ Moreover, it has been demonstrated recently that Chini-type clusters can display new reactivity and electronic states upon ligand substitution.^43^ They represent a continuous challenge, which we believe it worth of pursuing.

## 4 Experimental Section

### 4.1 General Experimental Procedures

All reactions and sample manipulations were carried out using standard Schlenk techniques under nitrogen and in dried solvents. All of the reagents were commercial products (Aldrich) of the highest purity available and were used as received, except [NR_4_]_2_[Pt_3*n*_(CO)_6*n*_] (*n* = 2–4)^1^ and [NR_4_]_2_[Ni_6_CO)_12_] (R = Et, Bu),^44^ which have been prepared according to the literature. Analyses of C, H, and N were obtained with a Thermo Quest Flash EA 1112NC instrument. Analysis of Ni and Pt were performed by microwave plasma-atomic emission spectrometry on a Agilent 4210 MP-AES instrument. IR spectra were recorded on a PerkinElmer Spectrum One interferometer in CaF_2_ cells. ESI mass spectra were recorded on a Waters Micromass ZQ4000 instrument using CH_3_CN as the solvent (source temperature 150 °C; capillary voltage 2.54 kV; infusion flow 20 μL/min; cone voltage 10 V). ^195^Pt and ^13^C{^1^H} NMR measurements were performed on Varian Mercury Plus 400 MHz and Varian Inova 300 MHz spectrometers. The carbon chemical shifts were referenced to a nondeuterated aliquot of the solvent. The platinum chemical shifts were referenced to external Na_2_PtCl_6_ (1.2 M in D_2_O). Structure drawings have been created with SCHAKAL99.^45^

*Caution*! CO and Ni(CO)_4_ may be generated during manipulation of these compounds. All of the operations must be carried out under a well-ventilated fume hood.

### 4.2 Synthesis of [NBu_4_]_2_[Pt_6_(CO)_12_]

NaOH (0.680 g, 17.0 mmol) was added as a solid to a solution of [NBu_4_]_2_[Pt_12_(CO)_24_] (1.14 g, 0.326 mmol) in dmso (20 mL) under a CO atmosphere. The mixture was stirred for 0.5 h at room temperature, and then H_2_O (3.5 mL) was added dropwise. The solution was further stirred for 1 h under a CO atmosphere, and then a saturated solution of [NBu_4_]Br in H_2_O (50 mL) was added, causing the precipitation of [NBu_4_]_2_[Pt_6_(CO)_12_]. The product was recovered by filtration, washed with H_2_O (2 × 20 mL), dried under reduced pressure, and eventually extracted with acetone (20 mL). Crystals of [NBu_4_]_2_[Pt_6_(CO)_12_] suitable for SC-XRD were obtained by slow diffusion of *n*-hexane (50 mL) into the acetone solution (yield 1.05 g, 81% based on Pt).

Anal. Calcd for C_44_H_72_N_2_O_12_Pt_6_ (1991.57): C, 26.53; H, 3.65; N, 1.41. Found: C, 26.34; H, 3.79; N, 1.11. IR (CH_3_CN, 293 K): ν_CO_ 2005(vs), 1802(m) cm^–1^.

### 4.3 Synthesis of [NBu_4_]_2_[Pt_6–*x*_Ni_*x*_(CO)_12_] (*x* = 1.25)

A solution of [NBu_4_]_2_[Pt_9_(CO)_18_] (1.00 g, 0.365 mmol) in thf (30 mL) was added dropwise to a solution of [NBu_4_]_2_[Ni_6_(CO)_12_] (0.494 g, 0.420 mmol) in thf (20 mL). The mixture was stirred for 2 h at room temperature, and then the solvent was removed under reduced pressure. The residue was washed with H_2_O (2 × 15 mL) and toluene (2 × 15 mL) and extracted with thf (50 mL). Crystals of [NBu_4_]_2_[Pt_6–*x*_Ni_*x*_(CO)_12_] (*x* = 1.25) suitable for SC-XRD were obtained by slow diffusion of *n*-hexane (150 mL) into the thf solution (yield 0.77 g, 61% based on Pt, 21% based on Ni). The Pt/Ni content of the final compound was determined by MP-AES before (Pt/Ni = 3.34) and after crystallization (Pt:Ni = 3.38), showing very similar results, which are very close to those expected on the basis of the composition of the crystals determined by SC-XR:D (Pt:Ni = 3.80). These values are considerably higher than the Pt:Ni content on the basis of the stoichiometry of the reagents (Pt:Ni = 1.30). Thus, part of the Ni is eliminated during the workup of the reaction mixture.

Anal. Calcd for C_44_H_72_N_2_Ni_1.25_O_12_Pt_4.75_ (1820.41): C, 29.03; H, 3.99; N, 1.54. Found: C, 29.34; H, 4.15; N, 1.31. MP-AES: calcd Pt:Ni 3.80; found (crystals) 3.38; found (before crystallization) 3.34. IR (Nujol, 293 K): ν_CO_ 1997(vs), 1980(s), 1818(m), 1779(s) cm^–1^. IR (CH_3_CN, 293 K): ν_CO_ 2005(vs), 1797(m) cm^–1^.

### 4.4 Synthesis of [NBu_4_]_2_[Pt_6–*x*_Ni_*x*_(CO)_12_] (*x* = 3.24), [NBu_4_]_2_[Pt_6–*x*_Ni_*x*_(CO)_12_] (*x* = 4.15), and [NBu_4_]_2_[Pt_6–*x*_Ni_*x*_(CO)_12_] (*x* = 4.16)

A solution of [NBu_4_]_2_[Ni_6_(CO)_12_] (0.700 g, 0.600 mmol) in thf (30 mL) was added dropwise to a solution of [NBu_4_]_2_[Pt_6_(CO)_12_] (0.950 g, 0.478 mmol) in thf (20 mL). The mixture was stirred for 2 h at room temperature, and then the solvent was removed under reduced pressure. The residue was washed with H_2_O (2 × 15 mL) and toluene (2 × 15 mL) and extracted with CH_2_Cl_2_ (35 mL). A mixture of crystals of [NBu_4_]_2_[Pt_6–*x*_Ni_*x*_(CO)_12_] (*x* = 3.24), [NBu_4_]_2_[Pt_6–*x*_Ni_*x*_(CO)_12_] (*x* = 4.15), and [NBu_4_]_2_[Pt_6–*x*_Ni_*x*_(CO)_12_] (*x* = 4.16) suitable for SC-XRD were obtained by slow diffusion of *n*-hexane (100 mL) into the CH_2_Cl_2_ solution (yield 0.88 g, 55% based on Pt, 51% based on Ni). The Pt:Ni content of the mixture of crystals was determined by MP-AES, giving a result very close to that of [NBu_4_]_2_[Pt_6–*x*_Ni_*x*_(CO)_12_] (*x* = 3.24). Thus, this was assumed to be the major component of the mixture and used for the calculation of the yields. The Pt:Ni content of [NBu_4_]_2_[Pt_6–*x*_Ni_*x*_(CO)_12_] (*x* = 3.24) (Pt:Ni = 0.85) is also closer to that of the reagents (Pt:Ni = 0.80) in comparison to [NBu_4_]_2_[Pt_6–*x*_Ni_*x*_(CO)_12_] (*x* = 4.15) (Pt:Ni = 0.45) and [NBu_4_]_2_[Pt_6–*x*_Ni_*x*_(CO)_12_] (*x* = 4.16) (Pt:Ni = 0.44). Thus, the reaction mixture seems to retain the Pt:Ni ratio of the reagents.

Anal. Calcd for C_44_H_72_N_2_Ni_3.24_O_12_Pt_2.76_ (1550.38): C, 34.15; H, 4.69; N, 1.81. Calcd for C_44_H_72_N_2_Ni_4.15_O_12_Pt_1.85_ (1425.59): C, 37.14; H, 5.10; N, 1.97. Calcd for C_44_H_72_N_2_Ni_4.16_O_12_Pt_1.84_ (1424.23): C, 37.17; H, 5.11; N, 1.97. Found: C, 36.85, H, 4.89, N, 1.96. MP-AES: calcd Pt:Ni 0.85 (C_44_H_72_N_2_Ni_3.24_O_12_Pt_2.76_); calcd 0.45 (C_44_H_72_N_2_Ni_4.15_O_12_Pt_1.85_); calcd 0.44 (C_44_H_72_N_2_Ni_4.16_O_12_Pt_1.84_); found 0.86. IR (Nujol, 293 K): ν_CO_ 2025(vw), 1966(vs), 1811(m) 1783(s) cm^–1^. IR (CH_2_Cl_2_, 293 K): ν_CO_ 1996(vs), 1793(m) cm^–1^. IR (thf, 293 K): ν_CO_ 1992(vs), 1798(m) cm^–1^. IR (acetone, 293 K): ν_CO_ 1989(vs), 1801(m) cm^–1^. IR (CH_3_CN, 293 K): ν_CO_ 1995(vs), 1798(m) cm^–1^. IR (dmso, 293 K): ν_CO_ 1990(vs), 1797(m) cm^–1^.

### 4.5 Synthesis of [NBu_4_]_2_[Pt_6–*x*_Ni_*x*_(CO)_12_] (*x* = 4.41)

A solution of [NBu_4_]_2_[Ni_6_(CO)_12_] (1.38 g, 1.18 mmol) in thf (40 mL) was added dropwise to a solution of [NBu_4_]_2_[Pt_6_(CO)_12_] (0.950 g, 0.478 mmol) in thf (20 mL). The mixture was stirred for 2 h at room temperature, and then the solvent was removed under reduced pressure. The residue was washed with H_2_O (2 × 15 mL) and toluene (2 × 15 mL) and extracted with thf (35 mL). Crystals of [NBu_4_]_2_[Pt_6–*x*_Ni_*x*_(CO)_12_] (*x* = 4.41) suitable for SC-XRD were obtained by slow diffusion of *n*-hexane (100 mL) into the thf solution (yield 0.93 g, 37% based on Pt, 42% based on Ni).

Anal. Calcd for C_44_H_72_N_2_Ni_4.41_O_12_Pt_1.59_ (1390.14): C, 38.10; H, 5.24; N, 2.02. Found: C, 38.31; H, 5.02; N, 1.84. IR (CH_2_Cl_2_, 293 K): ν_CO_ 1989(vs), 1788(m) cm^–1^.

### 4.6 Synthesis of [NBu_4_]_2_[Pt_6–*x*_Ni_*x*_(CO)_12_] (*x* = 5.78)

A solution of [NBu_4_]_2_[Ni_6_(CO)_12_] (1.60 g, 1.37 mmol) in thf (50 mL) was added dropwise to a solution of [NBu_4_]_2_[Pt_6_(CO)_12_] (0.216 g, 0.109 mmol) in thf (15 mL). The mixture was stirred for 2 h at room temperature, and then the solvent was removed under reduced pressure. The residue was washed with H_2_O (2 × 15 mL) and toluene (2 × 15 mL) and extracted with thf (40 mL). Crystals of [NBu_4_]_2_[Pt_6–*x*_Ni_*x*_(CO)_12_] (*x* = 5.78) suitable for SC-XRD were obtained by slow diffusion of *n*-hexane (100 mL) into the thf solution (yield 1.01 g, 28% based on Pt, 59% based on Ni).

Anal. Calcd for C_44_H_72_N_2_Ni_5.78_O_12_Pt_0.22_ (1203.35): C, 44.06; H, 6.06; N, 2.34. Found: C, 44.39; H, 5.84; N, 2.44. IR (thf, 293 K): ν_CO_ 1983(vs), 1811(m), 1786(ms) cm^–1^.

### 4.7 Synthesis of [NBu_4_]_2_[Pt_6–*x*_Ni_*x*_(CO)_12_] (*x* = 5.90)

A solution of [NBu_4_]_2_[Ni_6_(CO)_12_] (1.40 g, 1.20 mmol) in thf (50 mL) was added dropwise to a solution of [NBu_4_]_2_[Pt_6_(CO)_12_] (0.0982 g, 0.0495 mmol) in thf (15 mL). The mixture was stirred for 2 h at room temperature, and then the solvent was removed under reduced pressure. The residue was washed with H_2_O (2 × 15 mL) and toluene (2 × 15 mL) and extracted with thf (40 mL). Crystals of [NBu_4_]_2_[Pt_6–*x*_Ni_*x*_(CO)_12_] (*x* = 5.90) suitable for SC-XRD were obtained by slow diffusion of *n*-hexane (100 mL) into the thf solution (yield 0.98 g, 28% based on Pt, 68% based on Ni).

Anal. Calcd for C_44_H_72_N_2_Ni_5.90_O_12_Pt_0.10_ (1186.93): C, 44.67; H, 6.14; N, 2.37. Found: C, 44.38; H, 6.39; N, 2.08. IR (thf, 293 K): ν_CO_ 1983(vs), 1811(m), 1786(ms) cm^–1^.

### 4.8 Synthesis of [NBu_4_]_4_[Pt_6–*x*_Ni_*x*_(CO)_12_][Cl_1.77_Br_0.23_] (*x* = 2.53)

A solution of [NBu_4_]_2_[Ni_6_(CO)_12_] (0.450 g, 0.385 mmol) in thf (30 mL) was added dropwise to a solution of [NBu_4_]_2_[Pt_6_(CO)_12_] (0.950 g, 0.478 mmol) in thf (20 mL). The mixture was stirred for 2 h at room temperature, and then the crude product was precipitated by addition of a saturated solution of [NBu_4_]Cl in H_2_O (60 mL). The solid was recovered after filtration, washed with H_2_O (2 × 15 mL) and toluene (2 × 15 mL), and extracted with thf (30 mL). Crystals of [NBu_4_]_4_[Pt_6–*x*_Ni_*x*_(CO)_12_][Cl_1.77_Br_0.23_] (*x* = 2.53) suitable for SC-XRD were obtained by slow diffusion of *n*-hexane (100 mL) into the thf solution (yield 1.26 g, 69% based on Pt, 62% based on Ni). The presence of some Br^–^ ions in the crystals is due to contamination of the starting [NBu_4_]_2_[Ni_6_(CO)_12_] salt with some [NBu_4_]Br.

Anal. Calcd for C_76_H_144_Br_0.23_Cl_1.77_N_4_Ni_2.53_O_12_Pt_3.48_ (2213.03): C, 41.26; H, 6.57; N, 2.53. Found: C, 41.08; H, 6.74; N, 2.69. IR (thf, 293 K): ν_CO_ 1995(vs), 1797(m) cm^–1^. IR (CH_3_CN, 293 K): ν_CO_ 1997(vs), 1796(m) cm^–1^.

### 4.9 Synthesis of [NBu_4_]_2_[Pt_9-x_Ni_*x*_(CO)_18_] (*x* = 1.65) and Crystals of [NBu_4_]_2_[Pt_9_(CO)_18_]·thf

A solution of [NBu_4_]_2_[Ni_6_(CO)_12_] (0.428 g, 0.365 mmol) in thf (20 mL) was added dropwise to a solution of [NBu_4_]_2_[Pt_12_(CO)_24_] (1.28 g, 0.365 mmol) in thf (30 mL). The mixture was stirred for 2 hat room temperature, and then the solvent was removed under reduced pressure. The residue was washed with H_2_O (2 × 15 mL) and toluene (2 × 15 mL) and extracted with thf (30 mL). After filtration, the thf solution was evaporated to dryness resulting, in a microcrystalline powder of a compound formulated as [NBu_4_]_2_[Pt_9-x_Ni_*x*_(CO)_18_] (*x* = 1.65) on the basis of IR and MP-AES analyses (yield 0.82 g, 64% based on Pt, 29% based on Ni).

Anal. Calcd for C_54_H_80_N_2_O_19_Ni_1.65_Pt_7.35_ (2517.06): C, 23.84; H, 2.88; N, 1.11. Found: C, 23.29; H, 2.51; N, 0.81. MP-AES: calcd Pt:Ni 4.45; found 4.47. IR (thf, 293 K): ν_CO_: 2030(vs), 1844(ms) cm^–1^.

All attempts to crystallize [NBu_4_]_2_[Pt_9-x_Ni_*x*_(CO)_18_] (*x* = 1.65) by slow diffusion of *n*-hexane (80 mL) into the thf solution failed. Indeed, only a few crystals of [NBu_4_]_2_[Pt_9_(CO)_18_]**·**thf suitable for SC-XRD were obtained.

### 4.10 MP-AES Study of the Reaction between [NBu_4_]_2_[Pt_6_(CO)_12_] and [NBu_4_]_2_[Ni_6_(CO)_12_]

A solution containing a variable amount of [NBu_4_]_2_[Ni_6_(CO)_12_] (*m*_Ni_6_(CO)_12__, see list below) in thf (20 mL) was added dropwise to a solution of [NBu_4_]_2_[Pt_6_(CO)_12_] (0.410 g, 0.206 mmol) in thf (20 mL). The mixture was stirred for 2 h at room temperature, and then the solvent was removed under reduced pressure. The residue was washed with H_2_O (2 × 15 mL) and toluene (2 × 15 mL) and extracted with thf (40 mL). The resulting solution was analyzed by means of MP-AES in order to determine the Pt:Ni content and compare it with that of the starting reagents.

*m*_Ni_6_(CO)_12__ = 1.21 g (1.03 mol; Pt_6_:Ni_6_ = 1:5). MP-AES: calcd Pt:Ni 0.20; found 0.20. IR (thf, 293 K): ν_CO_ 1983(vs), 1810(m), 1787(m) cm^–1^.

*m*_Ni_6_(CO)_12__ = 0.483 g (0.412 mol; Pt_6_:Ni_6_ = 1:2). MP-AES: calcd Pt;Ni 0.50; found 0.56. IR (thf, 293 K): ν_CO_ 2003(s), 1984(vs), 1809(m) cm^–1^.

*m*_Ni_6_(CO)_12__ = 0.241 g (0.206 mol; Pt_6_:Ni_6_ = 1:1). MP-AES: calcd Pt:Ni 1.00; found 1.24. IR (thf, 293 K): ν_CO_ 2004(vs), 1984(vs), 1802(m) cm^–1^.

*m*_Ni_6_(CO)_12__ = 0.121 g (0.103 mol; Pt_6_:Ni_6_ = 2:1). MP-AES: calcd Pt:Ni 2.00; found 2.32. IR (thf, 293 K): ν_CO_ 2004(vs), 1985(sh), 1802(m) cm^–1^.

*m*_Ni_6_(CO)_12__ = 0.0483 g (0.0412 mol; Pt_6_:Ni_6_ = 5:1). MP-AES: calcd Pt:Ni 5.00; found 5.44. IR (thf, 293 K): ν_CO_ 2005(vs), 1802 (m) cm^–1^.

### 4.11 MP-AES Analyses

For a typical analysis, 4–5 mg of the sample, accurately weighed with an analytical balance (±0.0001 g), was placed in a 100 mL volumetric flask and completely dissolved with a few drops of aqua regia (HCl:HNO_3_ 3:1 v:v). Then, distilled H_2_O was added up to a total volume of 100 mL. The resulting sample was directly used for MP-AES analyses.

### 4.12 X-ray Crystallographic Study

Crystal data and collection details for [NBu_4_]_2_[Pt_6–*x*_Ni_*x*_(CO)_12_] (*x* = 1.25), [NBu_4_]_2_[Pt_6–*x*_Ni_*x*_(CO)_12_] (*x* = 3.24), [NBu_4_]_2_[Pt_6–*x*_Ni_*x*_(CO)_12_] (*x* = 4.15), [NBu_4_]_2_[Pt_6–*x*_Ni_*x*_(CO)_12_] (*x* = 4.16), [NBu_4_]_2_[Pt_6–*x*_Ni_*x*_(CO)_12_] (*x* = 4.41), [NBu_4_]_2_[Pt_6–*x*_Ni_*x*_(CO)_12_] (*x* = 5.78), [NBu_4_]_2_[Pt_6–*x*_Ni_*x*_(CO)_12_] (*x* = 5.90), [NBu_4_]_4_[Pt_6–*x*_Ni_*x*_(CO)_12_][Cl_1.77_Br_0.23_] (*x* = 2.53), [NBu_4_]_2_[Pt_9_(CO)_18_]**·**thf, and [NBu_4_]_2_[Pt_6_(CO)_12_] are reported in Table S5 in the Supporting Information. The diffraction experiments were carried out on a Bruker APEX II diffractometer equipped with a PHOTON2 detector using Mo Kα radiation. Data were corrected for Lorentz–polarization and absorption effects (empirical absorption correction with SADABS).^46^ Structures were solved by direct methods and refined by full-matrix least squares on the basis of all data using *F*^2^.^47^ Hydrogen atoms were fixed at calculated positions and refined by a riding model. All non-hydrogen atoms were refined with anisotropic displacement parameters, unless otherwise stated. Further information and refinement details may be found in the Supporting Information.

### 4.13 Computational Details with Figures and Tables

Full geometry optimizations, optimizations with selected constrained internal coordinates, and single-point calculations were carried out *in vacuo* using the hybrid meta-GGA DFT functional TPSS0, with 25% HF exchange,^48^ in combination with Ahlrichs’ def-2 TZVP basis set, with relativistic ECP for Pt.^49^ The “restricted” approach was used in all cases. Calculations were performed with ORCA 4.2.0 software.^50^ The output, converted in .molden format, was elaborated with the software Multiwfn, version 3.5.^51^ Cartesian coordinates of the DFT-optimized structures are collected in a separate .xyz file.

## Supporting Information Available

IR spectra, ESI-MS spectra, NMR spectra, figures of the possible isomers of [Pt_6–*x*_Ni_*x*_(CO)_12_]^2–^ (*x* = 0–6), reaction and isomerization schemes, X-ray crystallographic studies, and computational details with figures (PDF)
Optimized coordinates in .xyz format (XYZ)

## Supplementary Material

ic1c00752_si_002.pdf

ic1c00752_si_003.xyz

## Accession Codes

CCDC 2067847–2067856 contain the supplementary crystallographic data for this paper. These data can be obtained free of charge via www.ccdc.cam.ac.uk/data_request/cif, or by emailing data_request@ccdc.cam.ac.uk, or by contacting The Cambridge Crystallographic Data Centre, 12 Union Road, Cambridge CB2 1EZ, UK; fax: +44 1223 336033.

## Dedication

In memory of Prof. Larry F. Dahl, a giant of cluster chemistry, whose work has been an inspiration for generations of inorganic chemists.
