# Supplementary material for: Heterometallic Ni–Pt Chini-Type Carbonyl Clusters: An Example of Molecular Random Alloy Clusters
Source: Inorg Chem. 2021 Jun 4;60(12):8811–25. doi: 10.1021/acs.inorgchem.1c00752 (PMC8277170; doi:10.1021/acs.inorgchem.1c00752)
Supplement: Supplementary file 1 — ic1c00752_si_002.pdf [file ic1c00752_si_002.pdf]

Supporting Information for

Heterometallic Ni-Pt Chini-Type Carbonyl Clusters: An Example of Molecular Random Alloy Clusters

Cristiana Cesari,[†] Beatrice Berti,[†] Marco Bortoluzzi,[‡] Cristina Femoni,[†] Maria Carmela Iapalucci,[†] and Stefano Zacchini*[†]

[†] Dipartimento di Chimica Industriale "Toso Montanari", Università di Bologna, Viale Risorgimento 4 - 40136 Bologna, Italy. Fax: +39 0512093690, E-mail: ***stefano.zacchini@unibo.it***

[‡] Dipartimento di Scienze Molecolari e Nanosistemi, Ca' Foscari University of Venice, Via Torino 155 – 30175 Mestre (Ve), Italy

	Page/s
IR spectra	S2-S8
ESI-MS spectra	S9-S42
NMR spectra	S43-S49
Possible isomers of $[Pt_{6-x}Ni_x(CO)_{12}]^{2-}$ (x = 0 – 6)	S50
Triangle exchange reactions between $[Pt_{6-x}Ni_x(CO)_{12}]^{2-}$ (x = 0 – 6) clusters	S51-S58
Isomerization by CO migration of $[Pt_{6-x}Ni_x(CO)_{12}]^{2-}$ (x = 2 – 4)	S59-S60
Isomerization by triangle rotation of $[Pt_{6-x}Ni_x(CO)_{12}]^{2-}$ (x = 2 – 4)	S61
X-Ray crystallographic study	S62-S67
Computational details with figures and tables	S68-S72
References	S73

Figure S1

IR spectrum (ν_{CO} region) recorded in thf of $[NBu_4]_2[Ni_6(CO)_{12}]$.

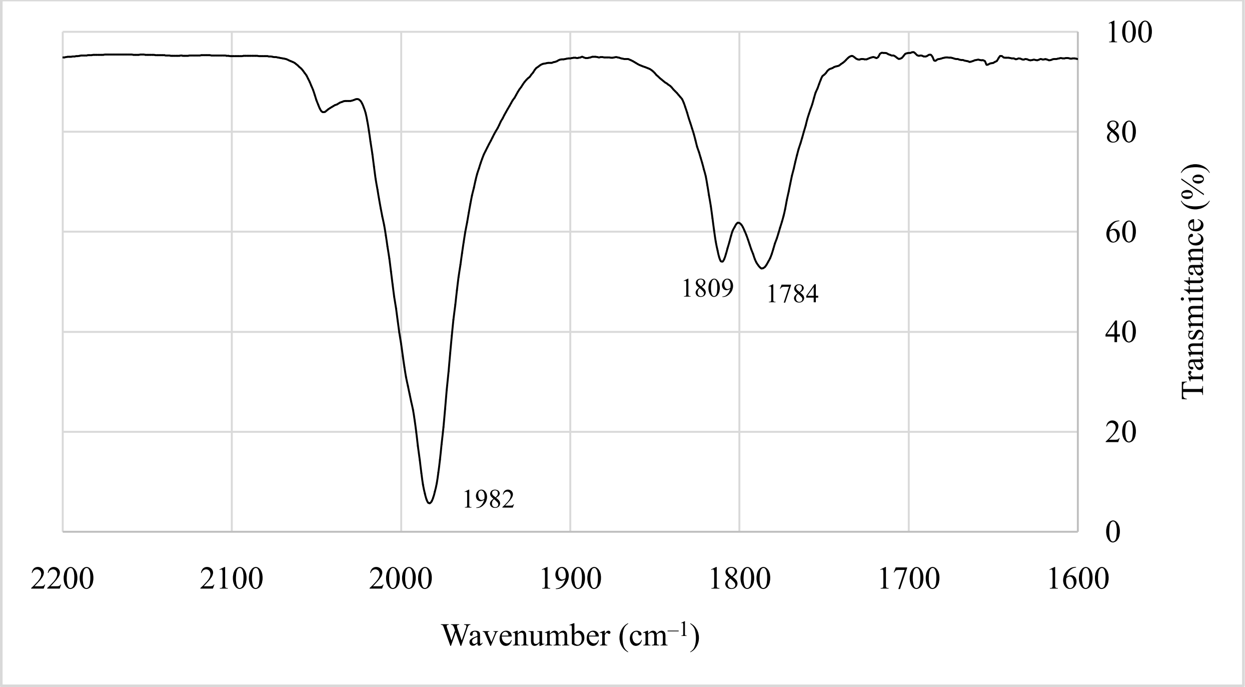

Figure S2

IR spectrum (ν_{CO} region) recorded in thf of $[NBu_4]_2[Ni_9(CO)_{18}]$.

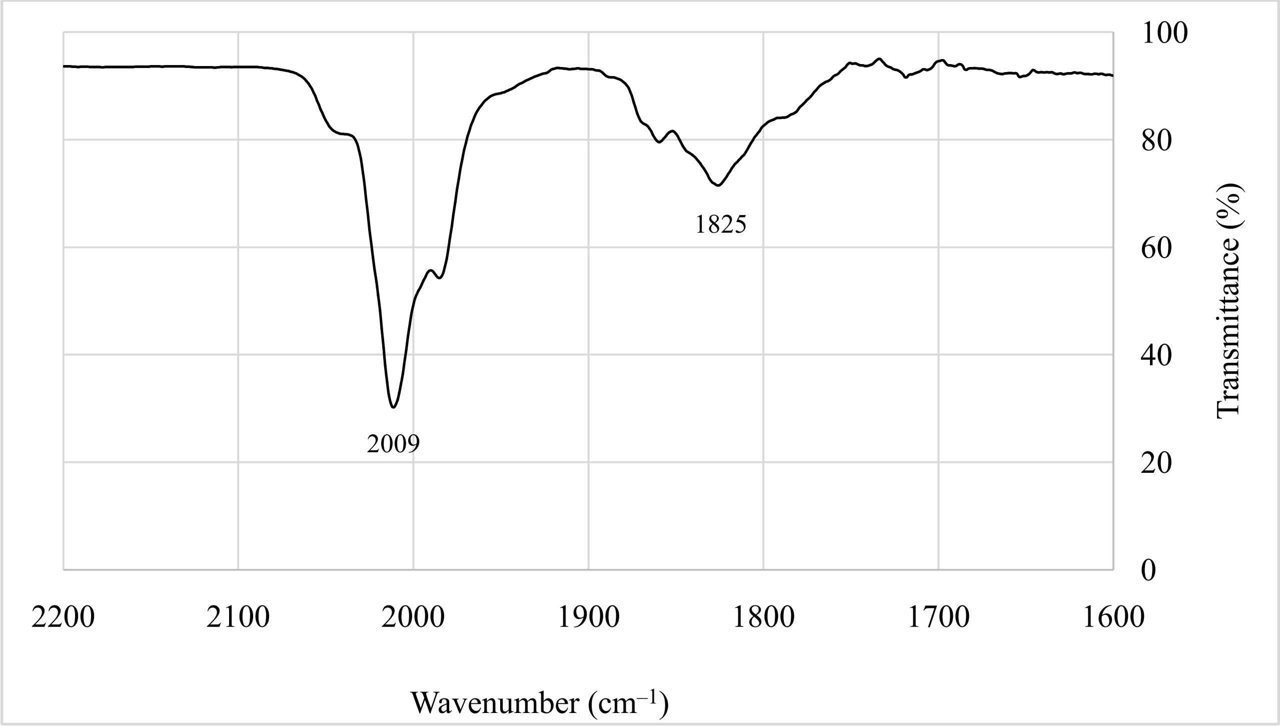

Figure S3

IR spectrum (ν_{CO} region) recorded in thf of $[NBu_4]_2[Pt_6(CO)_{12}]$.

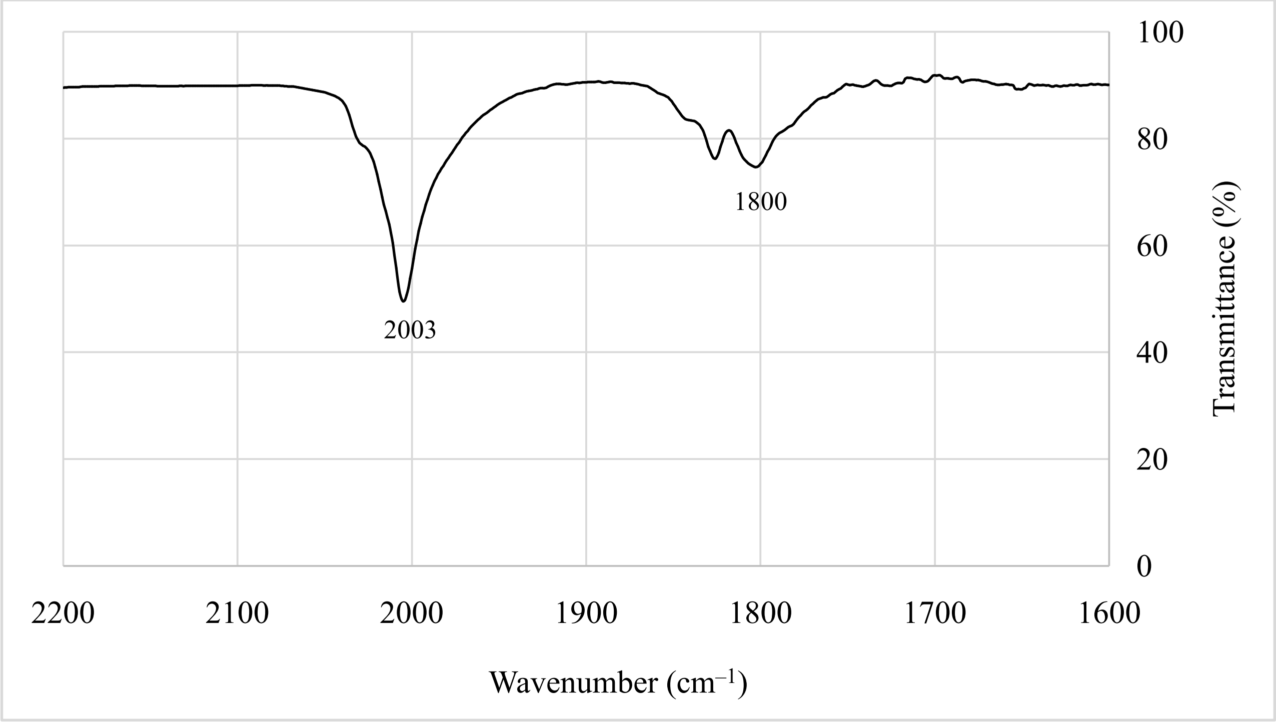

Figure S4

IR spectrum (ν_{CO} region) recorded in thf of $[NBu_4]_2[Pt_9(CO)_{18}]$.

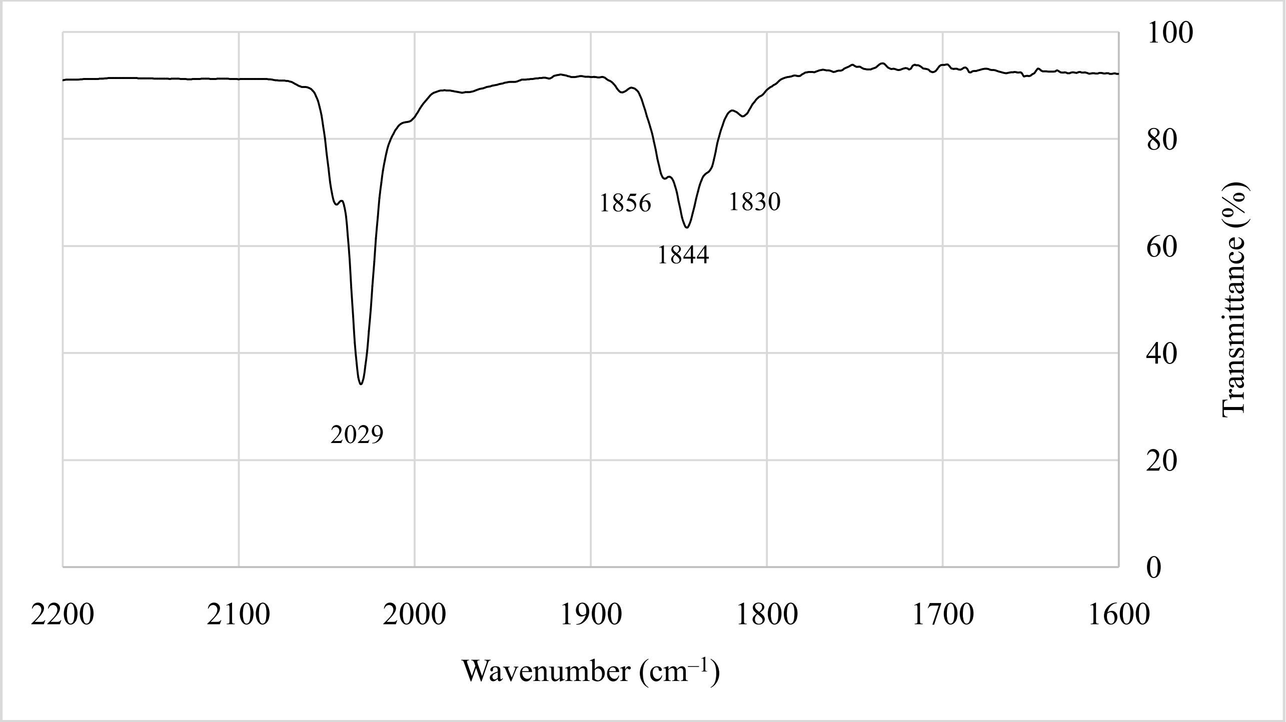

Figure S5

IR spectrum (ν_{CO} region) recorded in thf of $[NBu_4]_2[Pt_{12}(CO)_{24}]$.

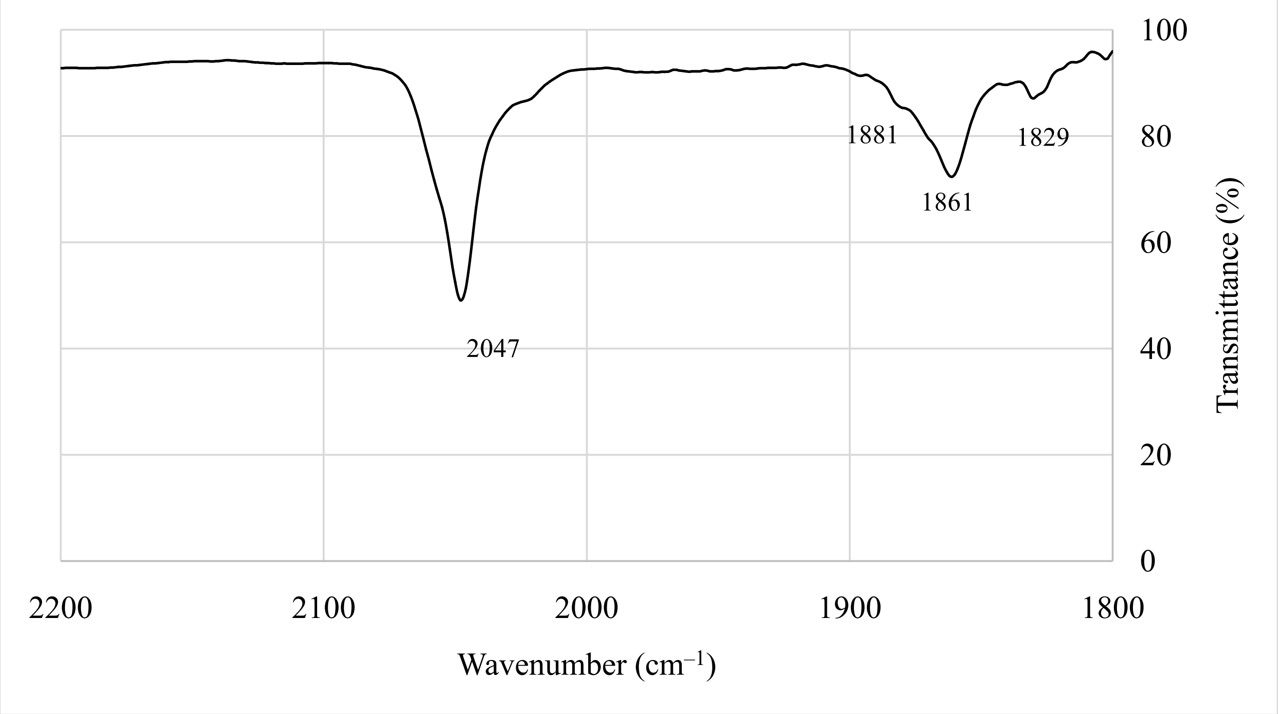

Figure S6

IR spectrum (ν_{CO} region) recorded in thf of the raw reaction mixture of $[NBu_4]_2[Pt_6(CO)_{12}]$ and $[NBu_4]_2[Ni_6(CO)_{12}]$ (1:1 molar ratio).

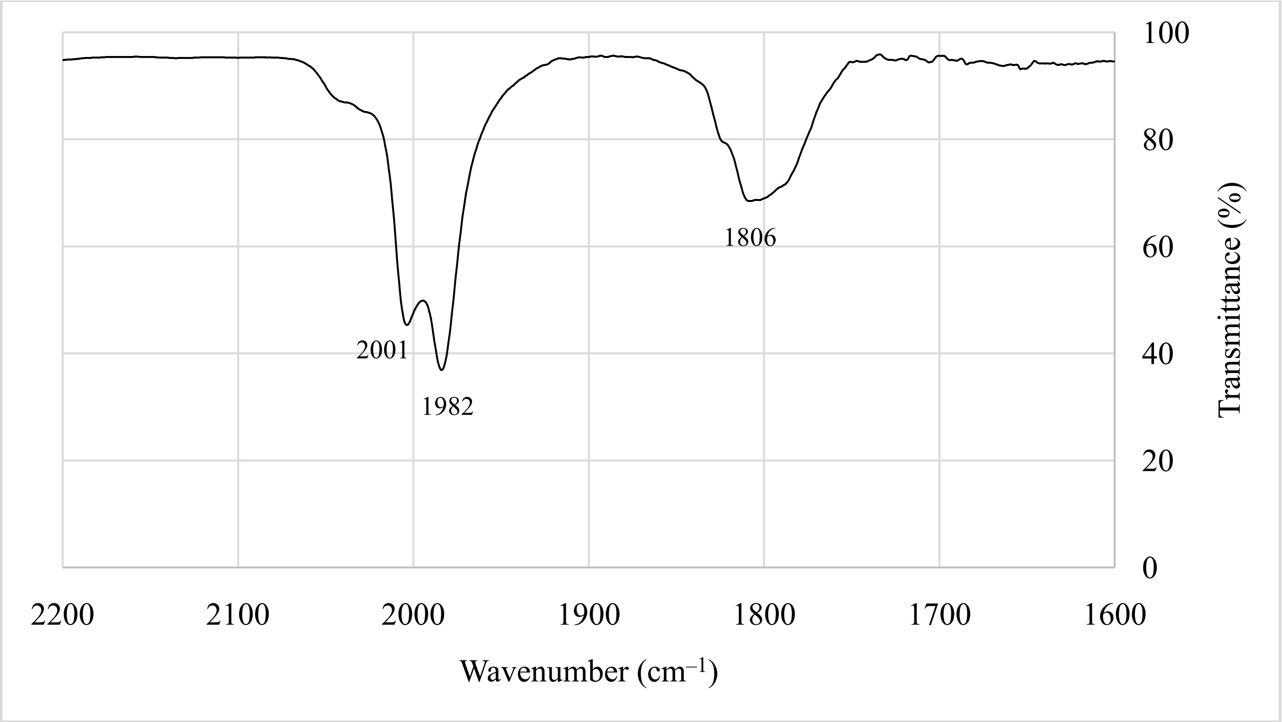

Figure S7

IR spectrum (ν_{CO} region) recorded in CH_3CN after work-up of the reaction mixture of $[NBu_4]_2[Pt_6(CO)_{12}]$ and $[NBu_4]_2[Ni_6(CO)_{12}]$ (1:1 molar ratio).

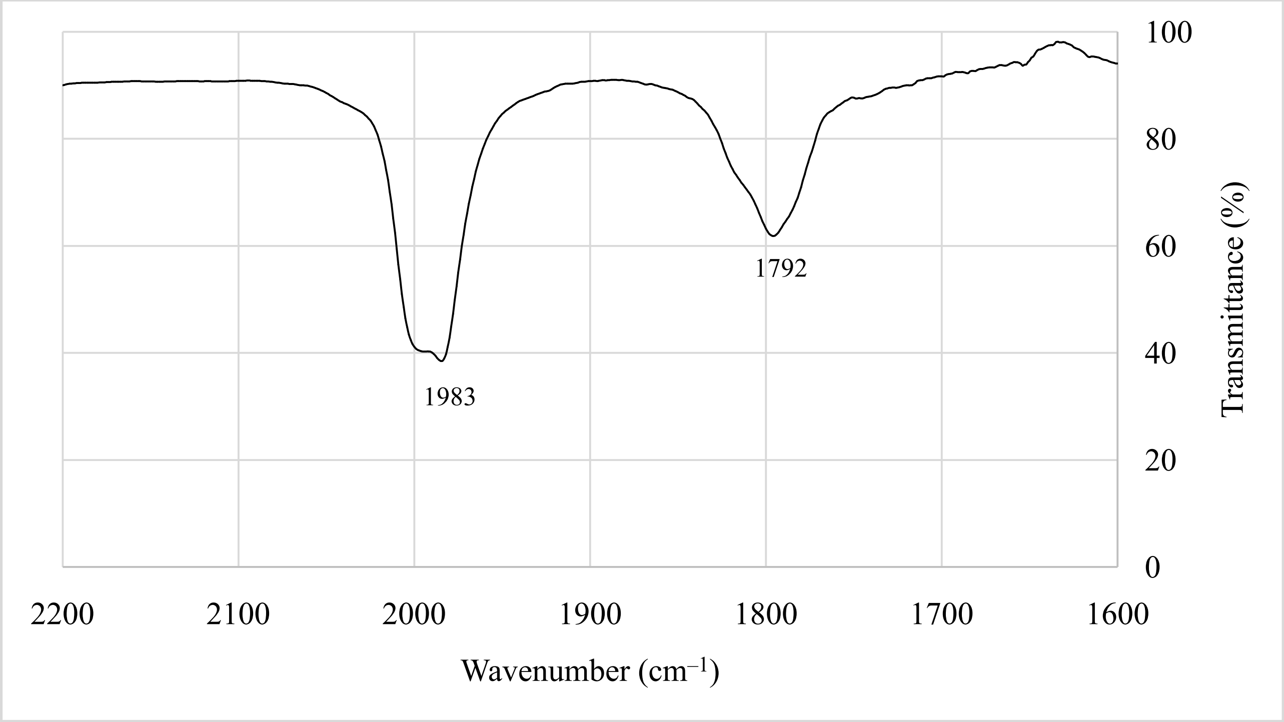

Figure S8

IR spectrum (ν_{CO} region) recorded in thf of the raw reaction mixture of $[NBu_4]_2[Pt_6(CO)_{12}]$ and $[NBu_4]_2[Ni_6(CO)_{12}]$ (1:2 molar ratio).

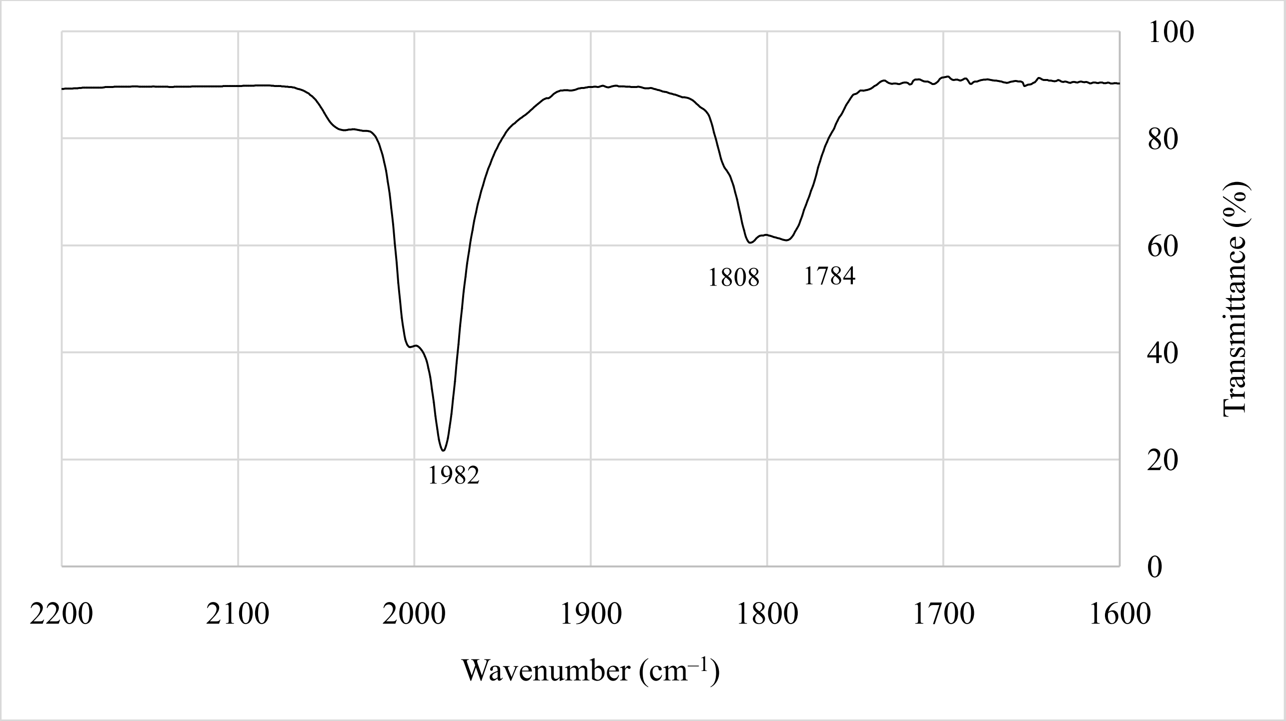

Figure S9

IR spectrum (ν_{CO} region) recorded in CH_3CN after work-up of the reaction mixture of $[NBu_4]_2[Pt_6(CO)_{12}]$ and $[NBu_4]_2[Ni_6(CO)_{12}]$ (1:2 molar ratio).

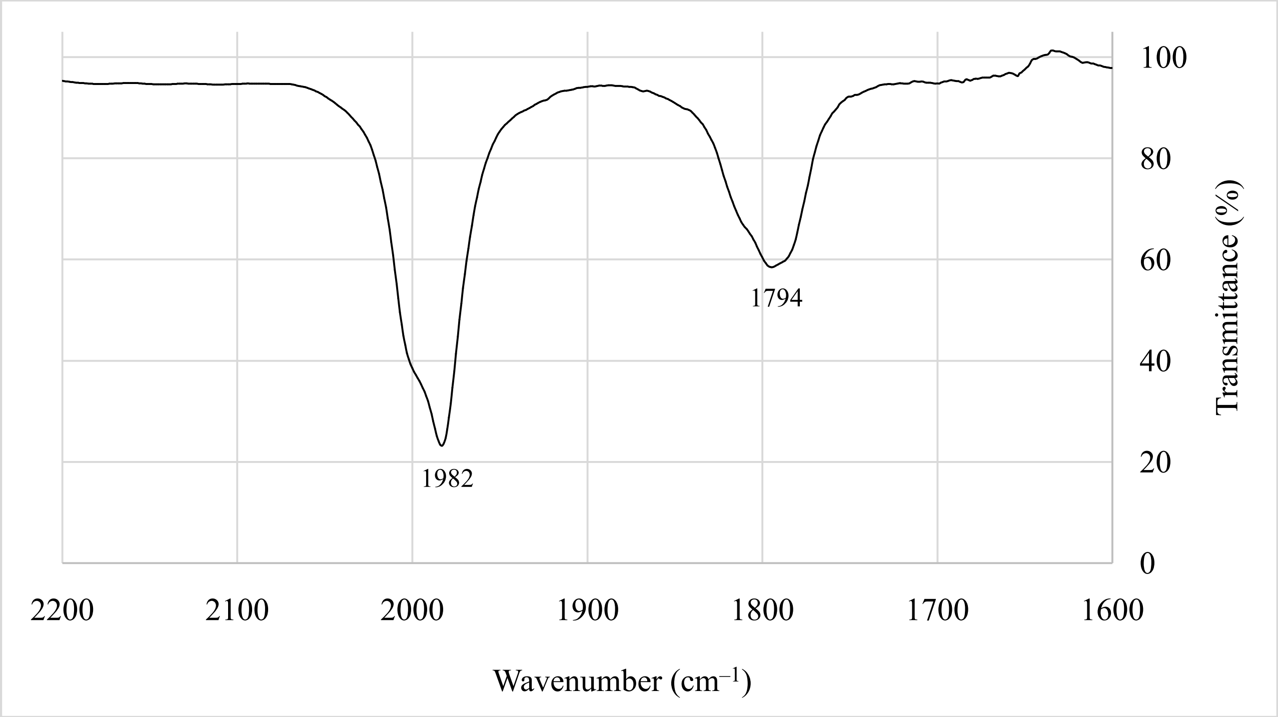

Figure S10

IR spectrum (ν_{CO} region) recorded in thf of the raw reaction mixture of $[NBu_4]_2[Pt_6(CO)_{12}]$ and $[NBu_4]_2[Ni_6(CO)_{12}]$ (2:1 molar ratio).

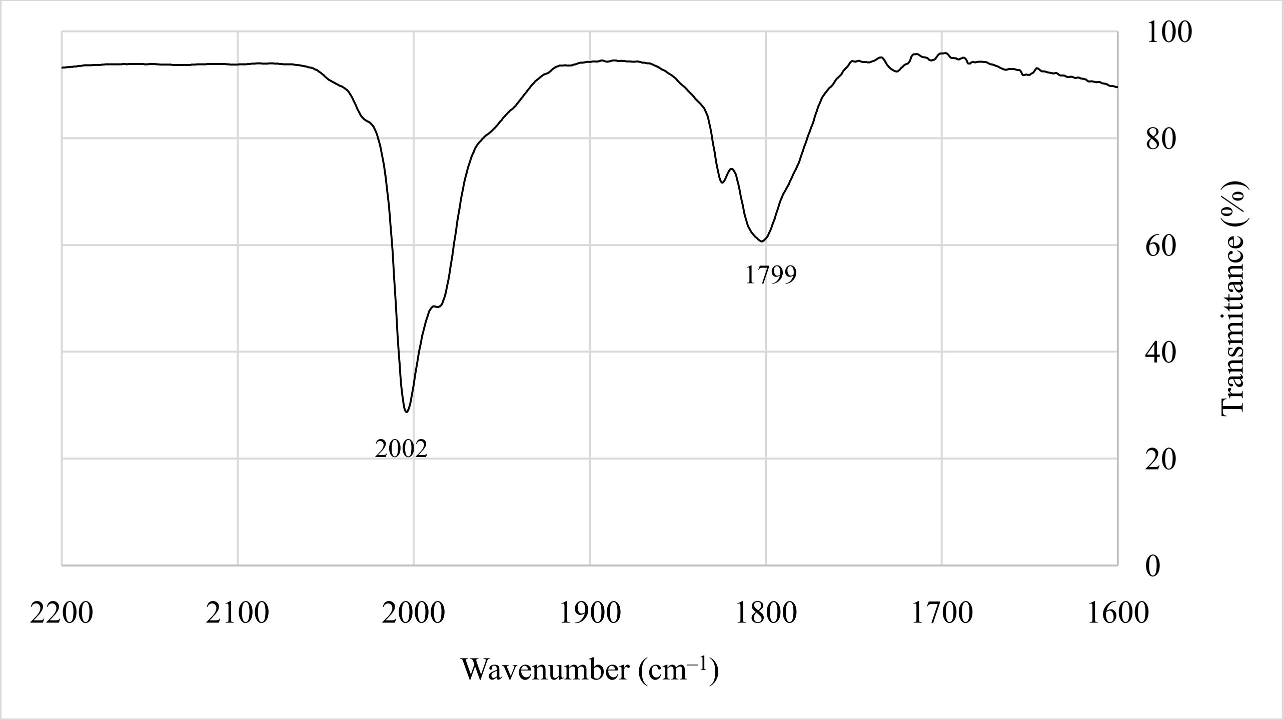

Figure S11

IR spectrum (ν_{CO} region) recorded in CH_3CN after work-up of the reaction mixture of $[NBu_4]_2[Pt_6(CO)_{12}]$ and $[NBu_4]_2[Ni_6(CO)_{12}]$ (2:1 molar ratio).

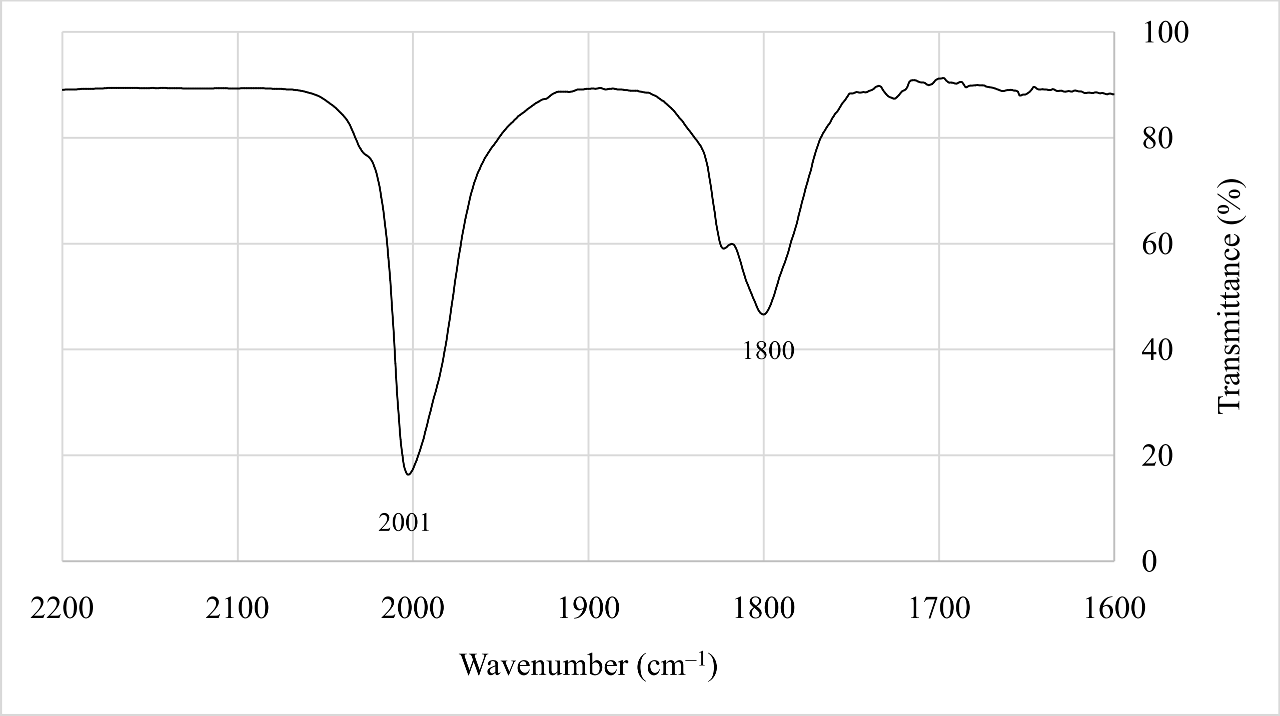

Figure S12

IR spectrum (ν_{CO} region) recorded in thf of the raw reaction mixture of $[NBu_4]_2[Pt_9(CO)_{18}]$ and $[NBu_4]_2[Ni_6(CO)_{12}]$ (1:1 molar ratio).

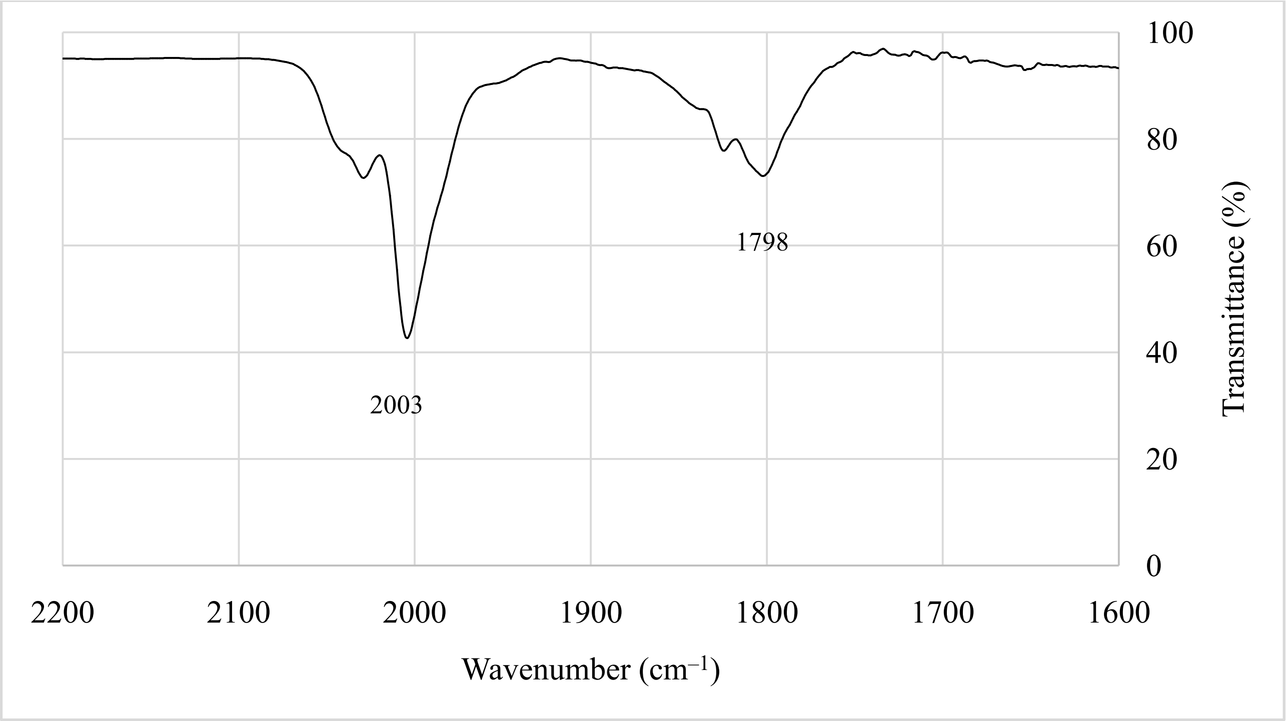

Figure S13

IR spectrum (ν_{CO} region) recorded in CH_3CN after work-up of the reaction mixture of $[NBu_4]_2[Pt_9(CO)_{18}]$ and $[NBu_4]_2[Ni_6(CO)_{12}]$ (1:1 molar ratio).

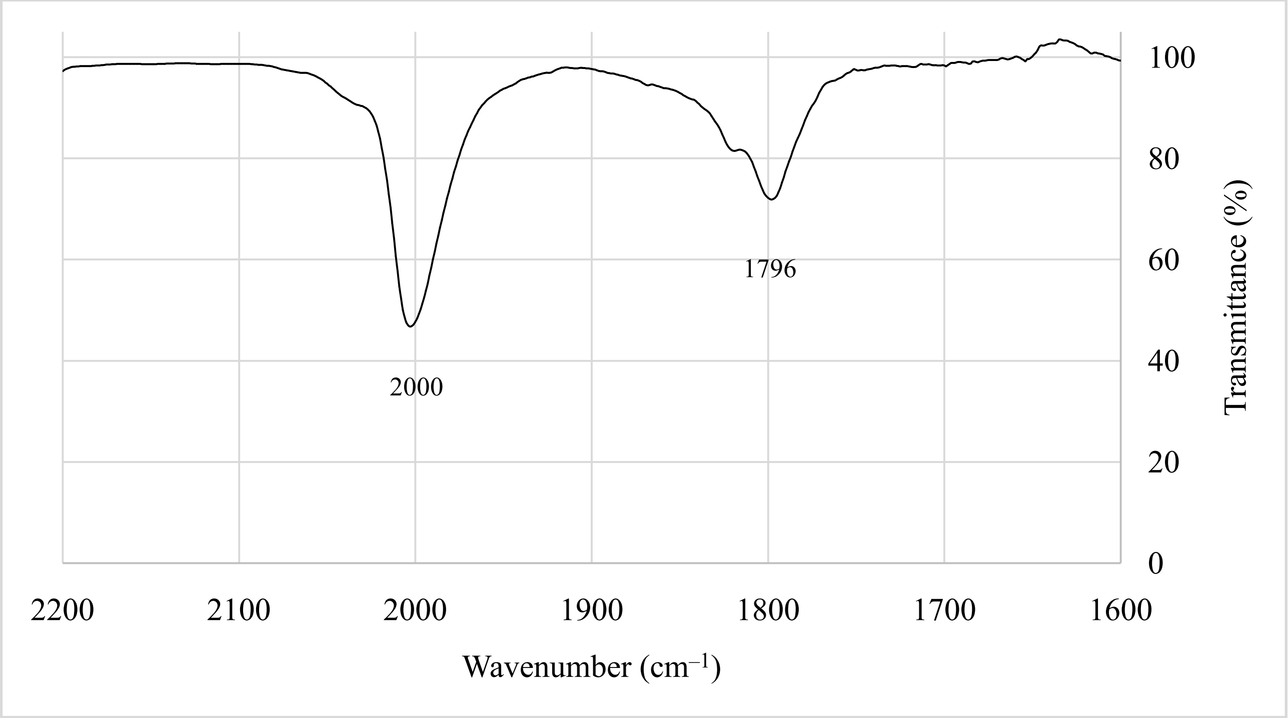

Figure S14

ESI-MS spectrum in CH_3CN (ES–) of $[NBu_4]_2[Pt_{6-x}Ni_x(CO)_{12}]$ (x = 1.25).

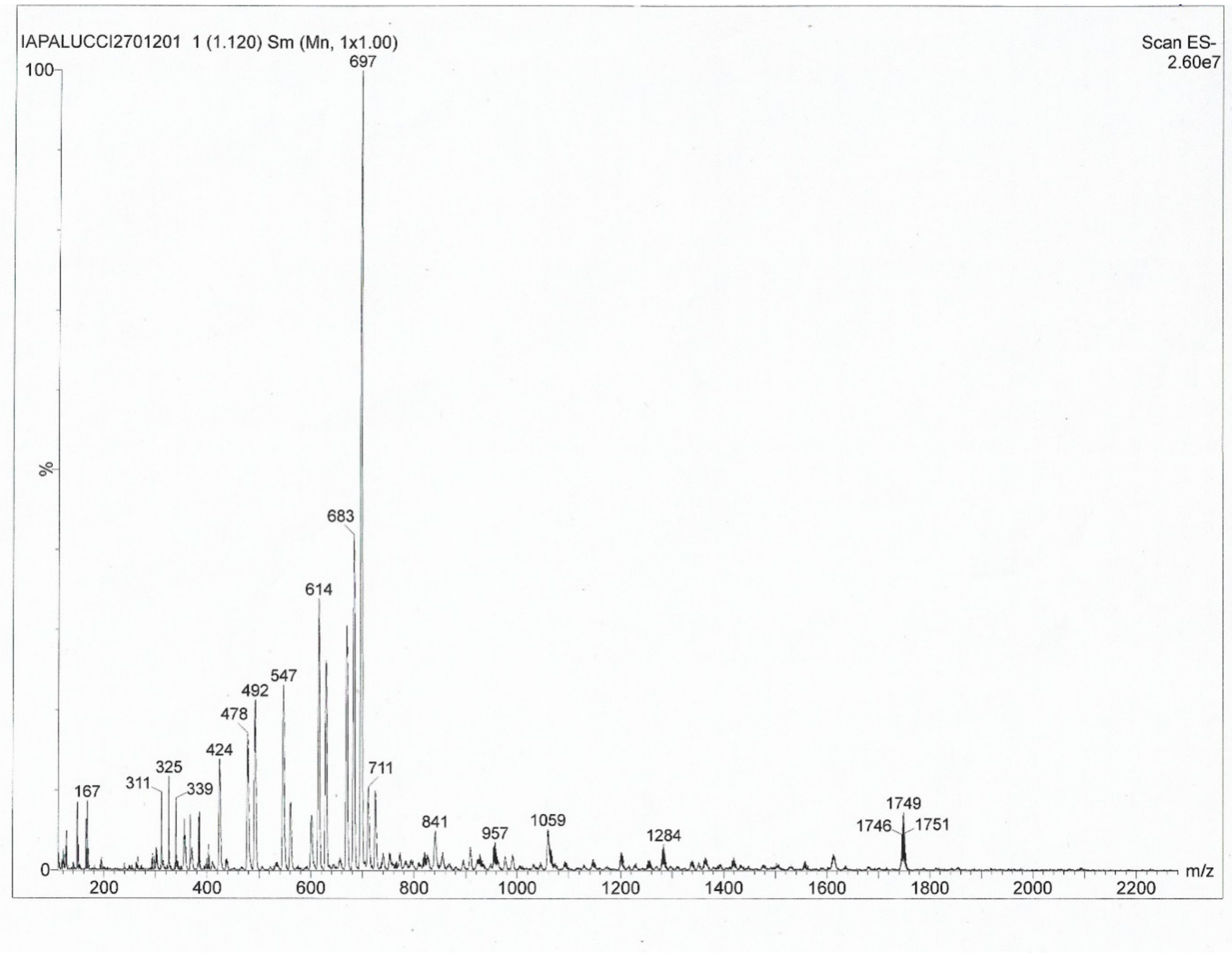

Table S1

Peak assignment of the ESI-MS spectrum (ES–) of $[NBu_4]_2[Pt_{6-x}Ni_x(CO)_{12}]$ (x = 1.25).

**m/z**	**Relative intensity**	**Ion**	**Code**	**Sum of the peaks intensities**
1749	10	$\{[Pt_6(CO)_{12}][NBu_4]\}^-$	Pt_6+NBu_4	10
1612	4	$\{[Pt_5Ni(CO)_{12}][NBu_4]\}^-$	Pt_5Ni+NBu_4	199
725	10	$[Pt_5Ni(CO)_{15}]^{2-}$	Pt_5Ni+3CO	
711	10	$[Pt_5Ni(CO)_{14}]^{2-}$	$PtNi_5$+2CO	
697	100	$[Pt_5Ni(CO)_{13}]^{2-}$	Pt_5Ni+1CO	
683	45	$[Pt_5Ni(CO)_{12}]^{2-}$	Pt_5Ni	
669	30	$[Pt_5Ni(CO)_{11}]^{2-}$	Pt_5Ni–1CO	
629	25	$[Pt_4Ni_2(CO)_{13}]^{2-}$	Pt_4Ni_2+1CO	65
614	35	$[Pt_4Ni_2(CO)_{12}]^{2-}$	Pt_4Ni_2	
601	5	$[Pt_4Ni_2(CO)_{11}]^{2-}$	Pt_4Ni_2–1CO	
561	8	$[Pt_3Ni_3(CO)_{13}]^{2-}$	Pt_3Ni_3+1CO	28
547	20	$[Pt_3Ni_3(CO)_{12}]^{2-}$	Pt_3Ni_3	
492	20	$[Pt_2Ni_4(CO)_{13}]^{2-}$	Pt_2Ni_4+1CO	35
478	15	$[Pt_2Ni_4(CO)_{12}]^{2-}$	Pt_2Ni_4	
424	15	$[PtNi_5(CO)_{13}]^{2-}$	$PtNi_5$+1CO	15

Figure S15

Isotopic pattern of the peak at m/z 424 of the ESI-MS spectrum in CH_3CN (ES–) of $[NBu_4]_2[Pt_{6-x}Ni_x(CO)_{12}]$ (x = 1.25). Upper traces: calculated isotopic patter for $[PtNi_5(CO)_{13}]^{2-}$. Lower trace: experimental isotopic pattern.

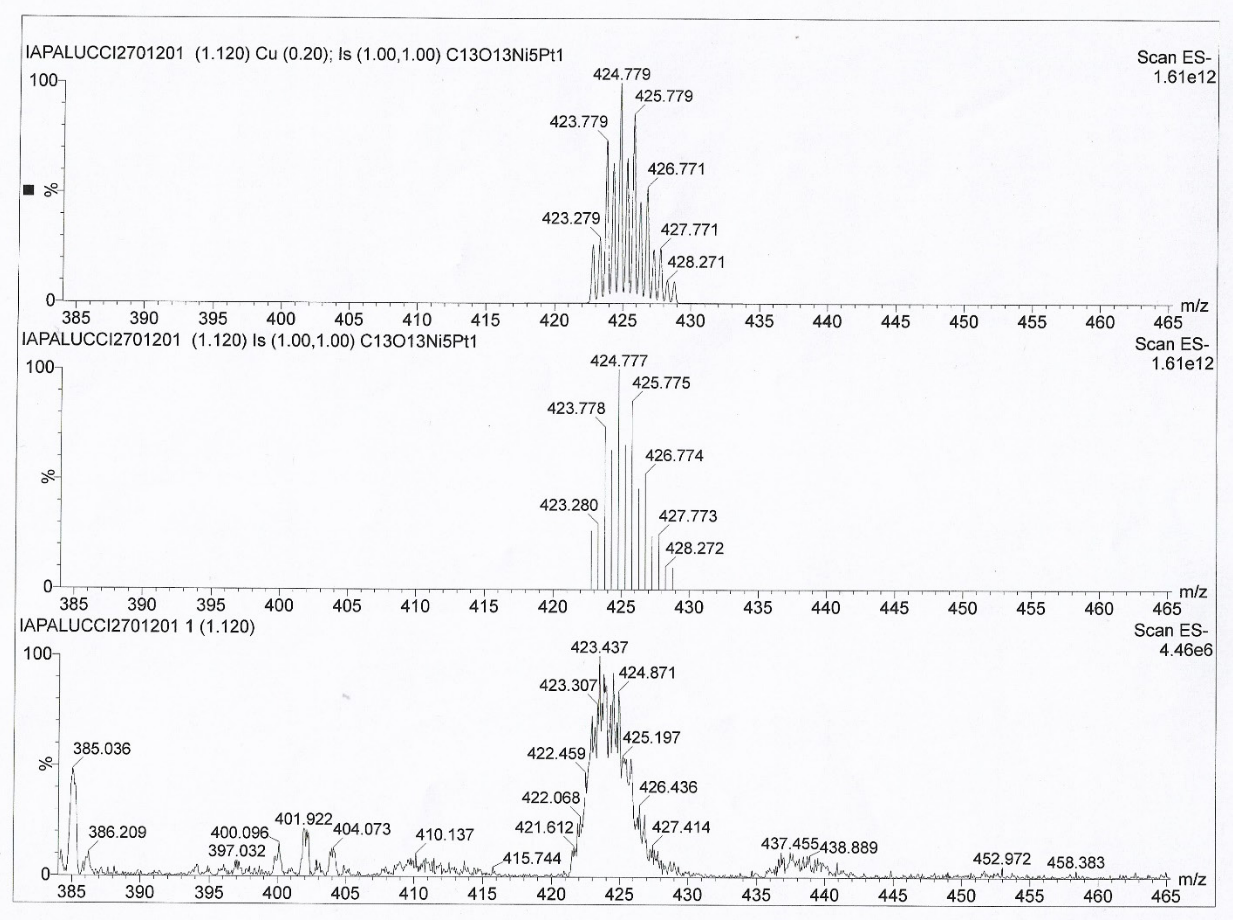

Figure S16

Isotopic pattern of the peak at m/z 492 of the ESI-MS spectrum in CH_3CN (ES–) of $[NBu_4]_2[Pt_{6-x}Ni_x(CO)_{12}]$ (x = 1.25). Upper traces: calculated isotopic patter for $[Pt_2Ni_4(CO)_{13}]^{2-}$. Lower trace: experimental isotopic pattern.

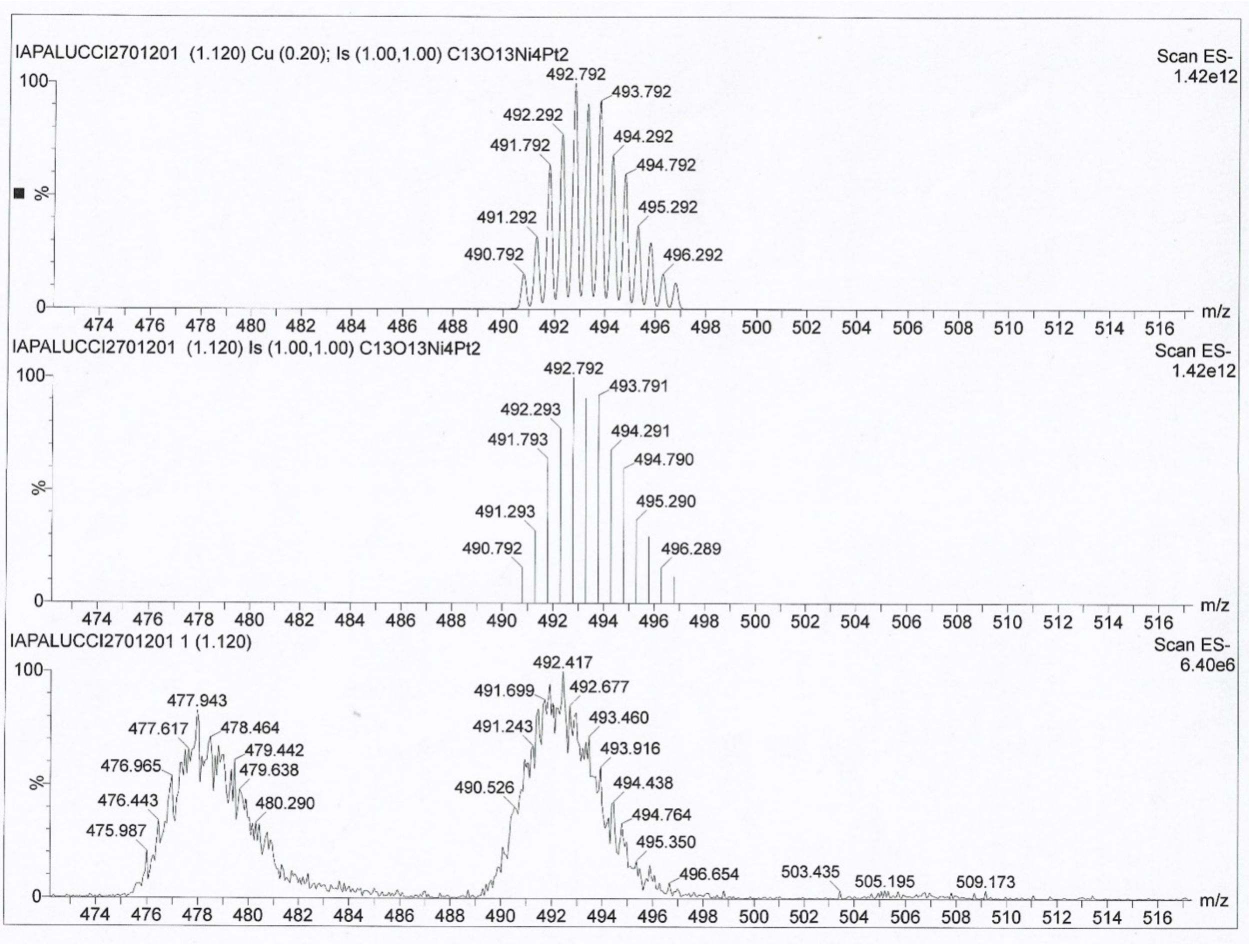

Figure S17

Isotopic pattern of the peak at m/z 547 of the ESI-MS spectrum in CH_3CN (ES–) of $[NBu_4]_2[Pt_{6-x}Ni_x(CO)_{12}]$ (x = 1.25). Upper traces: calculated isotopic patter for $[Pt_3Ni_3(CO)_{12}]^{2-}$. Lower trace: experimental isotopic pattern.

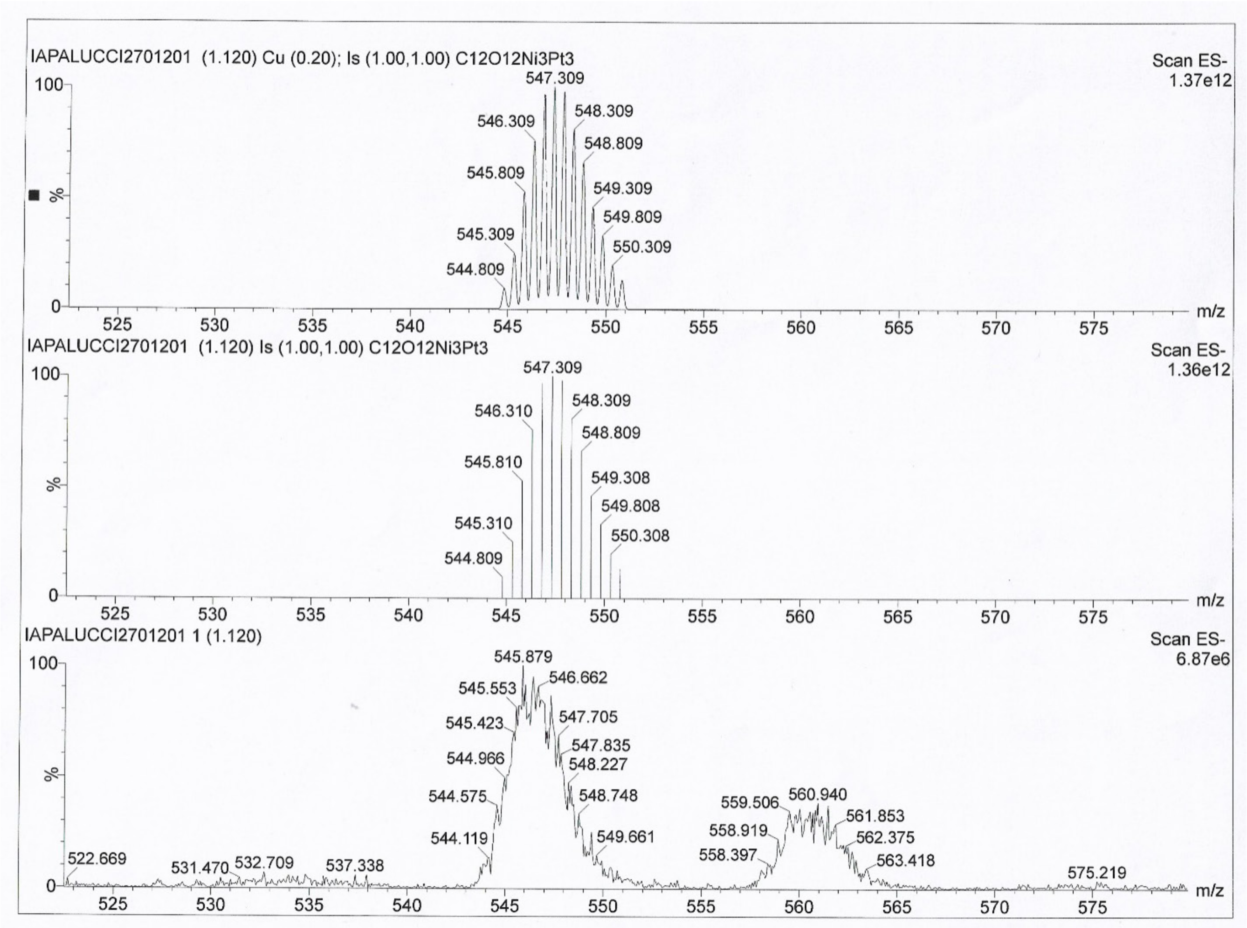

Figure S18

Isotopic pattern of the peak at m/z 614 of the ESI-MS spectrum in CH_3CN (ES–) of $[NBu_4]_2[Pt_{6-x}Ni_x(CO)_{12}]$ (x = 1.25). Upper traces: calculated isotopic patter for $[Pt_4Ni_2(CO)_{12}]^{2-}$. Lower trace: experimental isotopic pattern.

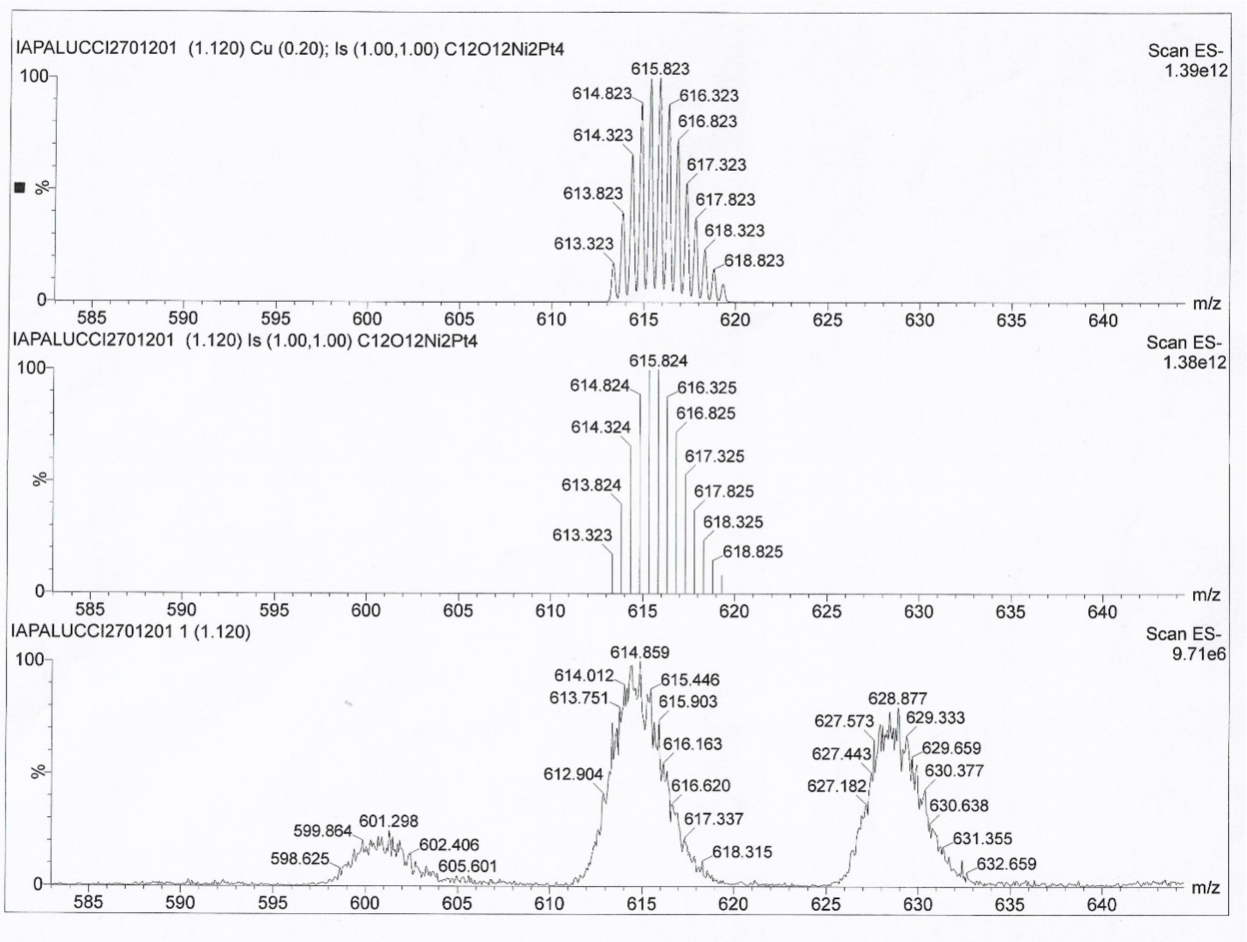

Figure S19

Isotopic pattern of the peak at m/z 683 of the ESI-MS spectrum in CH_3CN (ES–) of $[NBu_4]_2[Pt_{6-x}Ni_x(CO)_{12}]$ (x = 1.25). Upper traces: calculated isotopic patter for $[Pt_5Ni(CO)_{12}]^{2-}$. Lower trace: experimental isotopic pattern.

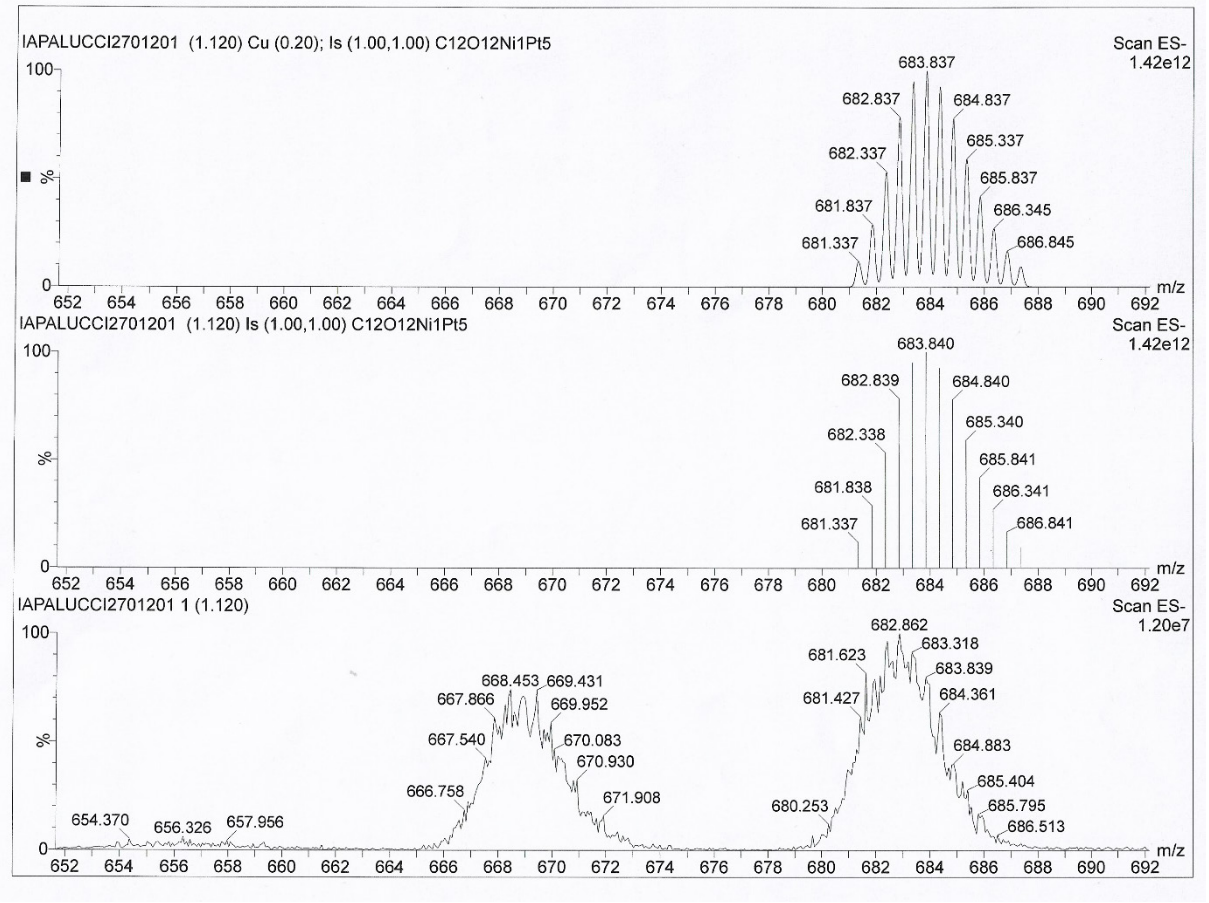

Figure S20

Isotopic pattern of the peak at m/z 697 of the ESI-MS spectrum in CH_3CN (ES–) of $[NBu_4]_2[Pt_{6-x}Ni_x(CO)_{12}]$ (x = 1.25). Upper traces: calculated isotopic patter for $[Pt_5Ni(CO)_{13}]^{2-}$. Lower trace: experimental isotopic pattern.

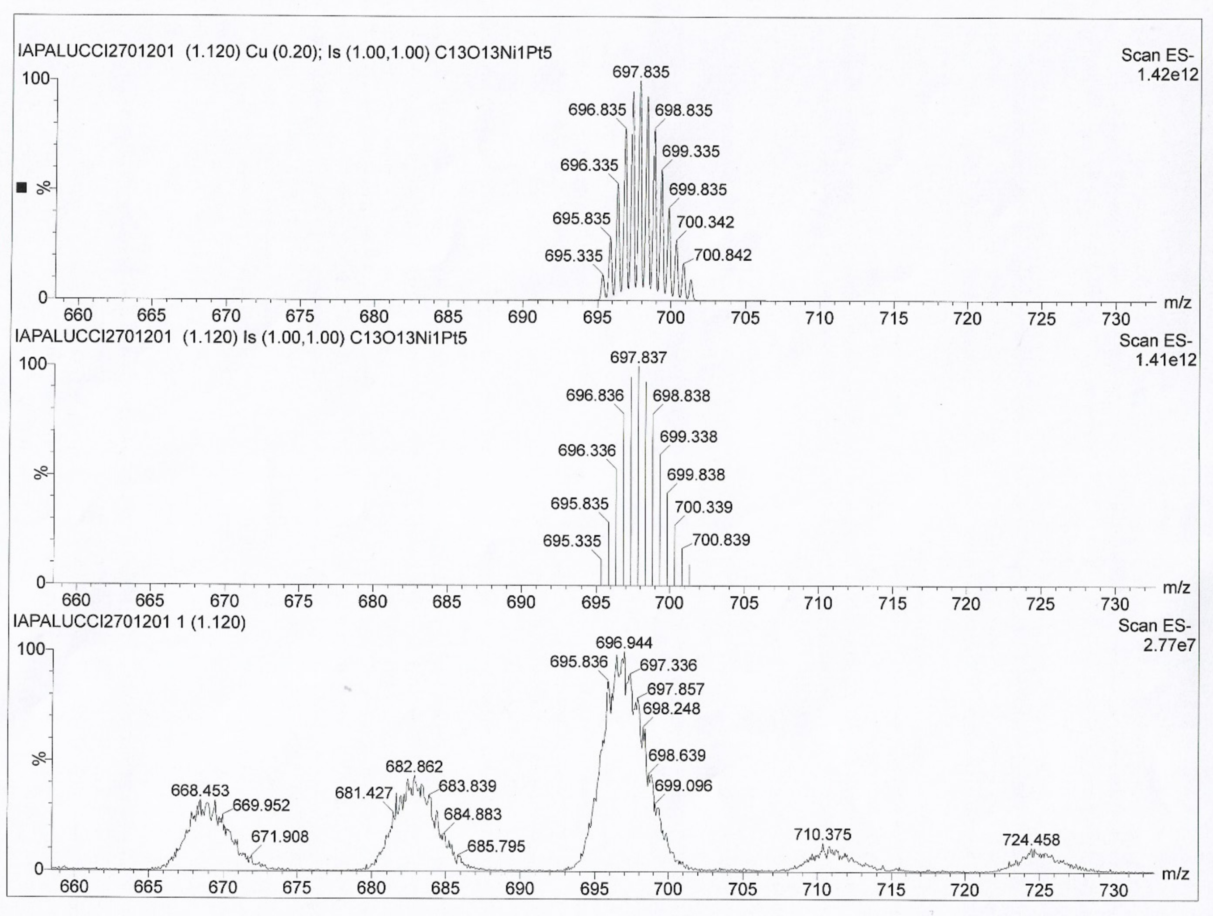

Figure S21

Isotopic pattern of the peak at m/z 1749 of the ESI-MS spectrum in CH_3CN (ES–) of $[NBu_4]_2[Pt_{6-x}Ni_x(CO)_{12}]$ (x = 1.25). Upper traces: calculated isotopic patter for $\{[Pt_6(CO)_{12}][NBu_4]\}^-$. Lower trace: experimental isotopic pattern.

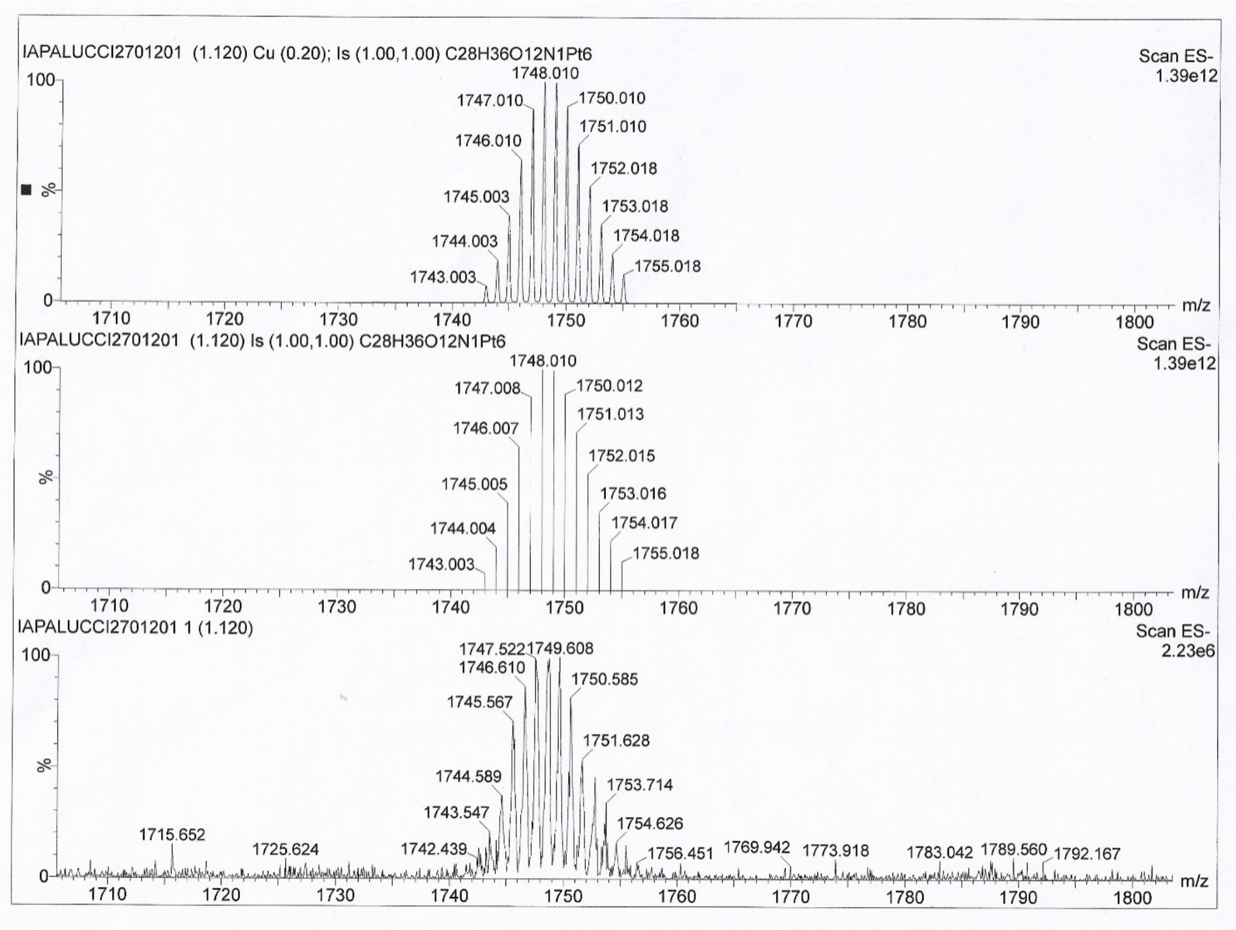

Figure S22

ESI-MS spectrum in CH_3CN (ES–) of $[NBu_4]_2[Pt_9(CO)_{18}]$ + 1.2$[NBu_4]_2[Ni_6(CO)_{12}]$ after work-up.

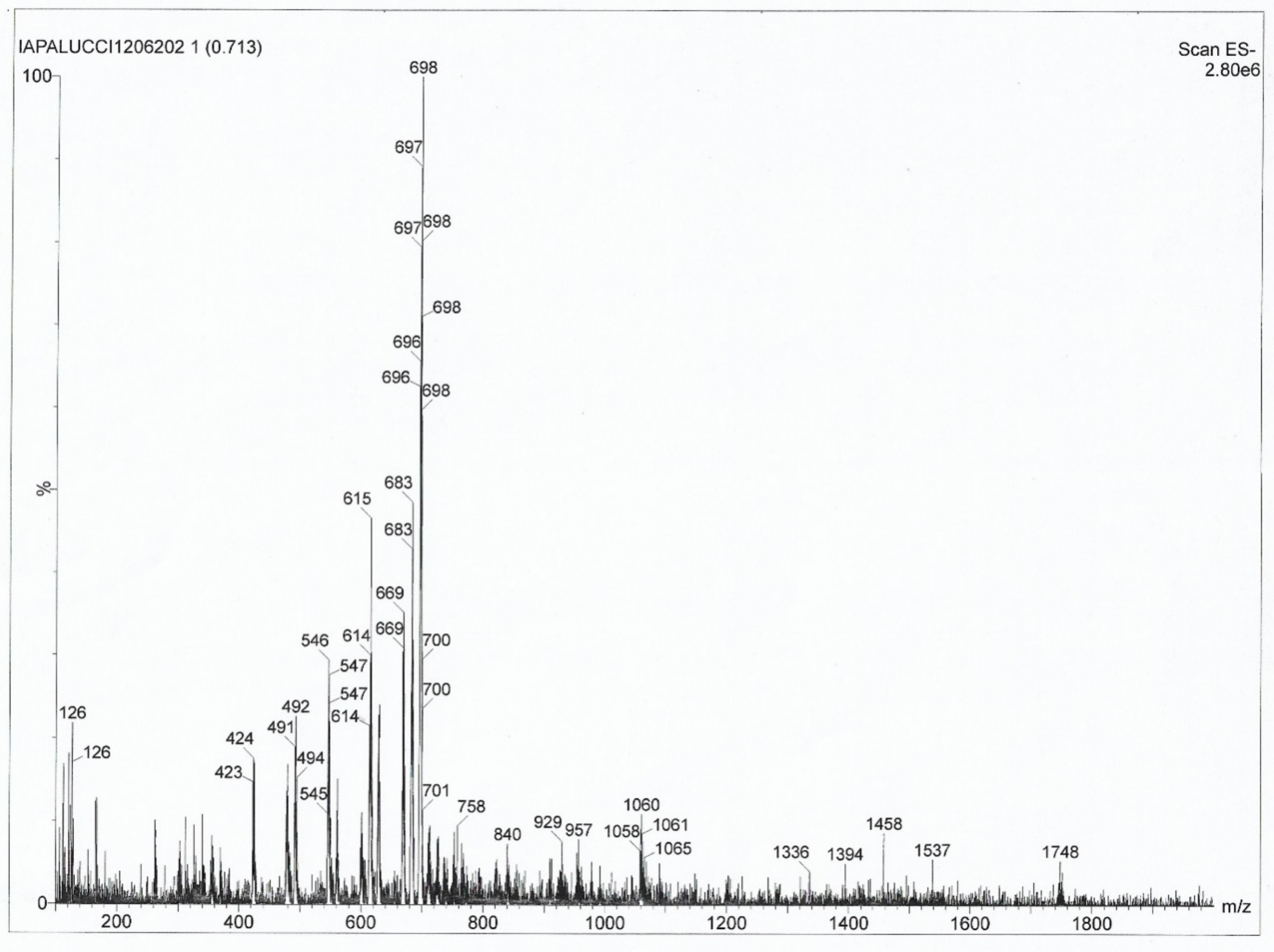

Table S2

Peak assignment of the ESI-MS spectrum (ES–) of $[NBu_4]_2[Pt_9(CO)_{18}]$ + 1.2$[NBu_4]_2[Ni_6(CO)_{12}]$ after work-up.

m/z	Relative intensity	Ion	Code	Sum of the peaks intensities
1749	5	$\{[Pt_6(CO)_{12}][NBu_4]\}^{-}$	Pt_6+NBu_4	5
698	100	$[Pt_5Ni(CO)_{13}]^{2-}$	Pt_5Ni+1CO	185
683	50	$[Pt_5Ni(CO)_{12}]^{2-}$	Pt_5Ni	
669	35	$[Pt_5Ni(CO)_{11}]^{2-}$	Pt_5Ni–1CO	
629	25	$[Pt_4Ni_2(CO)_{13}]^{2-}$	Pt_4Ni_2+1CO	85
615	50	$[Pt_4Ni_2(CO)_{12}]^{2-}$	Pt_4Ni_2	
601	10	$[Pt_4Ni_2(CO)_{11}]^{2-}$	Pt_4Ni_2–1CO	
561	15	$[Pt_3Ni_3(CO)_{13}]^{2-}$	Pt_3Ni_3+1CO	45
546	30	$[Pt_3Ni_3(CO)_{12}]^{2-}$	Pt_3Ni_3	
492	20	$[Pt_2Ni_4(CO)_{13}]^{2-}$	Pt_2Ni_4+1CO	35
478	15	$[Pt_2Ni_4(CO)_{12}]^{2-}$	Pt_2Ni_4	
424	18	$[PtNi_5(CO)_{13}]^{2-}$	$PtNi_5$+1CO	18

Figure S23

Isotopic pattern of the peak at m/z 546 of the ESI-MS spectrum in CH_3CN (ES–) of $[NBu_4]_2[Pt_9(CO)_{18}]$ + 1.2$[NBu_4]_2[Ni_6(CO)_{12}]$ after work-up. Upper traces: calculated isotopic patter for $[Pt_3Ni_3(CO)_{12}]^{2-}$. Lower trace: experimental isotopic pattern.

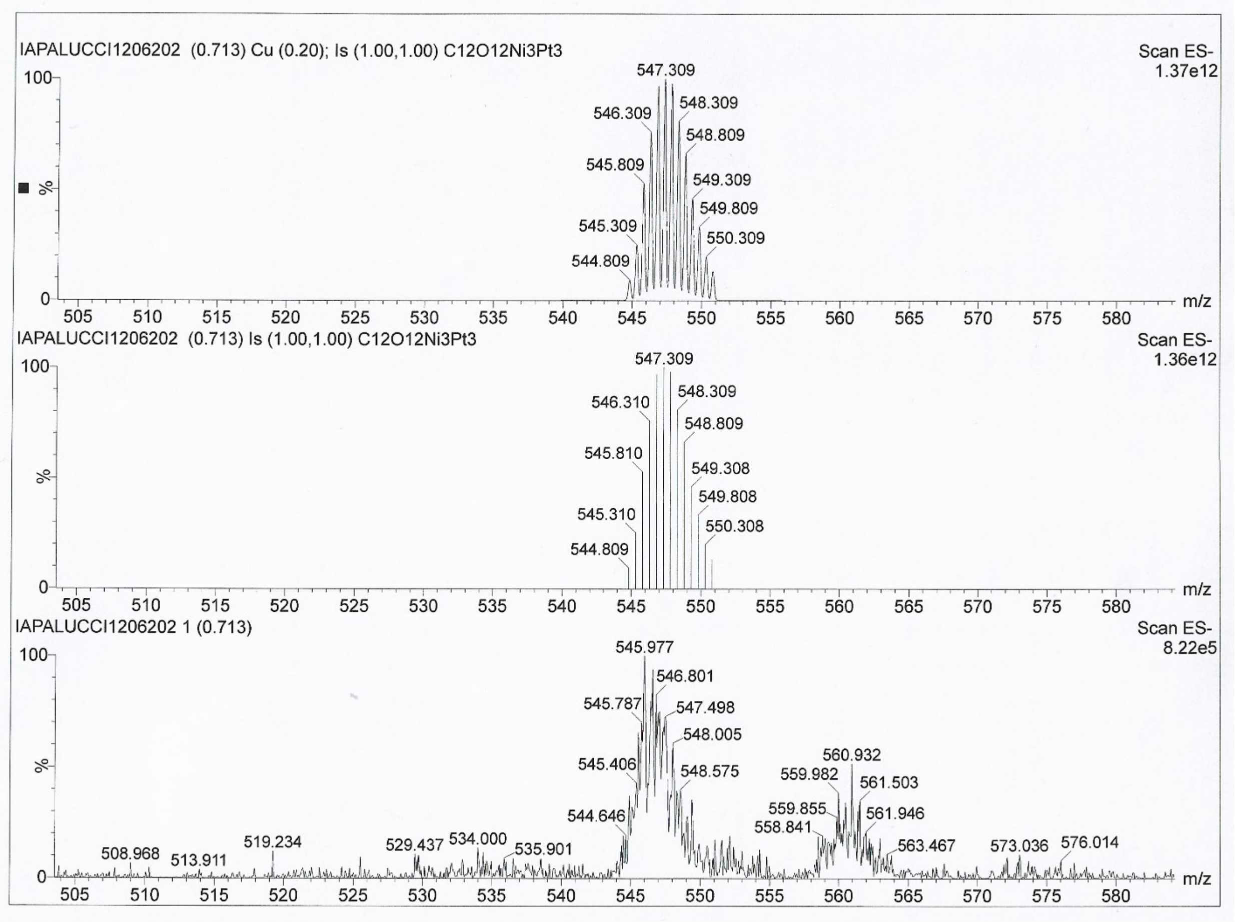

Figure S24

Isotopic pattern of the peak at m/z 615 of the ESI-MS spectrum in CH_3CN (ES–) of $[NBu_4]_2[Pt_9(CO)_{18}]$ + 1.2$[NBu_4]_2[Ni_6(CO)_{12}]$ after work-up. Upper traces: calculated isotopic patter for $[Pt_4Ni_2(CO)_{12}]^{2-}$. Lower trace: experimental isotopic pattern.

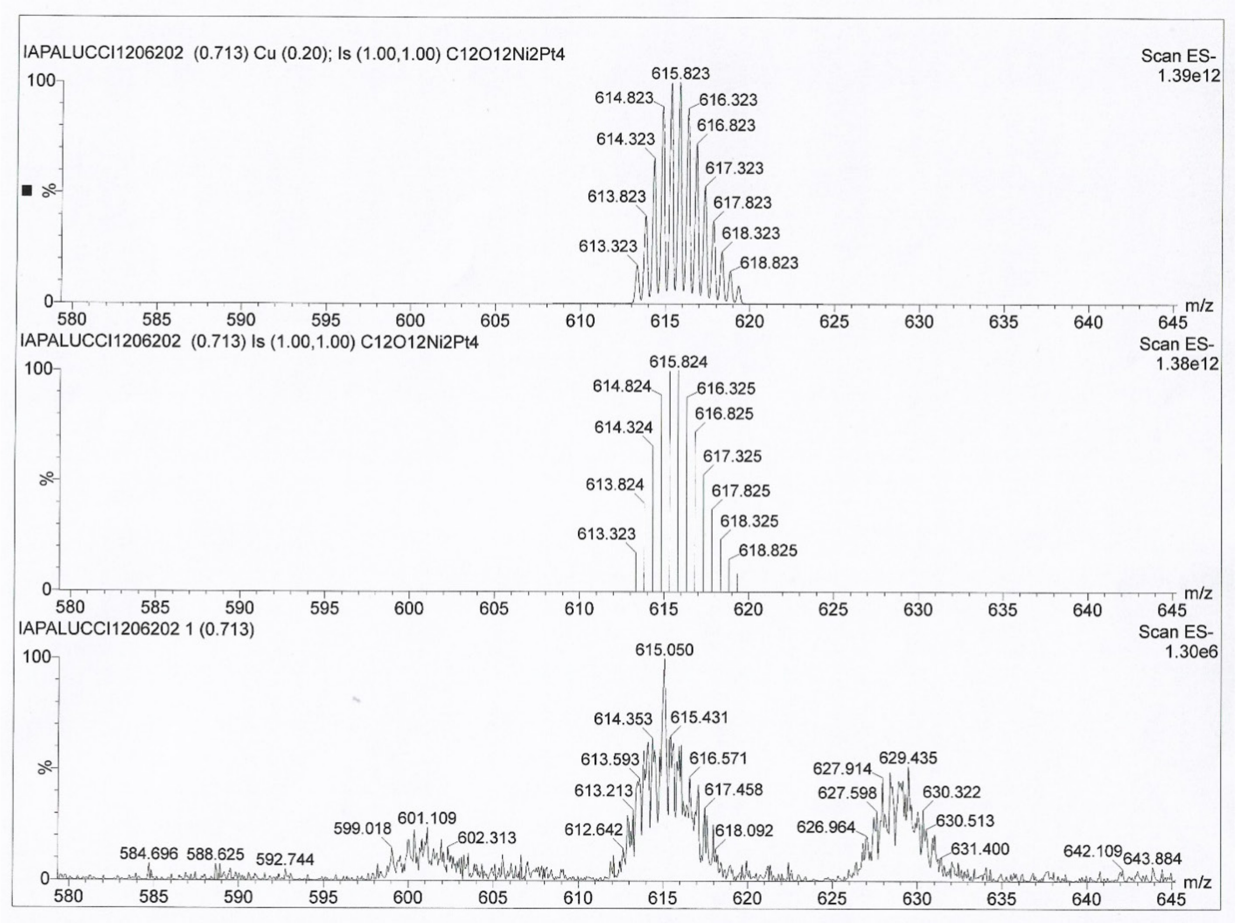

Figure S25

Isotopic pattern of the peak at m/z 683 of the ESI-MS spectrum in CH_3CN (ES–) of $[NBu_4]_2[Pt_9(CO)_{18}]$ + 1.2$[NBu_4]_2[Ni_6(CO)_{12}]$ after work-up. Upper traces: calculated isotopic patter for $[Pt_5Ni(CO)_{12}]^{2-}$. Lower trace: experimental isotopic pattern.

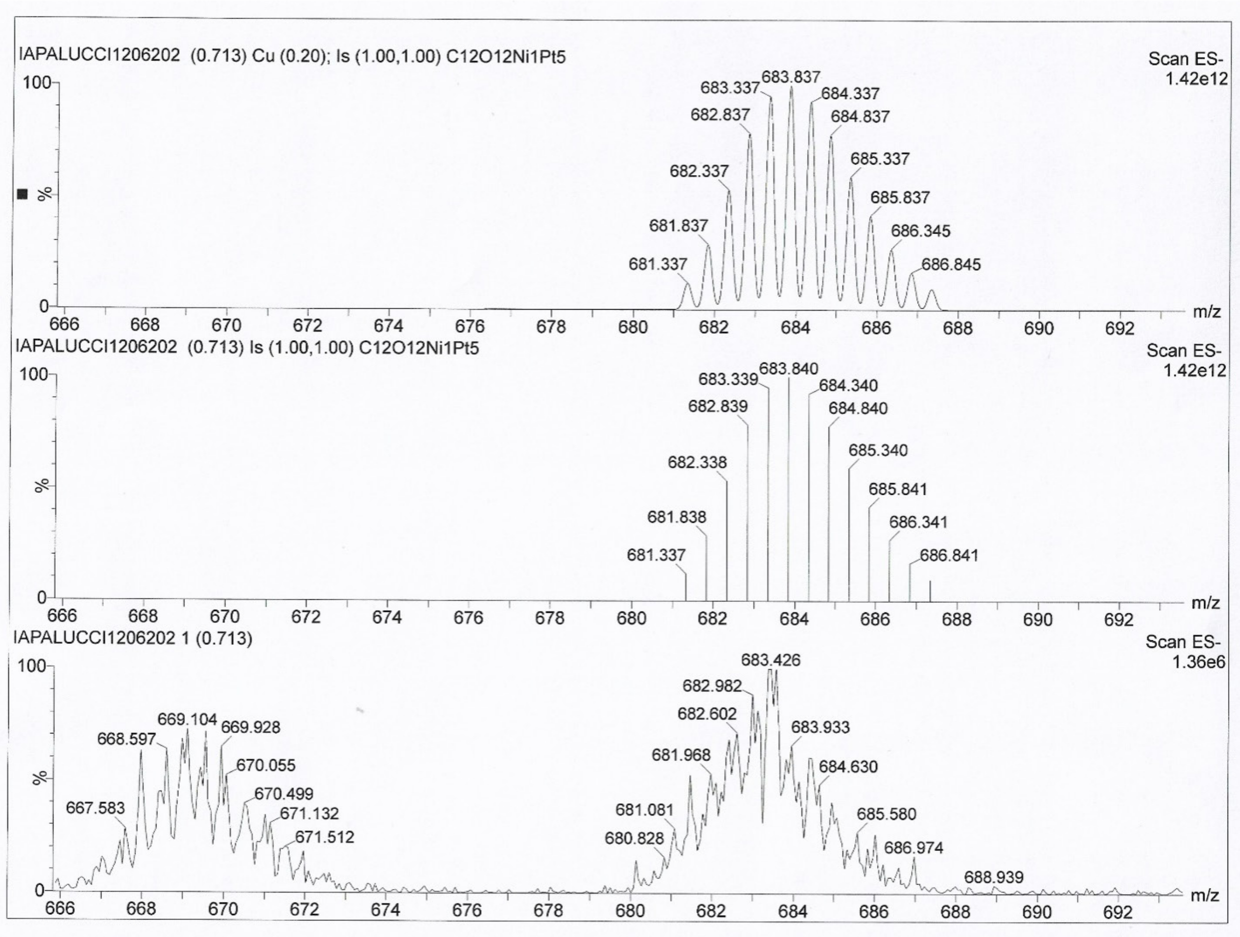

Figure S26

Isotopic pattern of the peak at m/z 698 of the ESI-MS spectrum in CH_3CN (ES–) of $[NBu_4]_2[Pt_9(CO)_{18}]$ + 1.2$[NBu_4]_2[Ni_6(CO)_{12}]$ after work-up. Upper traces: calculated isotopic patter for $[Pt_5Ni(CO)_{13}]^{2-}$. Lower trace: experimental isotopic pattern.

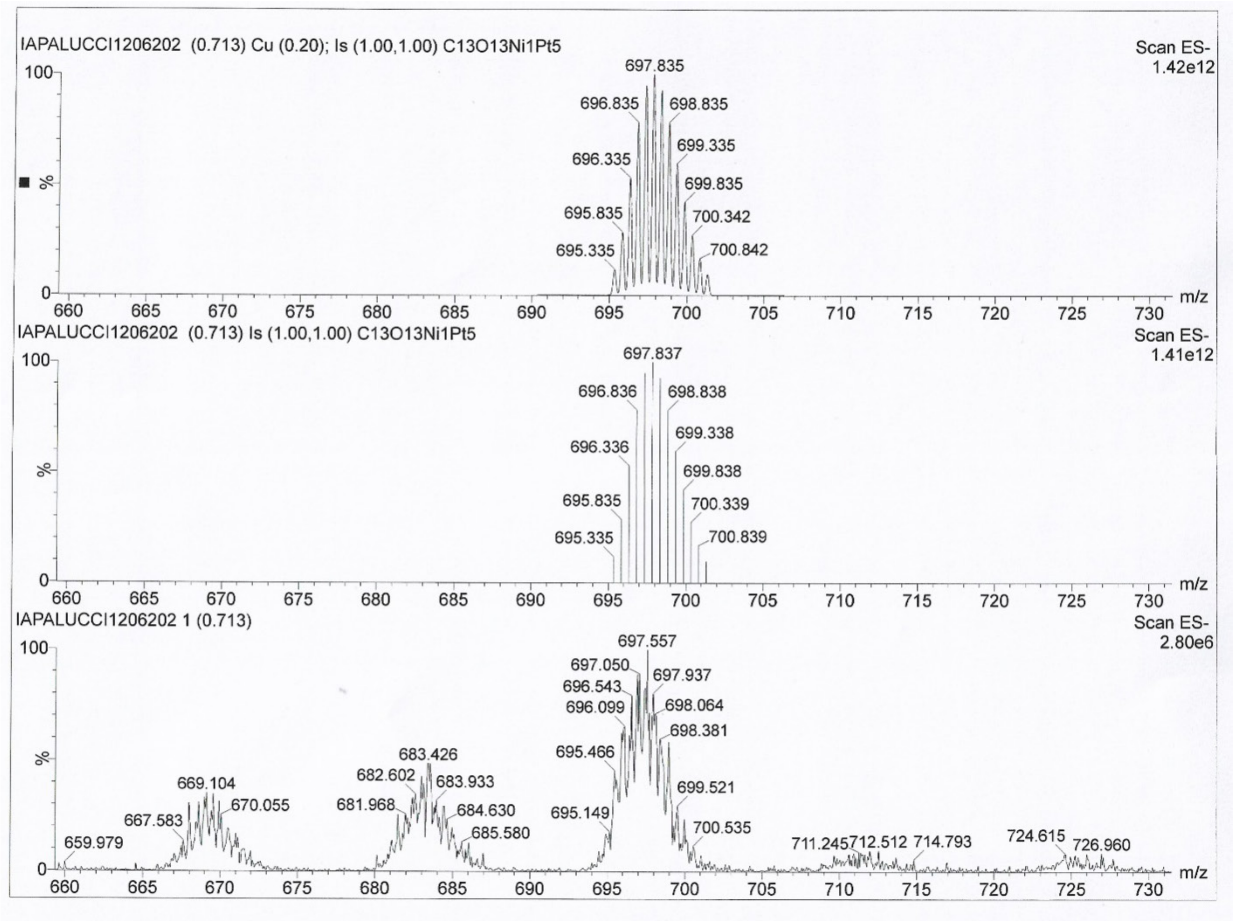

Figure S27

Isotopic pattern of the peak at m/z 1749 of the ESI-MS spectrum in CH_3CN (ES–) of $[NBu_4]_2[Pt_9(CO)_{18}]$ + 1.2$[NBu_4]_2[Ni_6(CO)_{12}]$ after work-up. Upper traces: calculated isotopic patter for $\{[Pt_6(CO)_{12}][NBu_4]\}^-$. Lower trace: experimental isotopic pattern.

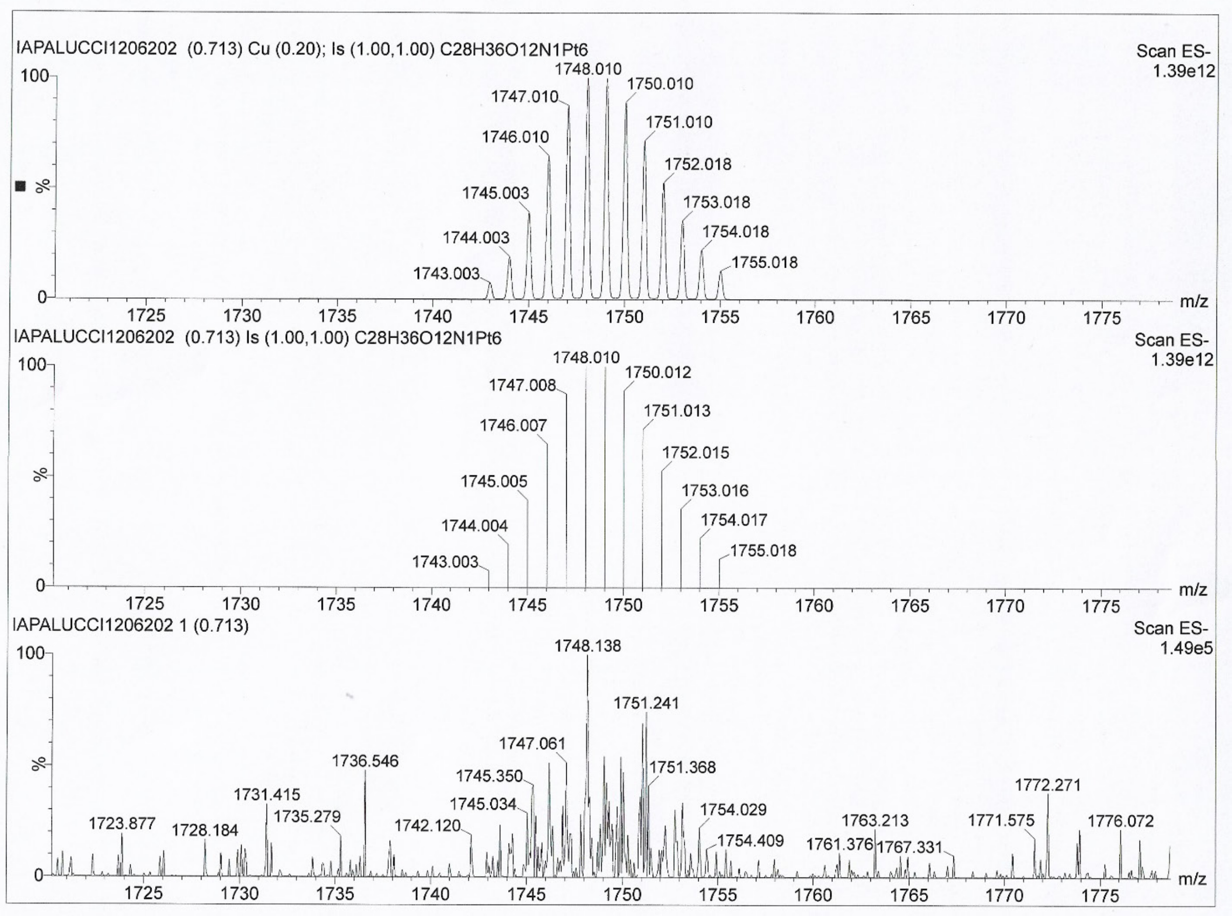

Figure S28

ESI-MS spectrum in CH_3CN (ES–) of $[NBu_4]_2[Pt_{6-x}Ni_x(CO)_{12}]$ (mixture of x = 3.24, 4.15, 4.16).

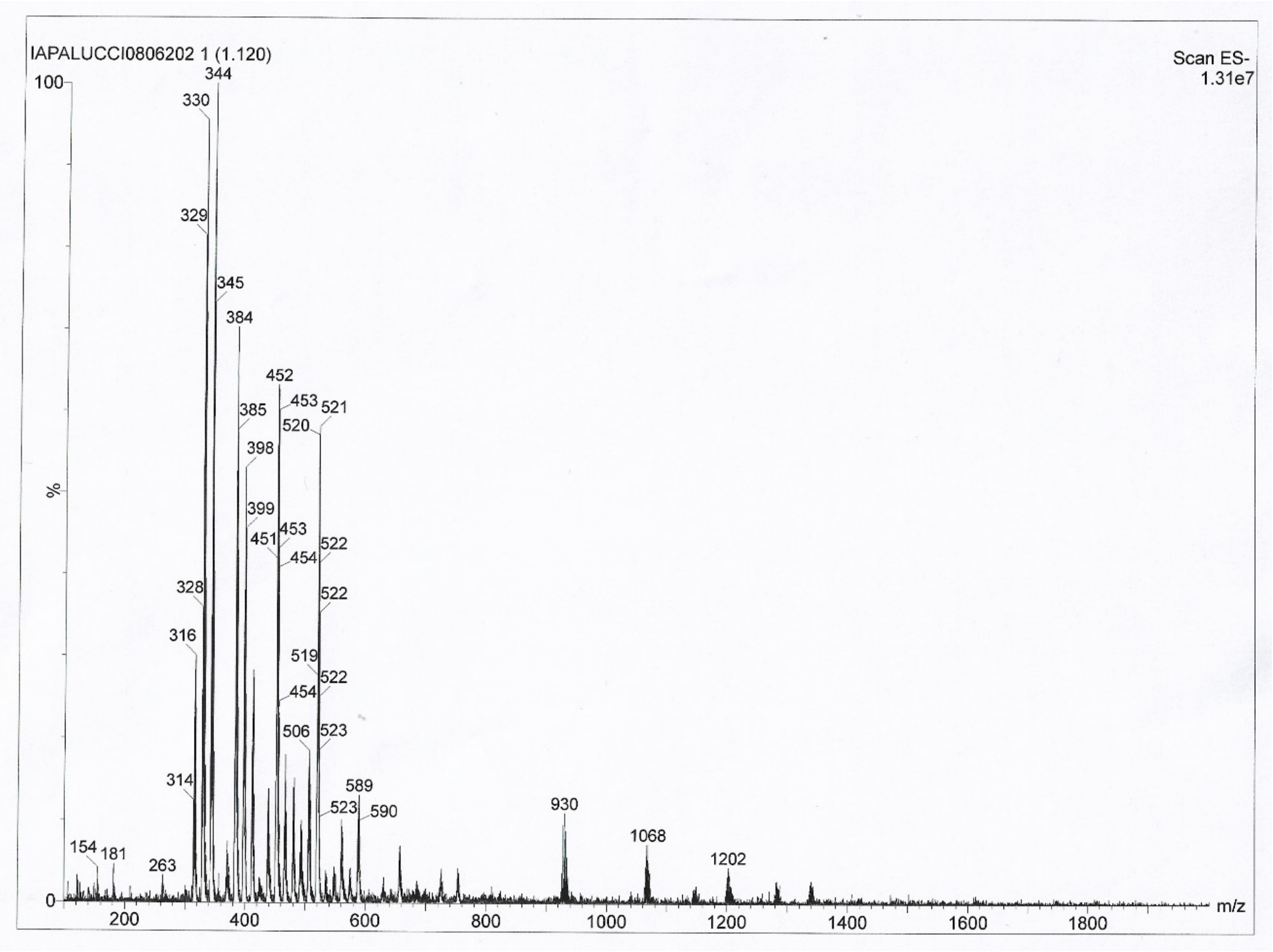

Table S3

Peak assignment of the ESI-MS spectrum (ES–) of $[NBu_4]_2[Pt_{6-x}Ni_x(CO)_{12}]$ (mixture of x = 3.24, 4.15, 4.16).

m/z	Relative intensity	Ion	Code	Sum of the peaks intensities
1338	2	$\{[Pt_3Ni_3(CO)_{12}][NBut_4]\}^-$	Pt_3Ni_3+NBu_4	-
1202	5	$\{[Pt_2Ni_4(CO)_{12}][NBut_4]\}^-$	Pt_2Ni_4+NBu_4	-
1068	8	$\{[PtNi_5(CO)_{12}][NBut_4]\}^-$	$PtNi_5$+NBu_4	-
930	10	$\{[Ni_6(CO)_{12}][NBut_4]\}^-$	Ni_6+NBu_4	-
753	5	$[Pt_6(CO)_{12}]^{2-}$	Pt_6	5
657	8	$[Pt_5Ni(CO)_{10}]^{2-}$	Pt_5Ni–2CO	
589	14	$[Pt_4Ni_2(CO)_{10}]^{2-}$	Pt_4Ni_2–2CO	14
548	10	$[Pt_3Ni_3(CO)_{12}]^{2-}$	Pt_3Ni_3	97
535	5	$[Pt_3Ni_3(CO)_{11}]^{2-}$	Pt_3Ni_3–1CO	
521	60	$[Pt_3Ni_3(CO)_{10}]^{2-}$	Pt_3Ni_3–2CO	
506	20	$[Pt_3Ni_3(CO)_9]^{2-}$	Pt_3Ni_3–3CO	
480	55	$[Pt_2Ni_4(CO)_{12}]^{2-}$	Pt_2Ni_4	130
466	10	$[Pt_2Ni_4(CO)_{11}]^{2-}$	Pt_2Ni_4–1CO	
452	60	$[Pt_2Ni_4(CO)_{10}]^{2-}$	Pt_2Ni_4–2CO	
412	30	$[PtNi_5(CO)_{12}]^{2-}$	$PtNi_5$	158
398	50	$[PtNi_5(CO)_{11}]^{2-}$	$PtNi_5$–1CO	
384	70	$[PtNi_5(CO)_{10}]^{2-}$	$PtNi_5$–2CO	
344	100	$[Ni_6(CO)_{12}]^{2-}$	Ni_6	235
330	95	$[Ni_6(CO)_{11}]^{2-}$	Ni_6–1CO	
316	30	$[Ni_6(CO)_{10}]^{2-}$	Ni_6–1CO	

Figure S29

Isotopic pattern of the peak at m/z 344 of the ESI-MS spectrum in CH_3CN (ES–) of $[NBu_4]_2[Pt_{6-x}Ni_x(CO)_{12}]$ (mixture of x = 3.24, 4.15, 4.16). Upper traces: calculated isotopic patter for $[Ni_6(CO)_{12}]^{2-}$. Lower trace: experimental isotopic pattern.

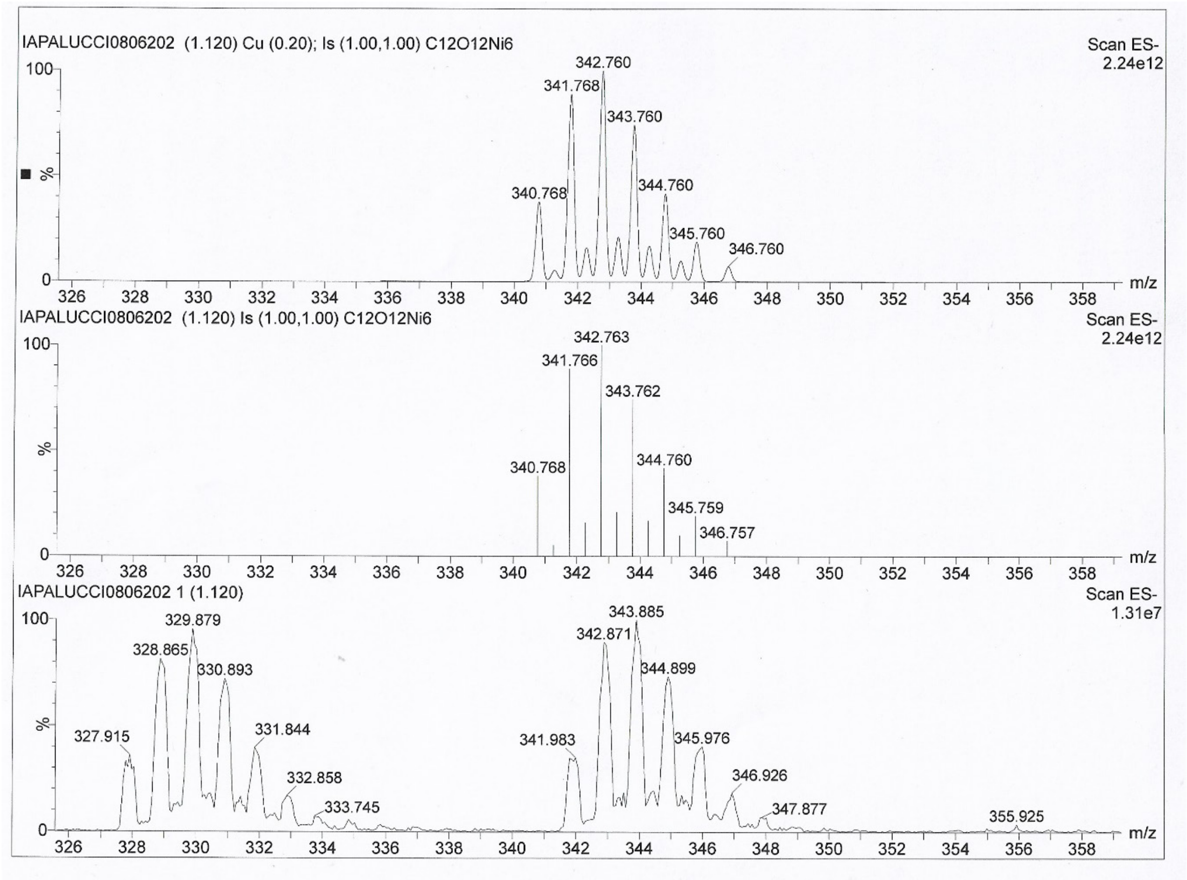

Figure S30

Isotopic pattern of the peak at m/z 412 of the ESI-MS spectrum in CH_3CN (ES–) of $[NBu_4]_2[Pt_{6-x}Ni_x(CO)_{12}]$ (mixture of x = 3.24, 4.15, 4.16). Upper traces: calculated isotopic patter for $[PtNi_5(CO)_{12}]^{2-}$. Lower trace: experimental isotopic pattern.

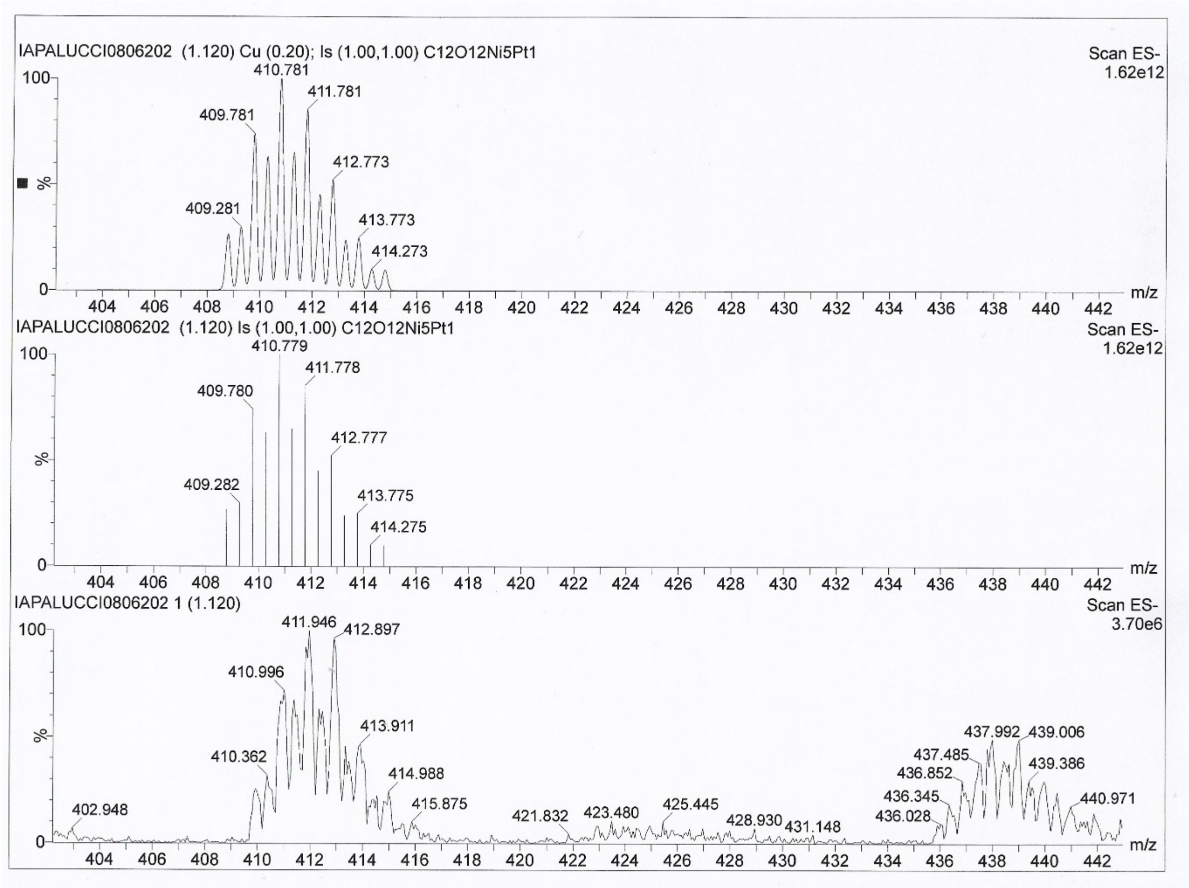

Figure S31

Isotopic pattern of the peak at m/z 480 of the ESI-MS spectrum in CH_3CN (ES–) of $[NBu_4]_2[Pt_{6-x}Ni_x(CO)_{12}]$ (mixture of x = 3.24, 4.15, 4.16). Upper traces: calculated isotopic patter for $[Pt_2Ni_4(CO)_{12}]^{2-}$. Lower trace: experimental isotopic pattern.

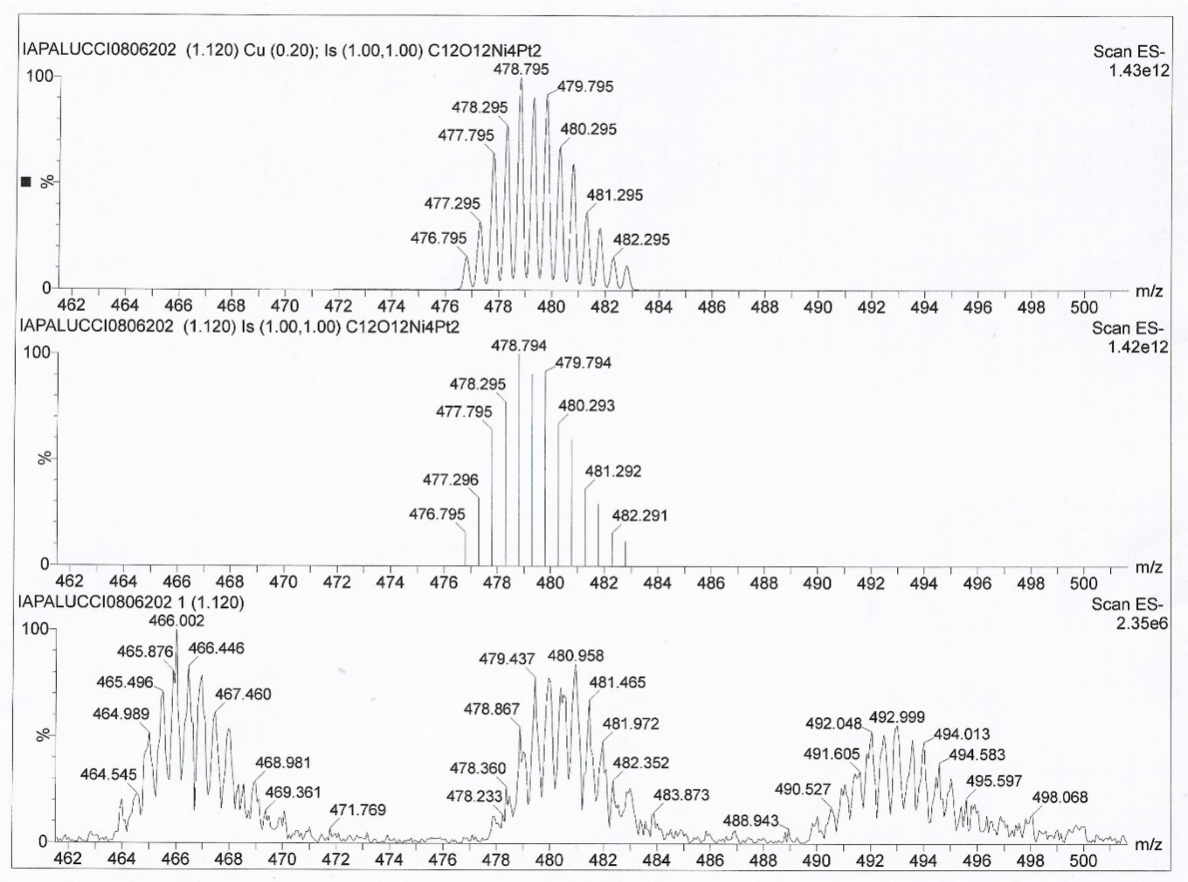

Figure S32

Isotopic pattern of the peak at m/z 521 of the ESI-MS spectrum in CH_3CN (ES–) of $[NBu_4]_2[Pt_{6-x}Ni_x(CO)_{12}]$ (mixture of x = 3.24, 4.15, 4.16). Upper traces: calculated isotopic patter for $[Pt_3Ni_3(CO)_{10}]^{2-}$. Lower trace: experimental isotopic pattern.

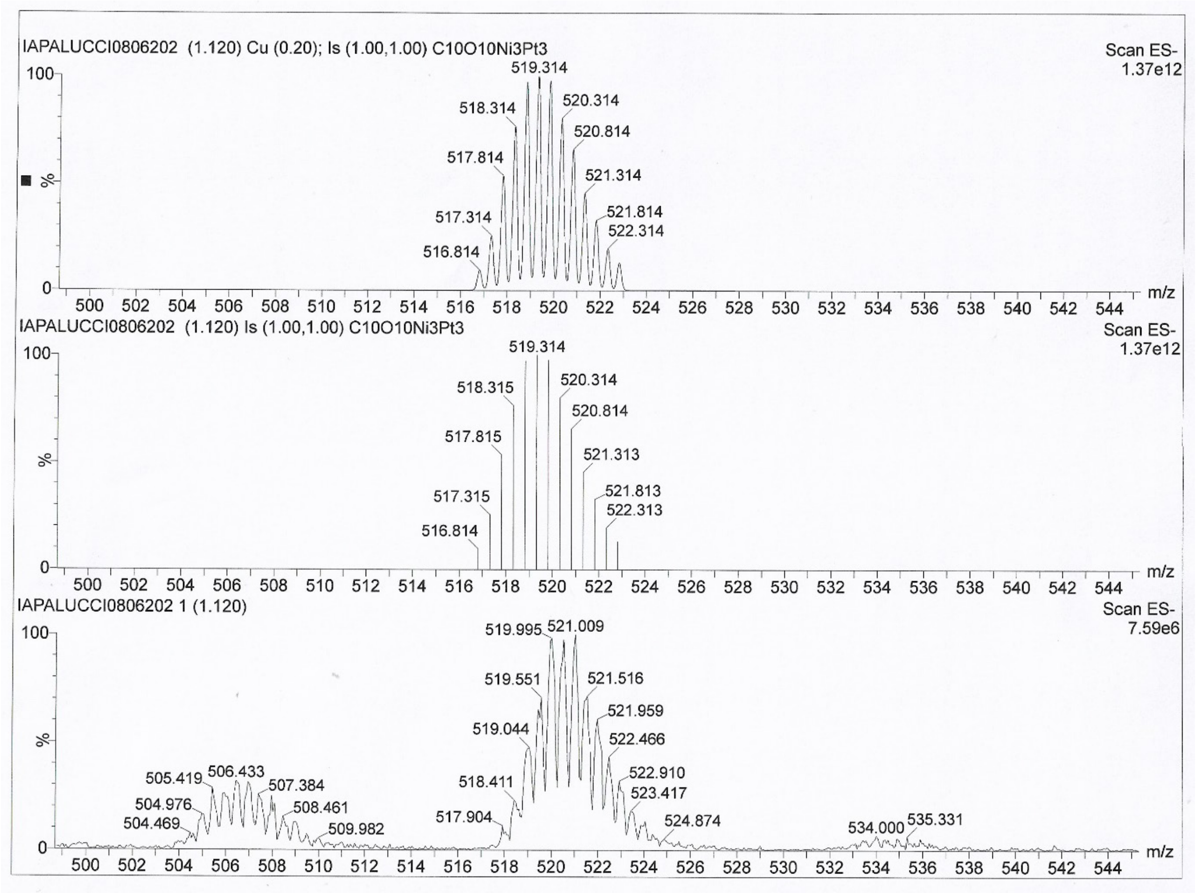

Figure S33

Isotopic pattern of the peak at m/z 930 of the ESI-MS spectrum in CH_3CN (ES–) of $[NBu_4]_2[Pt_{6-x}Ni_x(CO)_{12}]$ (mixture of x = 3.24, 4.15, 4.16). Upper traces: calculated isotopic patter for $\{[Ni_6(CO)_{12}][NBu_4]\}^-$. Lower trace: experimental isotopic pattern.

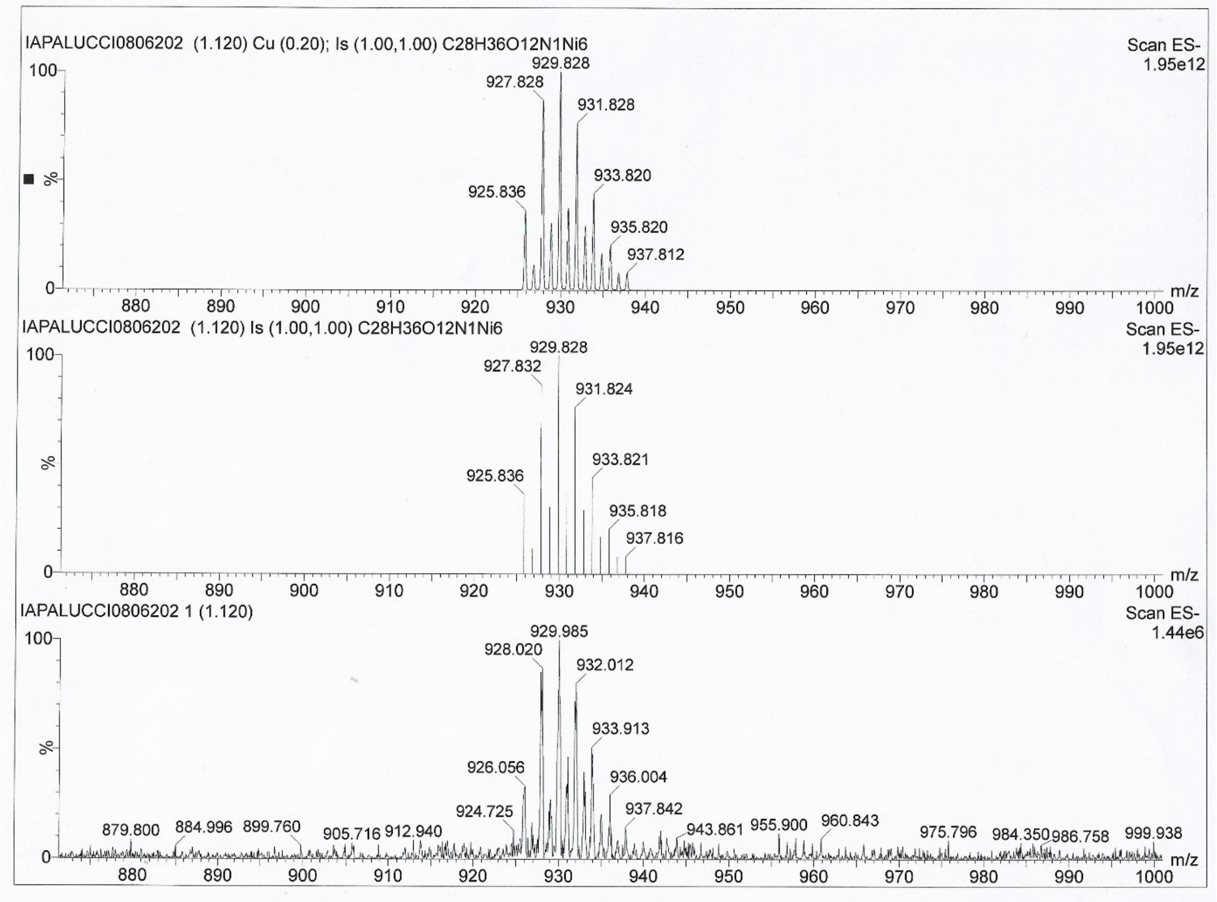

Figure S34

Isotopic pattern of the peak at m/z 1068 of the ESI-MS spectrum in CH_3CN (ES–) of $[NBu_4]_2[Pt_{6-x}Ni_x(CO)_{12}]$ (mixture of x = 3.24, 4.15, 4.16). Upper traces: calculated isotopic patter for $\{[PtNi_5(CO)_{12}][NBu_4]\}^-$. Lower trace: experimental isotopic pattern.

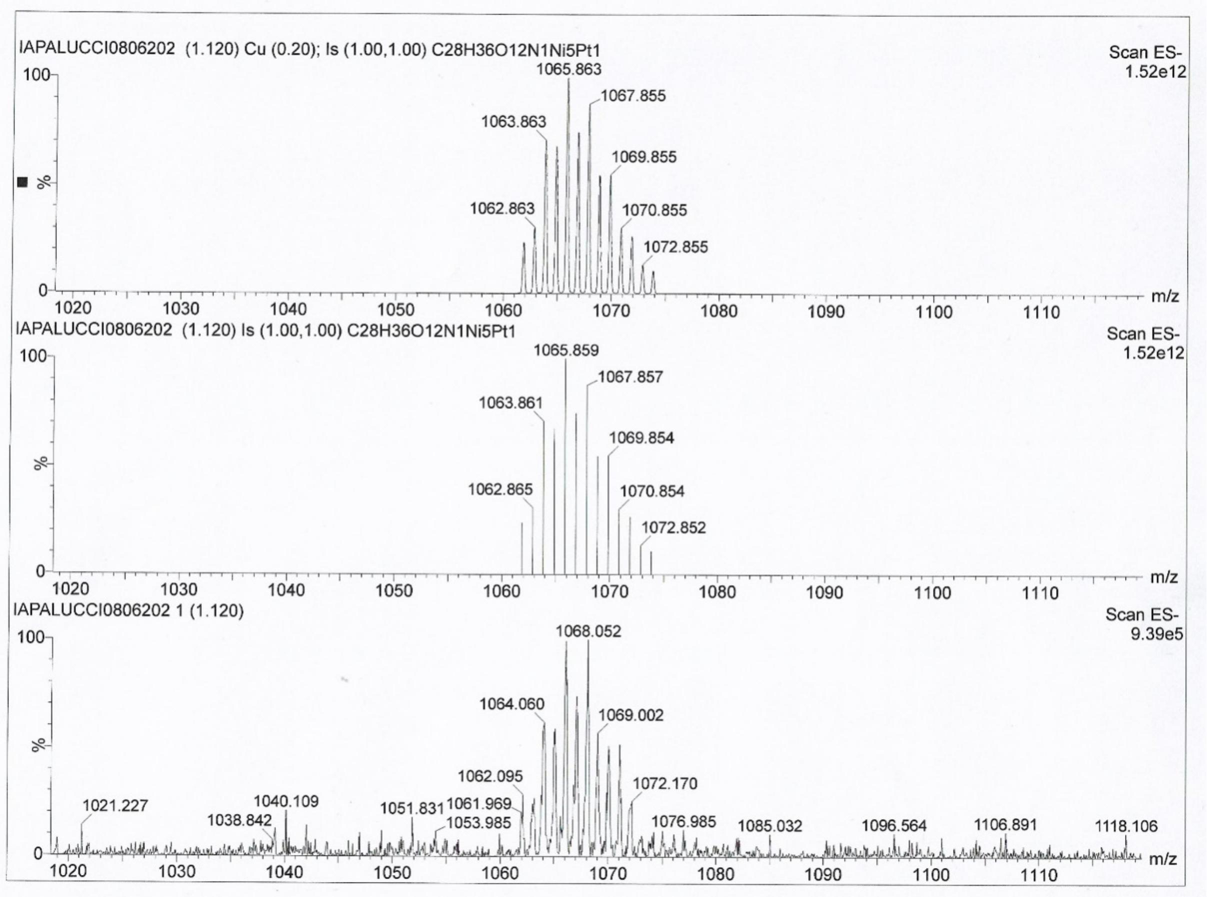

Figure S35

Isotopic pattern of the peak at m/z 1202 of the ESI-MS spectrum in CH_3CN (ES–) of $[NBu_4]_2[Pt_{6-x}Ni_x(CO)_{12}]$ (mixture of x = 3.24, 4.15, 4.16). Upper traces: calculated isotopic patter for $\{[Pt_2Ni_4(CO)_{12}][NBu_4]\}^-$. Lower trace: experimental isotopic pattern.

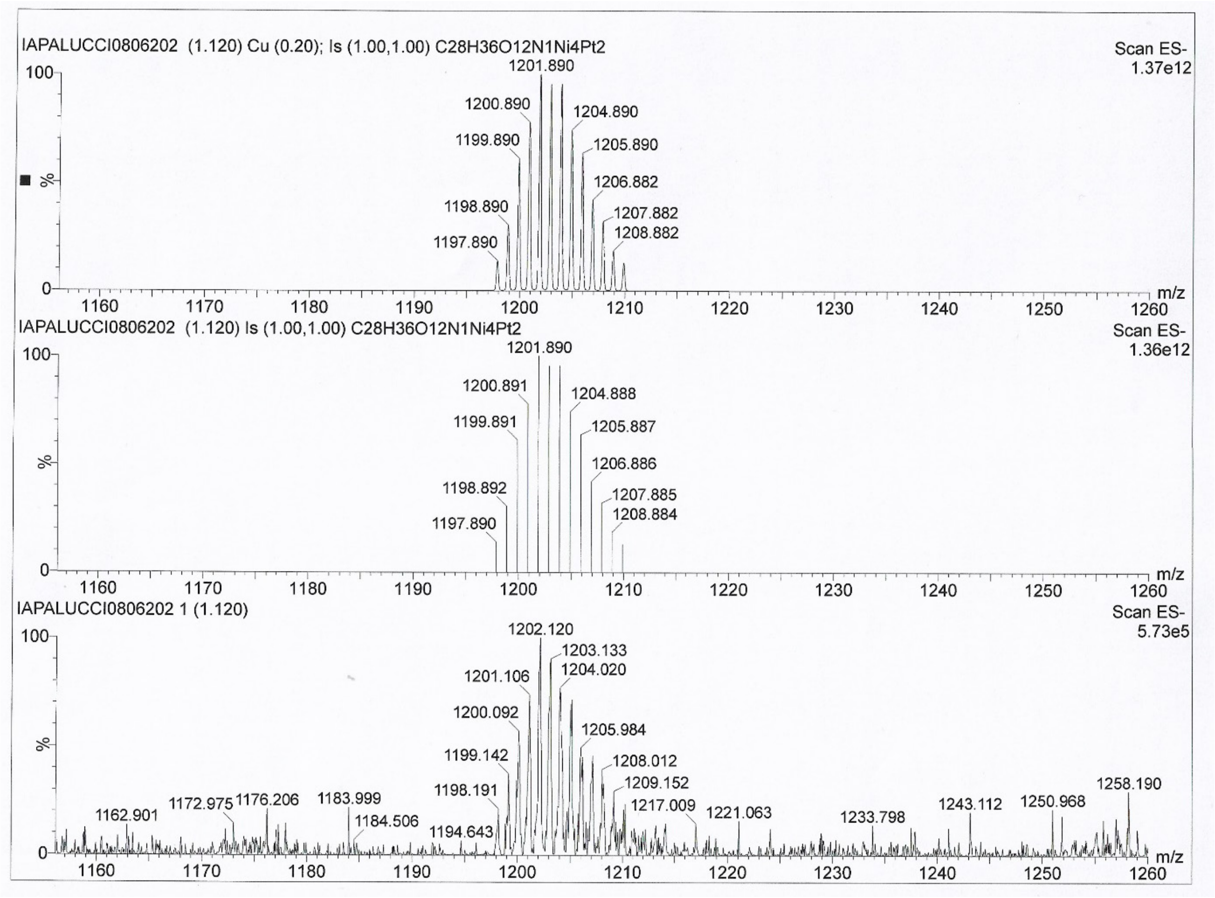

Figure S36

Isotopic pattern of the peak at m/z 1338 of the ESI-MS spectrum in CH_3CN (ES–) of $[NBu_4]_2[Pt_{6-x}Ni_x(CO)_{12}]$ (mixture of x = 3.24, 4.15, 4.16). Upper traces: calculated isotopic patter for $\{[Pt_3Ni_3(CO)_{12}][NBu_4]\}^-$. Lower trace: experimental isotopic pattern.

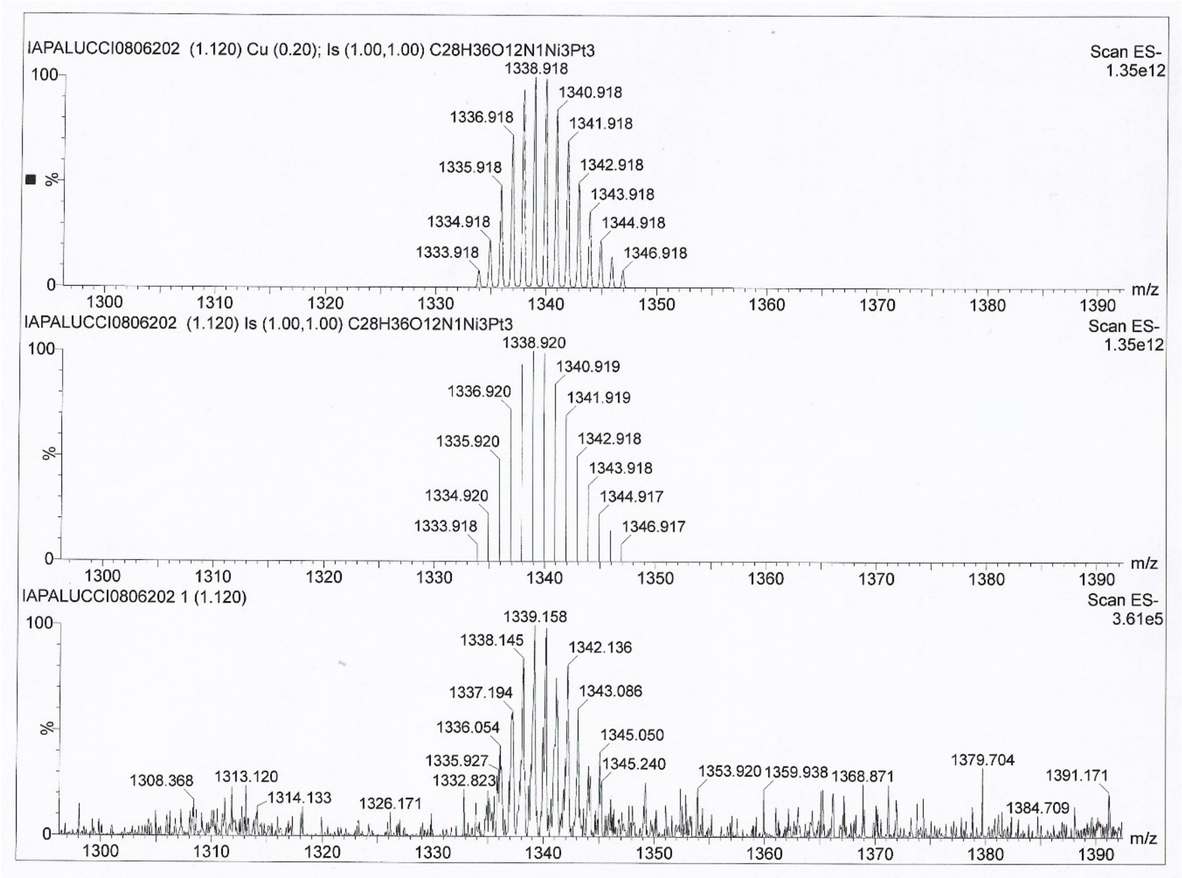

Figure S37

ESI-MS spectrum in CH_3CN (ES–) of $[NBu_4]_2[Pt_6(CO)_{11}]$ + 1.0$[NBu_4]_2[Ni_6(CO)_{12}]$ after work-up.

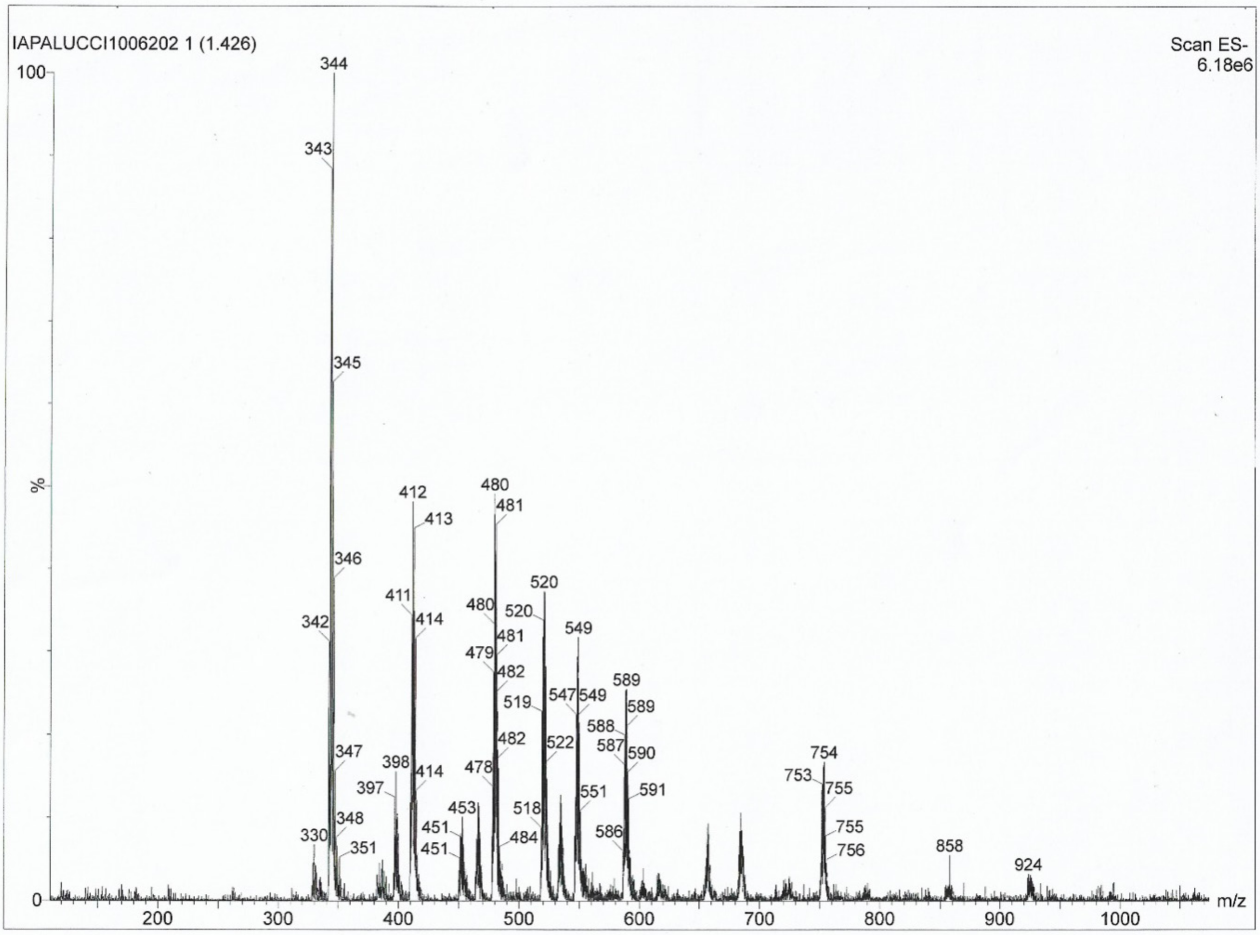

Table S4

Peak assignment of the ESI-MS spectrum (ES–) of $[NBu_4]_2[Pt_6(CO)_{11}]$ + 1.0$[NBu_4]_2[Ni_6(CO)_{12}]$ after work-up.

m/z	Relative intensity	Ion	Code	Sum of the peaks intensities
753	18	$[Pt_6(CO)_{12}]^{2-}$	Pt_6	18
684	10	$[Pt_5Ni(CO)_{12}]^{2-}$	Pt_5Ni	20
658	10	$[Pt_5Ni(CO)_{10}]^{2-}$	Pt_5Ni–2CO	
616	4	$[Pt_4Ni_2(CO)_{12}]^{2-}$	Pt_4Ni_2	
603	4	$[Pt_4Ni_2(CO)_{11}]^{2-}$	Pt_4Ni_2–1CO	33
588	25	$[Pt_4Ni_2(CO)_{10}]^{2-}$	Pt_4Ni_2–2CO	
548	30	$[Pt_3Ni_3(CO)_{12}]^{2-}$	Pt_3Ni_3	
534	15	$[Pt_3Ni_3(CO)_{11}]^{2-}$	Pt_3Ni_3–1CO	85
520	40	$[Pt_3Ni_3(CO)_{10}]^{2-}$	Pt_3Ni_3–2CO	
480	50	$[Pt_2Ni_4(CO)_{12}]^{2-}$	Pt_2Ni_4	
466	12	$[Pt_2Ni_4(CO)_{11}]^{2-}$	Pt_2Ni_4–1CO	72
453	10	$[Pt_2Ni_4(CO)_{10}]^{2-}$	Pt_2Ni_4–2CO	
412	50	$[PtNi_5(CO)_{12}]^{2-}$	$PtNi_5$	
398	15	$[PtNi_5(CO)_{11}]^{2-}$	$PtNi_5$–1CO	70
385	5	$[PtNi_5(CO)_{10}]^{2-}$	$PtNi_5$–2CO	
344	100	$[Ni_6(CO)_{12}]^{2-}$	Ni_6	108
330	8	$[Ni_6(CO)_{11}]^{2-}$	Ni_6–1CO	

Figure S38

Isotopic pattern of the peak at m/z 344 of the ESI-MS spectrum in CH_3CN (ES–) of $[NBu_4]_2[Pt_6(CO)_{11}]$ + 1.0$[NBu_4]_2[Ni_6(CO)_{12}]$ after work-up. Upper traces: calculated isotopic patter for $[Ni_6(CO)_{12}]^{2-}$. Lower trace: experimental isotopic pattern.

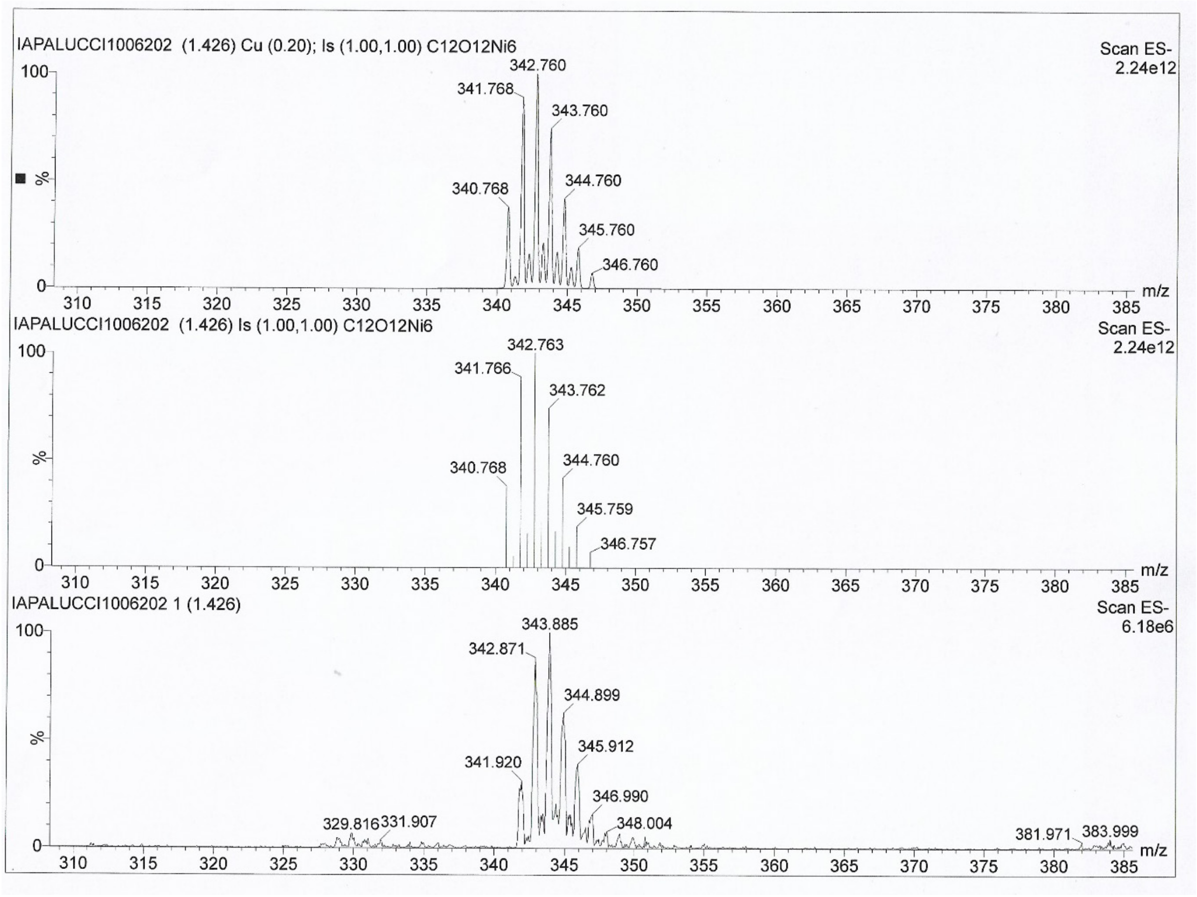

Figure S39

Isotopic pattern of the peak at m/z 412 of the ESI-MS spectrum in CH_3CN (ES–) of $[NBu_4]_2[Pt_6(CO)_{11}]$ + 1.0$[NBu_4]_2[Ni_6(CO)_{12}]$ after work-up. Upper traces: calculated isotopic patter for $[PtNi_5(CO)_{12}]^{2-}$. Lower trace: experimental isotopic pattern.

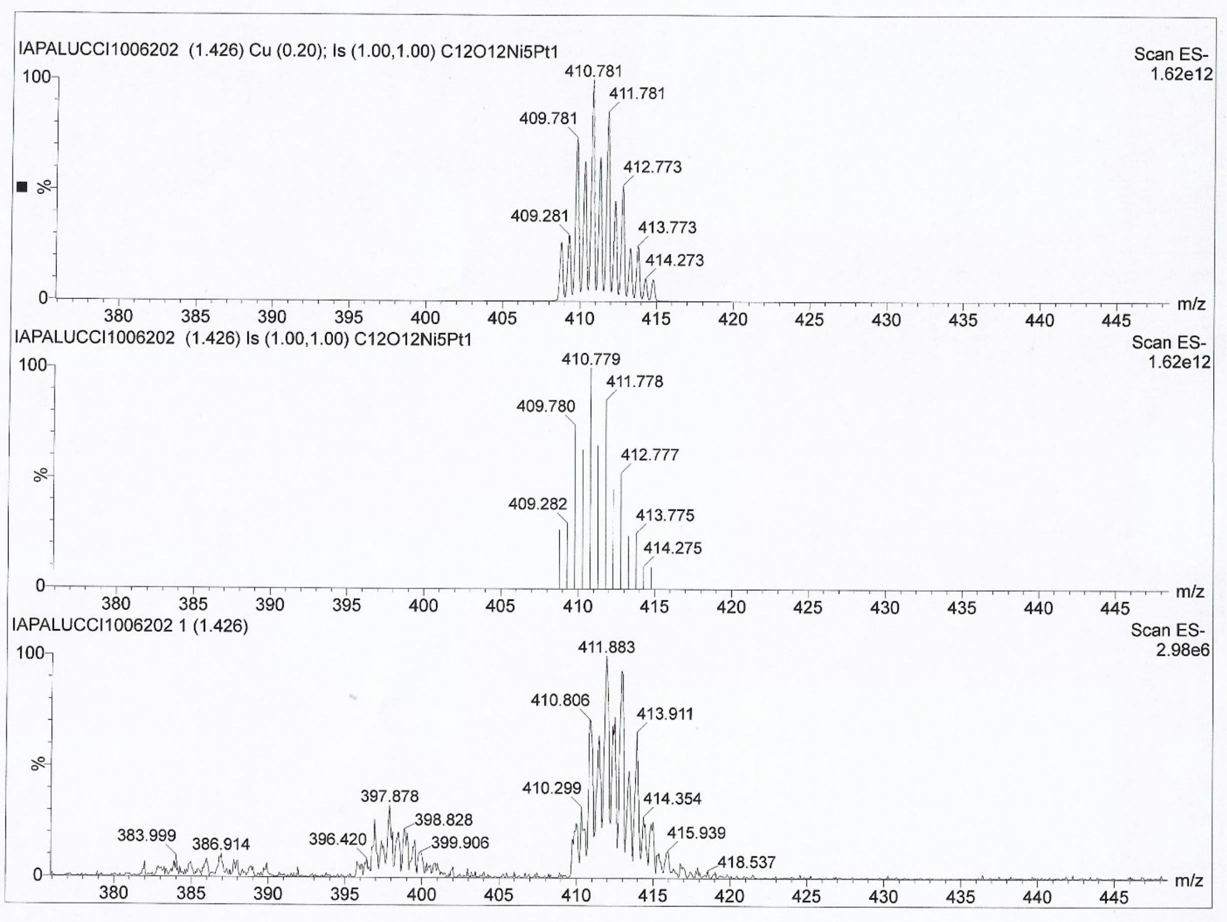

Figure S40

Isotopic pattern of the peak at m/z 480 of the ESI-MS spectrum in CH_3CN (ES–) of $[NBu_4]_2[Pt_6(CO)_{11}]$ + 1.0$[NBu_4]_2[Ni_6(CO)_{12}]$ after work-up. Upper traces: calculated isotopic patter for $[Pt_2Ni_4(CO)_{12}]^{2-}$. Lower trace: experimental isotopic pattern.

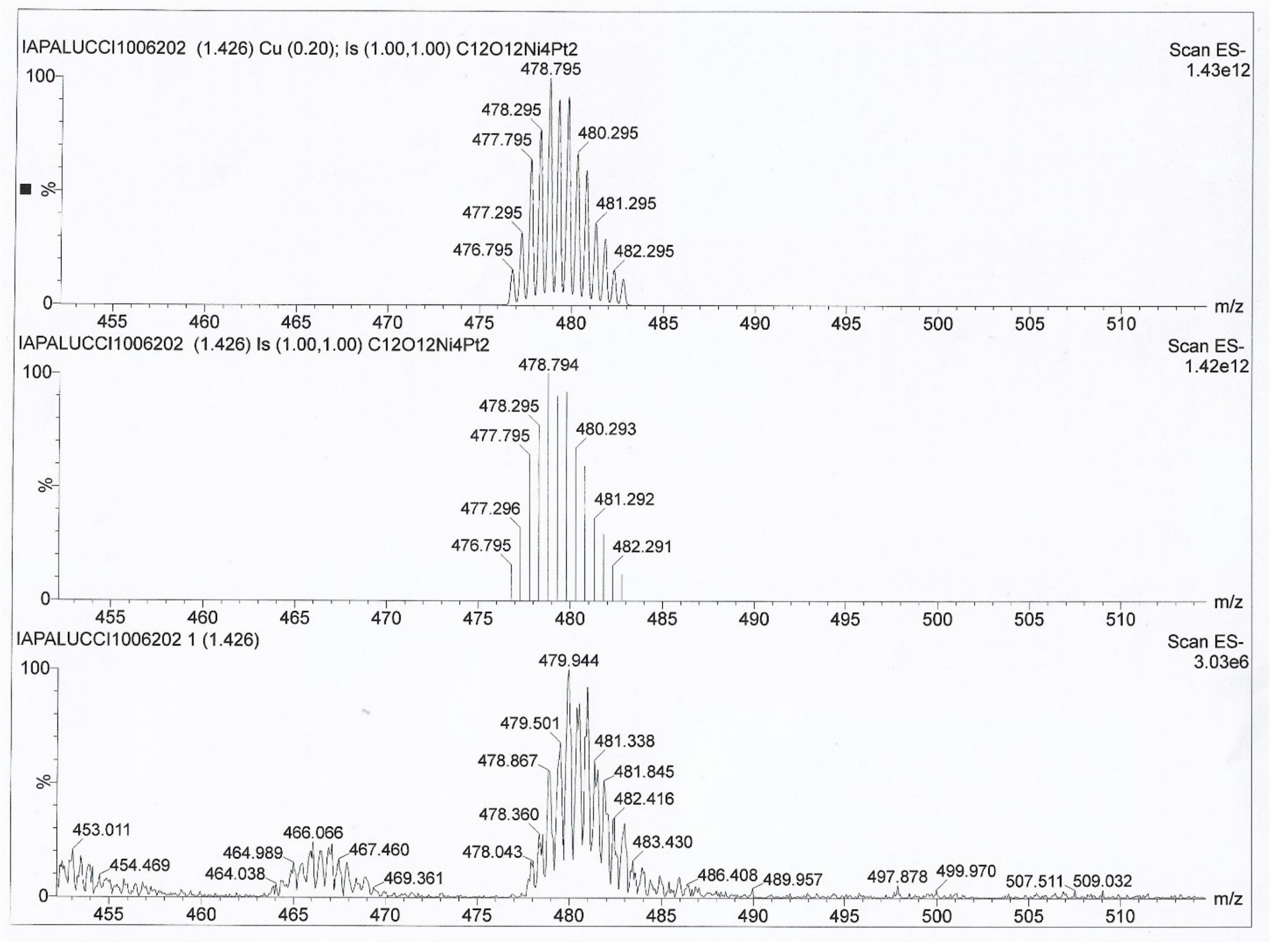

Figure S41

Isotopic pattern of the peak at m/z 520 of the ESI-MS spectrum in CH_3CN (ES–) of $[NBu_4]_2[Pt_6(CO)_{11}]$ + 1.0$[NBu_4]_2[Ni_6(CO)_{12}]$ after work-up. Upper traces: calculated isotopic patter for $[Pt_3Ni_3(CO)_{10}]^{2-}$. Lower trace: experimental isotopic pattern.

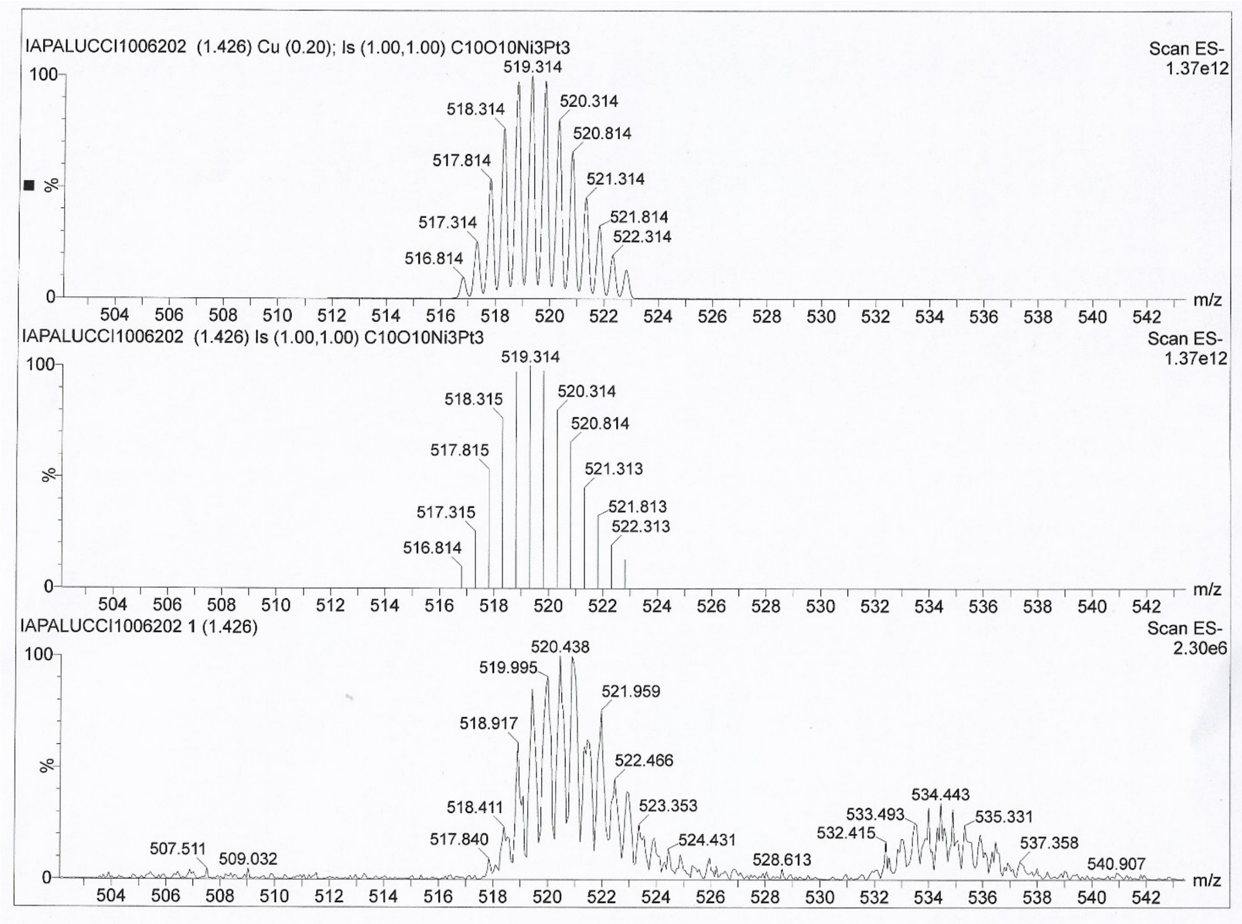

Figure S42

Isotopic pattern of the peak at m/z 588 of the ESI-MS spectrum in CH_3CN (ES–) of $[NBu_4]_2[Pt_6(CO)_{11}]$ + 1.0$[NBu_4]_2[Ni_6(CO)_{12}]$ after work-up. Upper traces: calculated isotopic patter for $[Pt_4Ni_2(CO)_{10}]^{2-}$. Lower trace: experimental isotopic pattern.

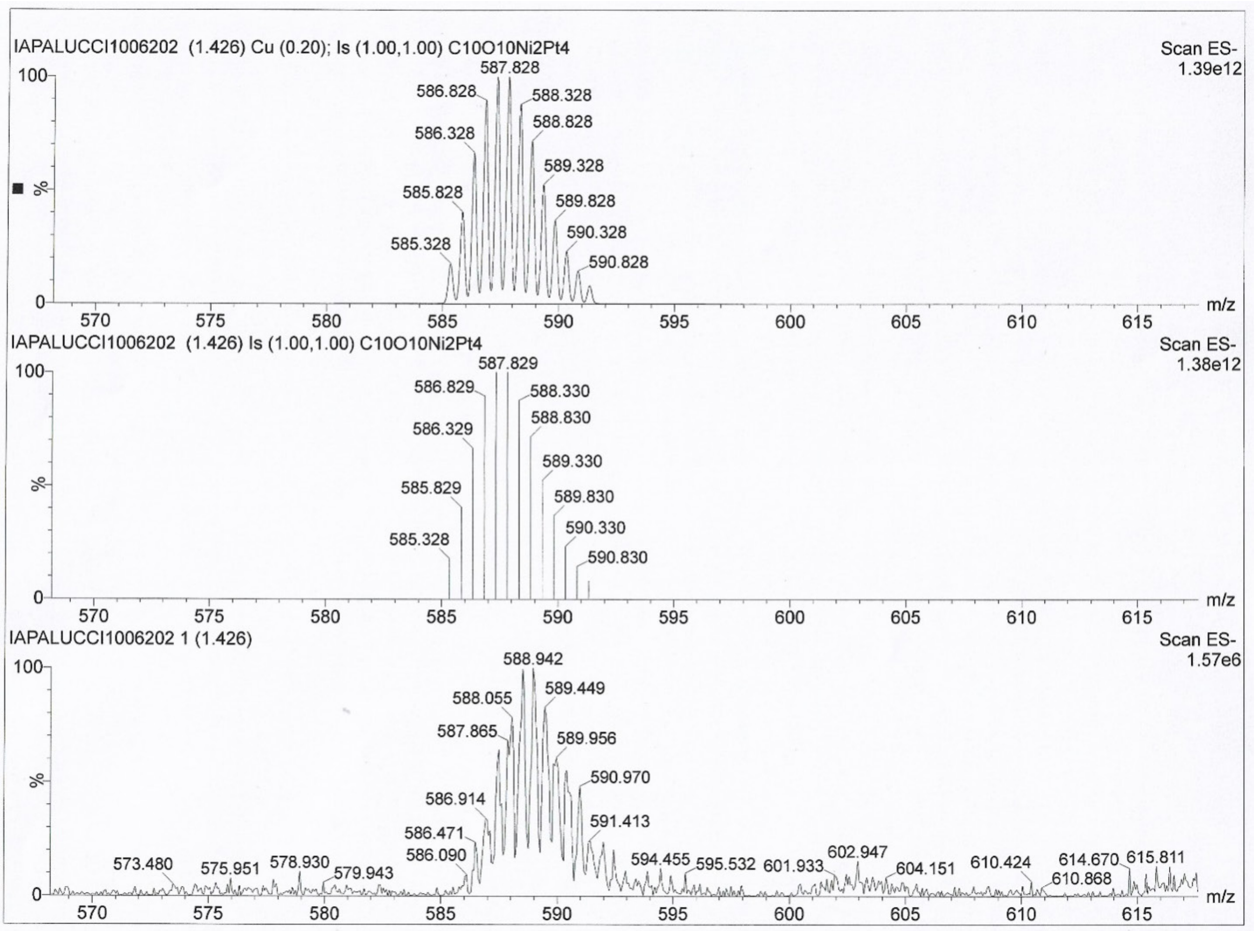

Figure S43

Isotopic pattern of the peak at m/z 754 of the ESI-MS spectrum in CH_3CN (ES–) of $[NBu_4]_2[Pt_6(CO)_{11}]$ + 1.0$[NBu_4]_2[Ni_6(CO)_{12}]$ after work-up. Upper traces: calculated isotopic patter for $[Pt_6(CO)_{12}]^{2-}$. Lower trace: experimental isotopic pattern.

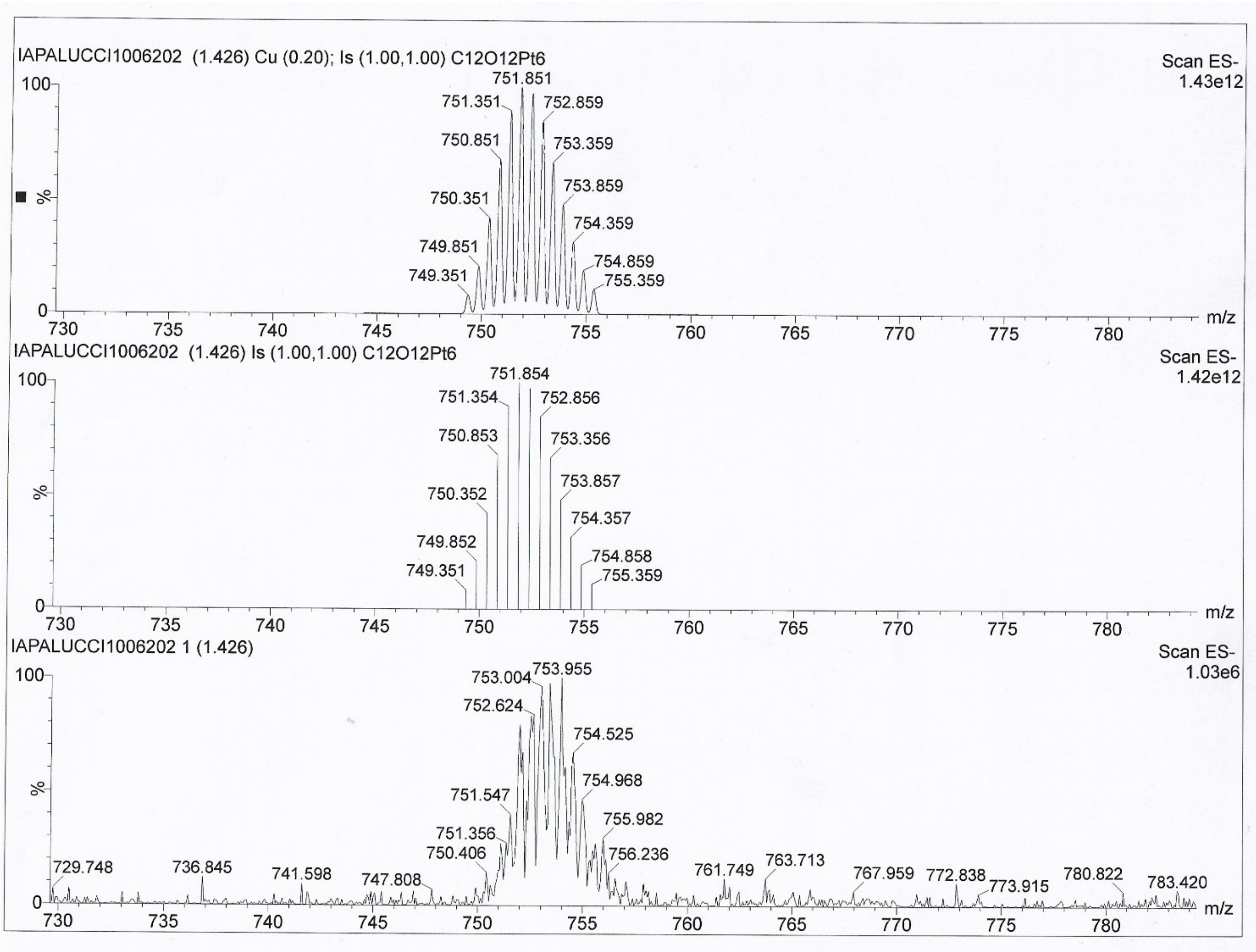

Figure S44

^{195}Pt NMR spectra of $[NBu_4]_2[Pt_6(CO)_{12}]$ + $2[NBu_4]_2[Ni_6(CO)_{12}]$ in CD_3COCD_3 at 298 K recorded at different times.

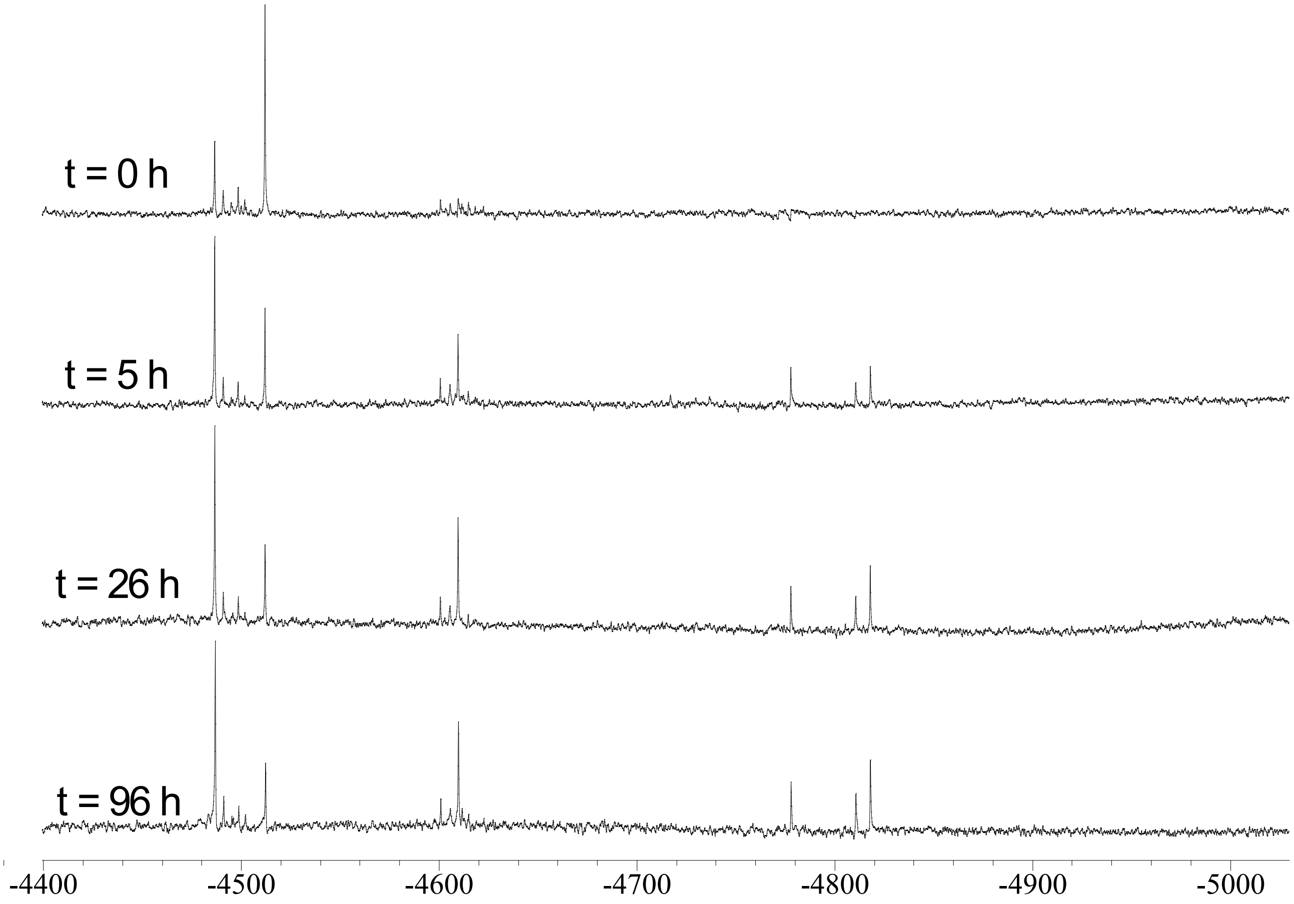

Figure S45

^{195}Pt NMR spectra in CD_3COCD_3 at 298 K of (a) $[NBu_4]_2[Pt_6(CO)_{12}]$, (b) $[NBu_4]_2[Pt_{6-x}Ni_x(CO)_{12}]$ (x = 1.25), (c) $[NBu_4]_4[Pt_{6-x}Ni_x(CO)_{12}][Cl_{1.77}Br_{0.23}]$ (x = 2.53), (d) $[NMe_4]_2[Pt_6(CO)_{12}]$ + $[NMe_4]_2[Ni_6(CO)_{12}]$ (1:1), (e) $[NBu_4]_2[Pt_{6-x}Ni_x(CO)_{12}]$ (mixture of x = 3.24, 4.15, 4.16), (f) $[NBu_4]_2[Pt_6(CO)_{12}]$ + $[NBu_4]_2[Ni_6(CO)_{12}]$ (1:5).

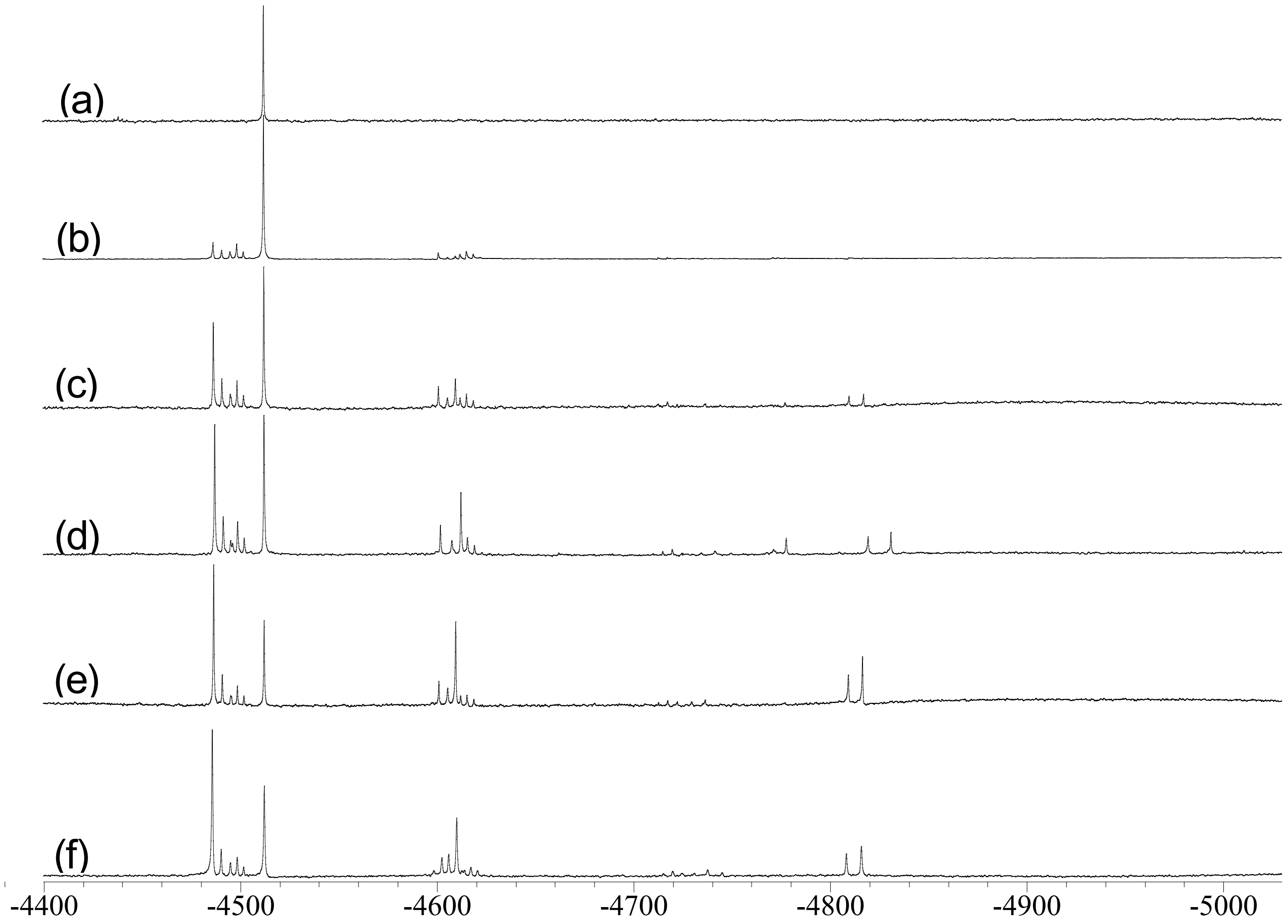

Figure S46

Comparison of (top) the simulated ^{195}Pt NMR spectrum of $[Pt_5Ni(CO)_{12}]^{2-}$ (δ_{Pt} -4497 (3Pt) and -4613 (2Pt), $^{1}J_{Pt-Pt}$ = 218 Hz) and (bottom) the experimental ^{195}Pt NMR spectrum of $[NBu_4]_2[Pt_{6-x}Ni_x(CO)_{12}]$ (x = 1.25) in CD_3COCD_3 at 298 K. The singlets at δ_{Pt} -4510 and -4613 ppm are due to $[Pt_6(CO)_{12}]^{2-}$ and $[Pt_3Ni_3(CO)_{12}]^{2-}$, respectively.

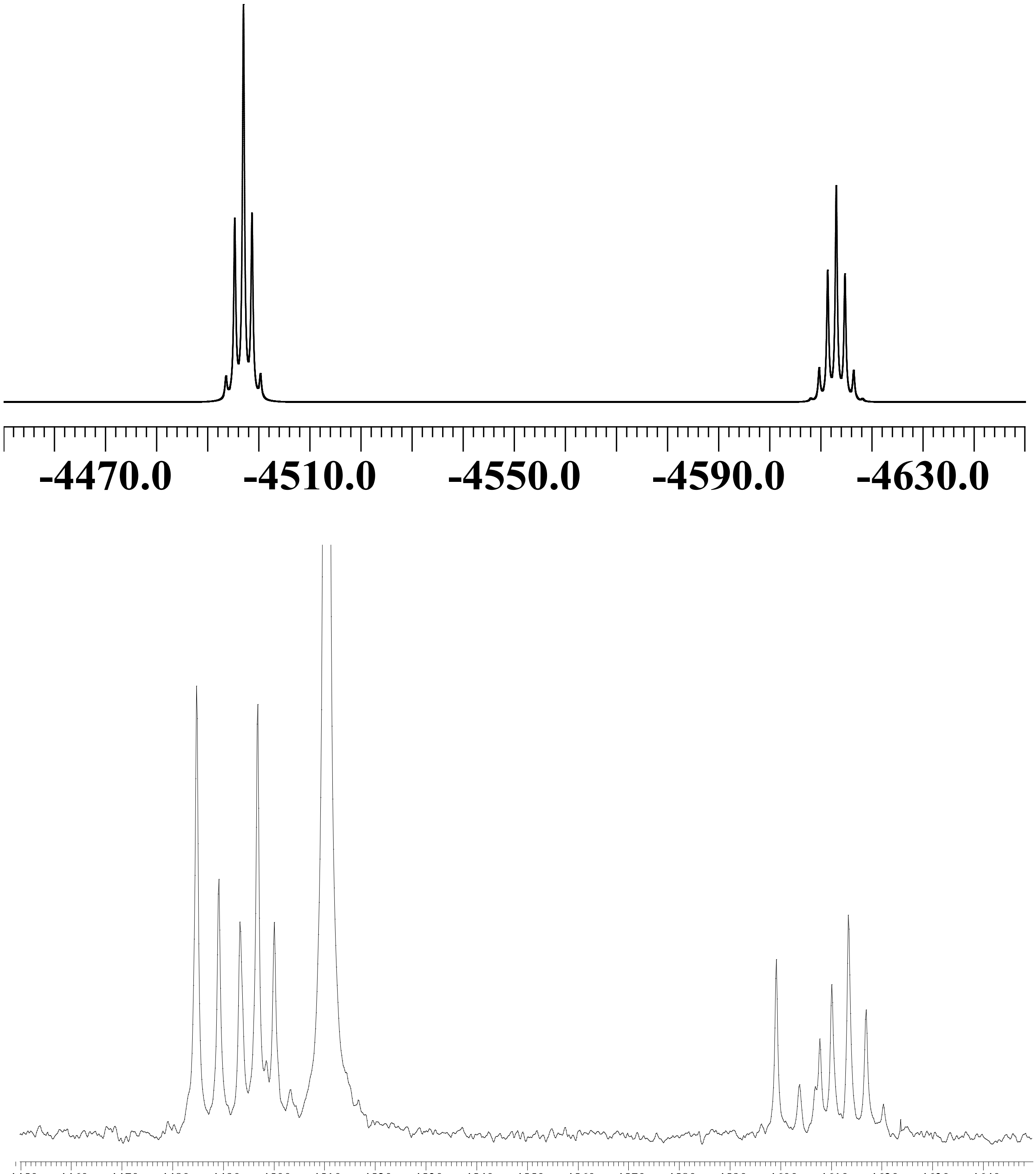

Figure S47

VT ^{195}Pt NMR spectra of $[NBu_4]_2[Pt_{\sim5}Ni_{\sim1}(CO)_{12}]$ in CD_3COCD_3.

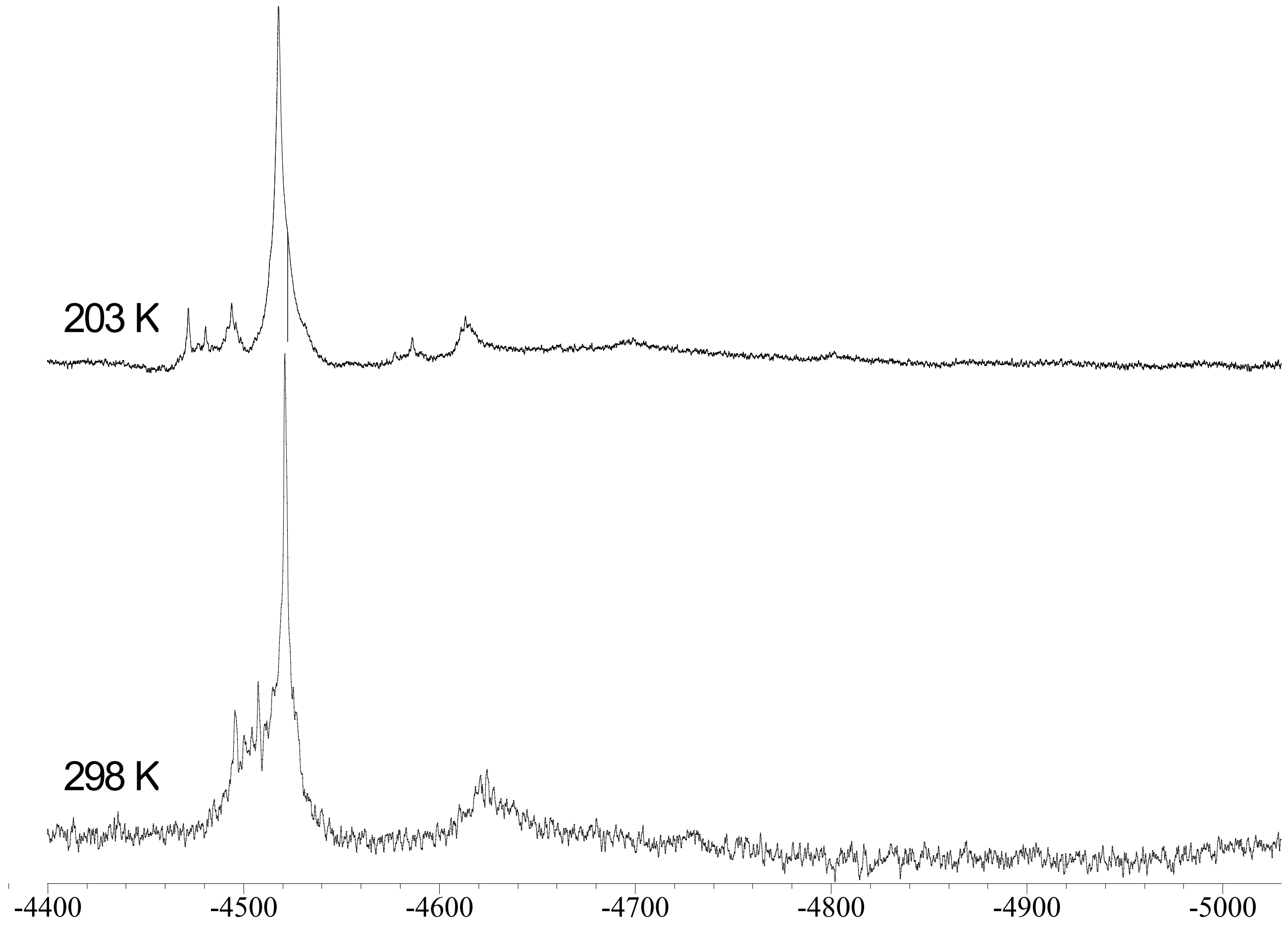

Figure S48

VT ^{13}C NMR spectra of $[NBu_4]_2[Pt_{\sim 5}Ni_{\sim 1}(CO)_{12}]$ in CD_3COCD_3.

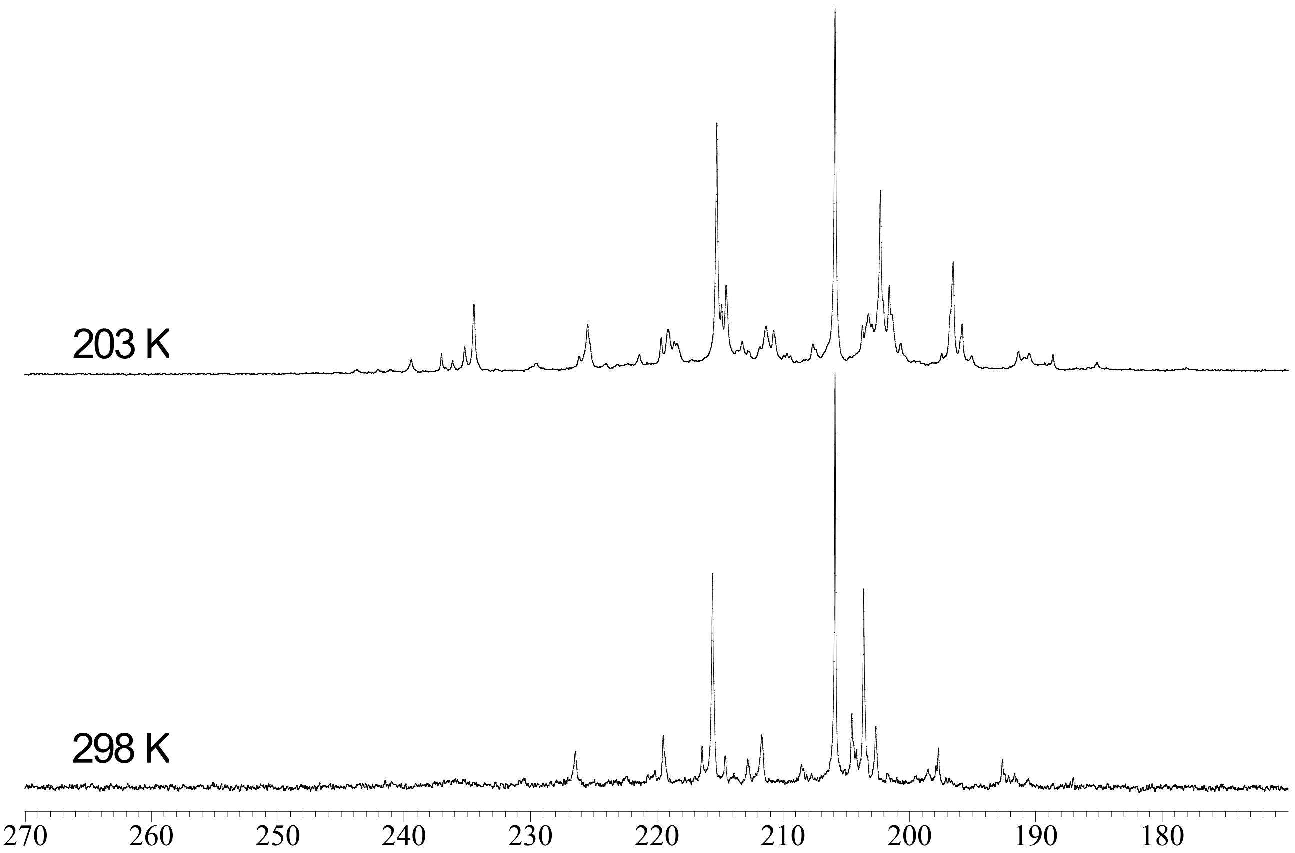

Figure S49

VT ^{195}Pt NMR spectra of $[NBu_4]_2[Pt_{\sim 2}Ni_{\sim 4}(CO)_{12}]$ in CD_3COCD_3.

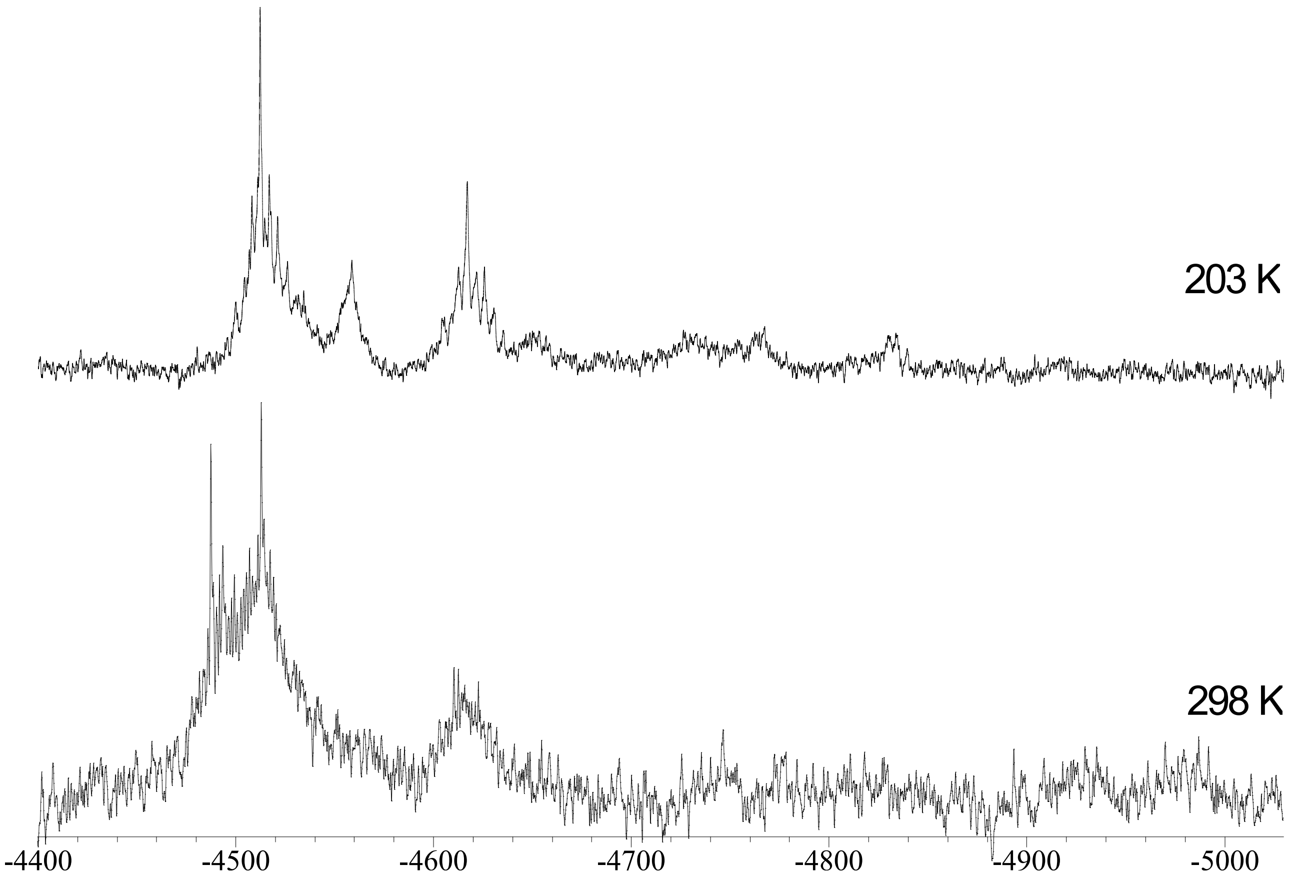

Figure S50

VT ^{13}C NMR spectra of $[NBu_4]_2[Pt_{\sim2}Ni_{\sim4}(CO)_{12}]$ in CD_3COCD_3.

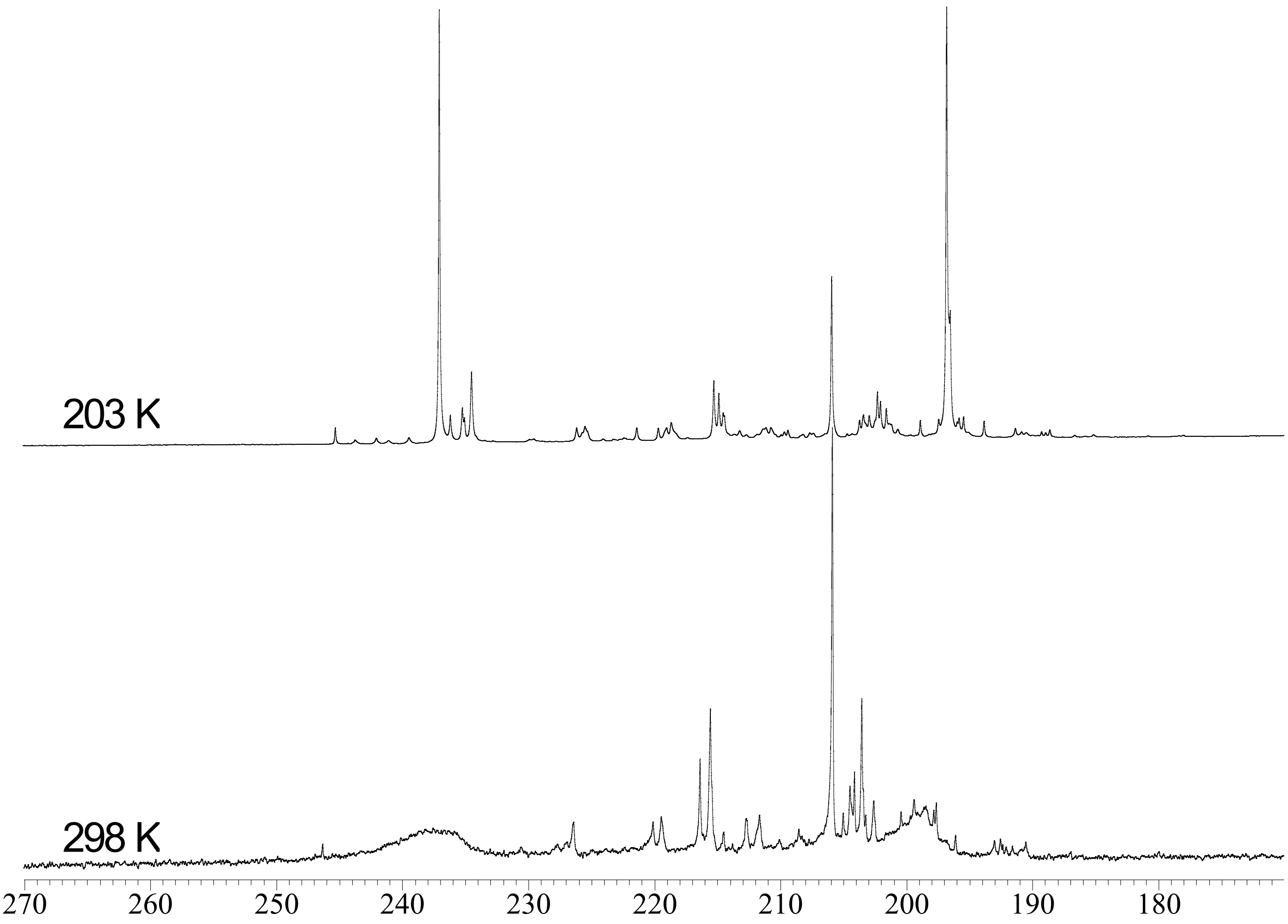

Figure S51

Possible isomers of $[Pt_{6-x}Ni_x(CO)_{12}]^{2-}$ (x = 0 – 6). Isomers **1-10** are inter-converted by a combination of inter-molecular triangle exchange reactions and intra-molecular CO exchange. Isomers 4/4’, 5/5’ and 6/6’ are inter-converted by intra-molecular triangle rotation.

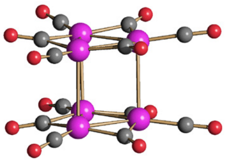

2-$(Pt_3)(Pt_3)$

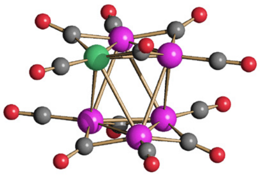

9-$(Pt_3)(Pt_2Ni)$

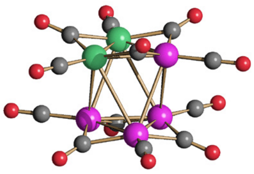

7-$(Pt_3)(PtNi_2)$

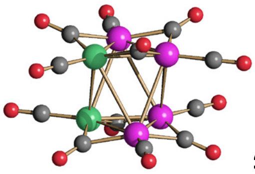

5-$(Pt_2Ni)(Pt_2Ni)$

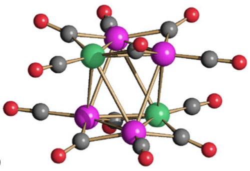

5’-$(Pt_2Ni)(Pt_2Ni)$

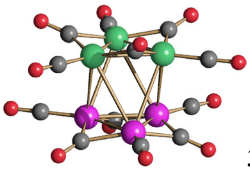

3-$(Pt_3)(Ni_3)$

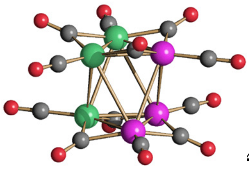

4-$(Pt_2Ni)(PtNi_2)$

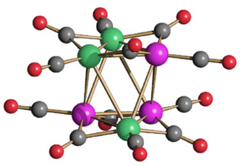

4’-$(Pt_2Ni)(PtNi_2)$

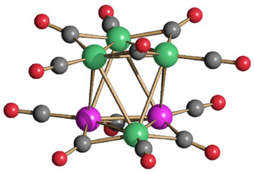

8-$(Ni_3)(Pt_2Ni)$

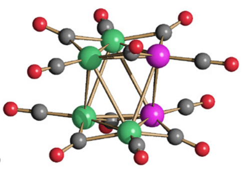

6-$(PtNi_2)(PtNi_2)$

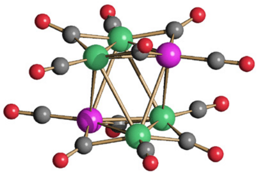

6’-$(PtNi_2)(PtNi_2)$

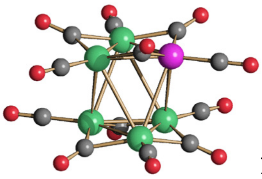

10-$(Ni_3)(PtNi_2)$

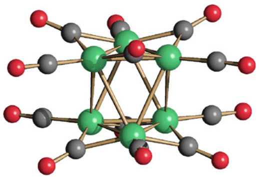

1-$(Ni_3)(Ni_3)$

Figure S52

Triangle exchange reactions between $[Pt_{6-x}Ni_x(CO)_{12}]^{2-}$ (x = 0 – 6) clusters.

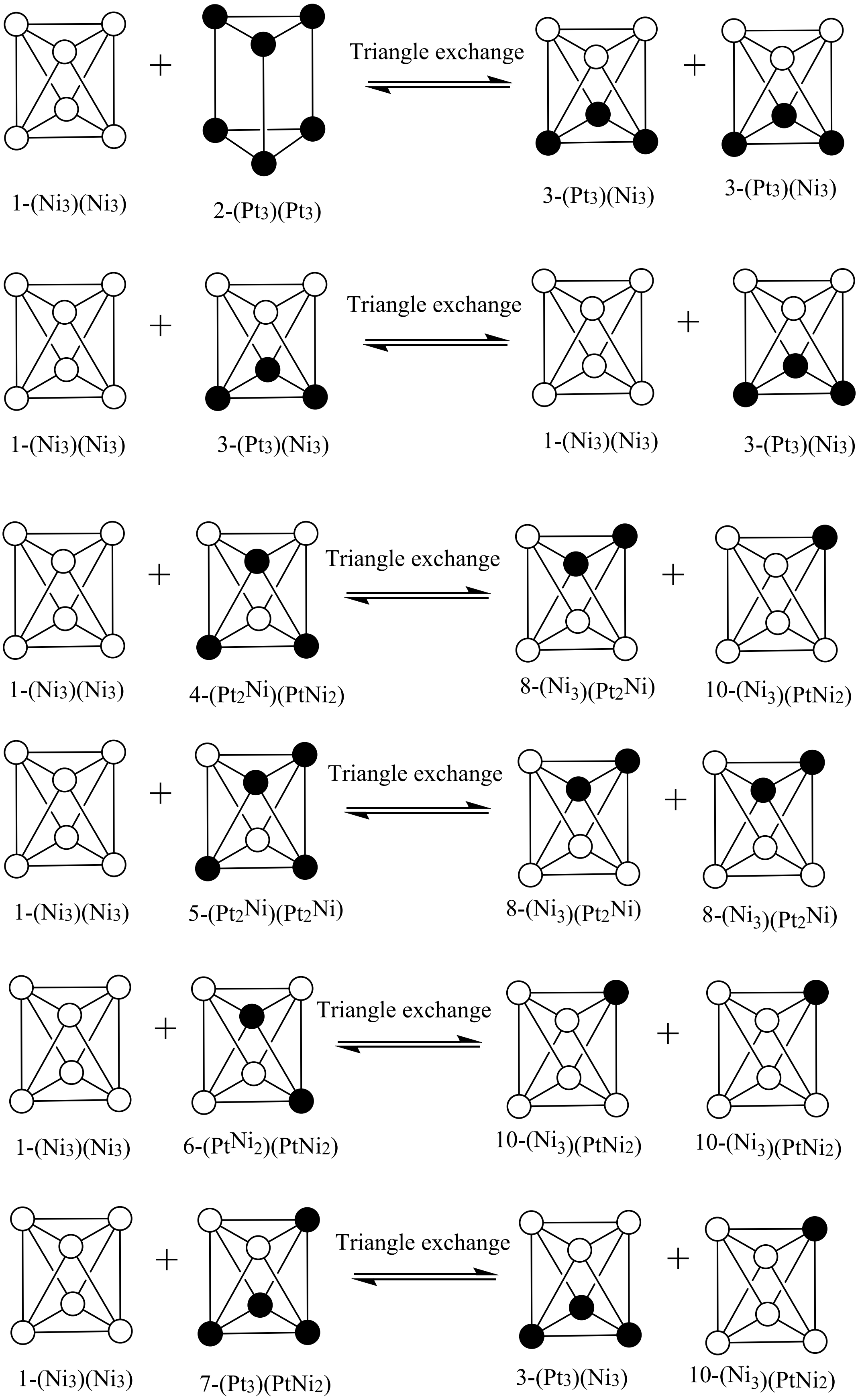

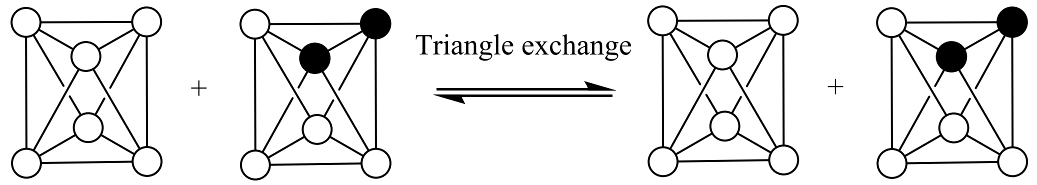

1-$(Ni_3)(Ni_3)$ 8-$(Ni_3)(Pt_2Ni)$ 1-$(Ni_3)(Ni_3)$ 8-$(Ni_3)(Pt_2Ni)$

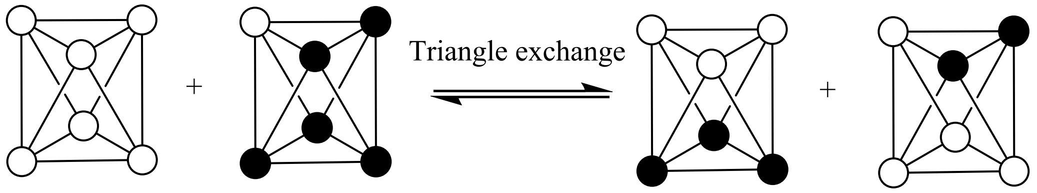

1-$(Ni_3)(Ni_3)$ 9-$(Pt_3)(Pt_2Ni)$ 3-$(Pt_3)(Ni_3)$ 8-$(Ni_3)(Pt_2Ni)$

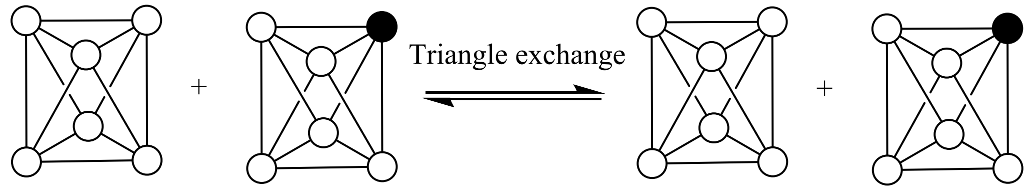

1-$(Ni_3)(Ni_3)$ 10-$(Ni_3)(PtNi_2)$ 1-$(Ni_3)(Ni_3)$ 10-$(Ni_3)(PtNi_2)$

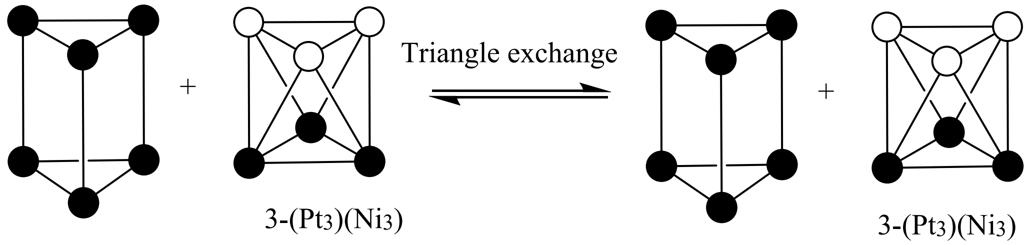

2-$(Pt_3)(Pt_3)$ 3-$(Pt_3)(Ni_3)$ 2-$(Pt_3)(Pt_3)$ 3-$(Pt_3)(Ni_3)$

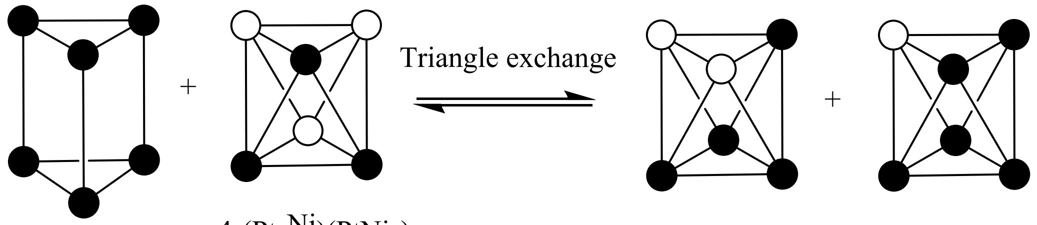

2-$(Pt_3)(Pt_3)$ 4-$(Pt_2Ni)(PtNi_2)$ 7-$(Pt_3)(PtNi_2)$ 9-$(Pt_3)(Pt_2Ni)$

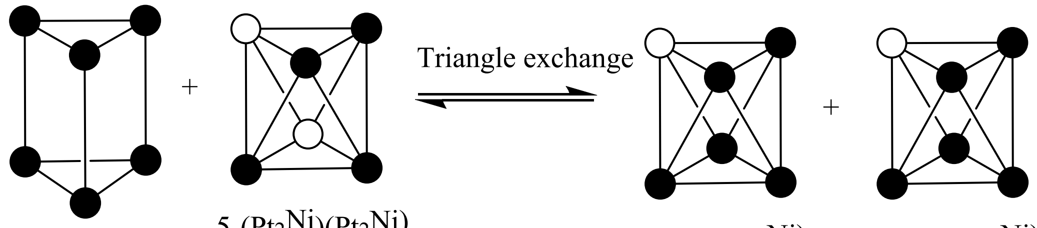

2-$(Pt_3)(Pt_3)$ 5-$(Pt_2Ni)(Pt_2Ni)$ 9-$(Pt_3)(Pt_2Ni)$ 9-$(Pt_3)(Pt_2Ni)$

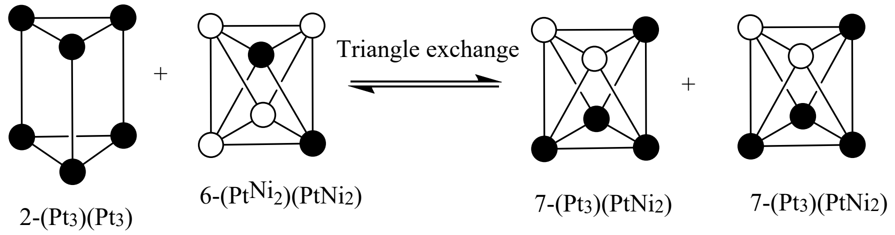
2-(Pt_3)(Pt_3)
+
6-($PtNi_2$)($PtNi_2$)
Triangle exchange
7-(Pt_3)($PtNi_2$)
+
7-(Pt_3)($PtNi_2$)

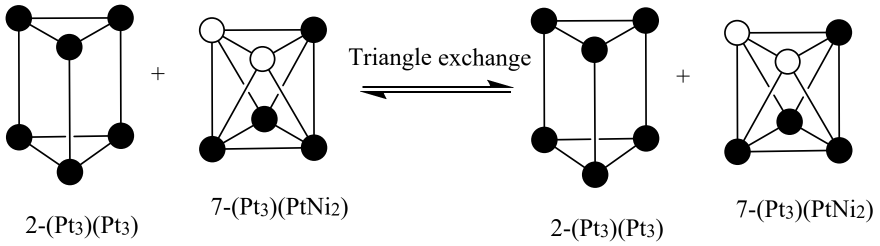
2-(Pt_3)(Pt_3)
+
7-(Pt_3)($PtNi_2$)
Triangle exchange
2-(Pt_3)(Pt_3)
+
7-(Pt_3)($PtNi_2$)

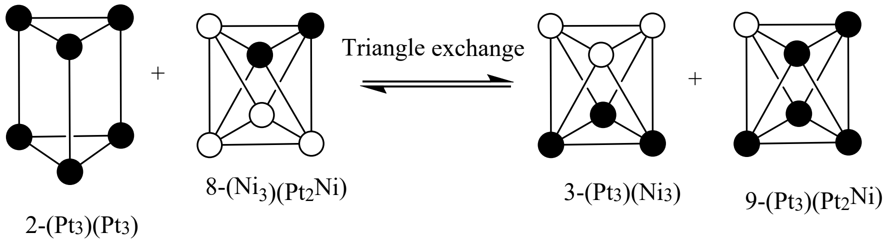
2-(Pt_3)(Pt_3)
+
8-(Ni_3)(Pt_2Ni)
Triangle exchange
3-(Pt_3)(Ni_3)
+
9-(Pt_3)(Pt_2Ni)

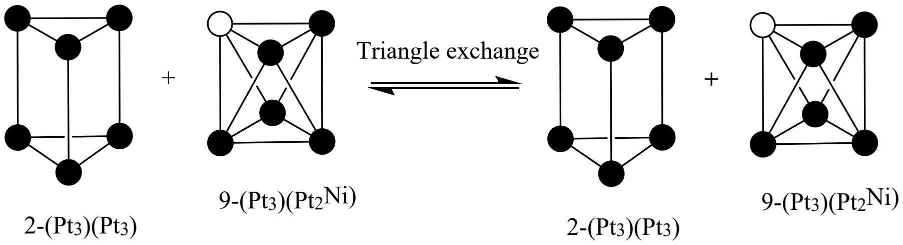
2-(Pt_3)(Pt_3)
+
9-(Pt_3)(Pt_2Ni)
Triangle exchange
2-(Pt_3)(Pt_3)
+
9-(Pt_3)(Pt_2Ni)

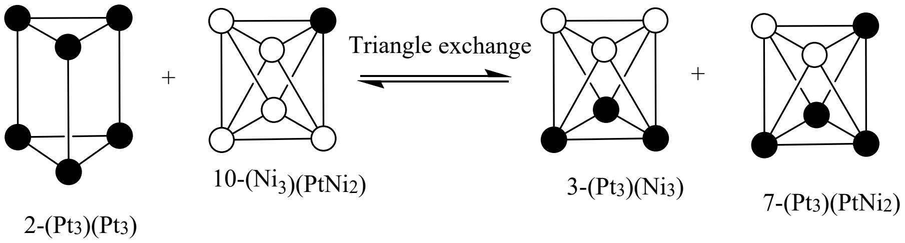
2-(Pt_3)(Pt_3)
+
10-(Ni_3)($PtNi_2$)
Triangle exchange
3-(Pt_3)(Ni_3)
+
7-(Pt_3)($PtNi_2$)

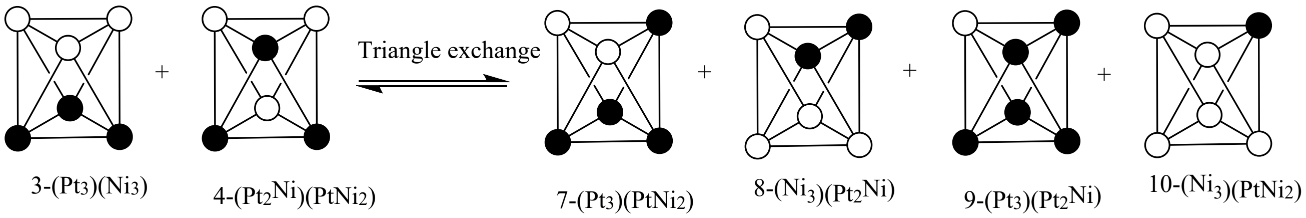
3-(Pt_3)(Ni_3)
+
4-(Pt_2Ni)($PtNi_2$)
Triangle exchange
7-(Pt_3)($PtNi_2$)
+
8-(Ni_3)(Pt_2Ni)
+
9-(Pt_3)(Pt_2Ni)
+
10-(Ni_3)($PtNi_2$)

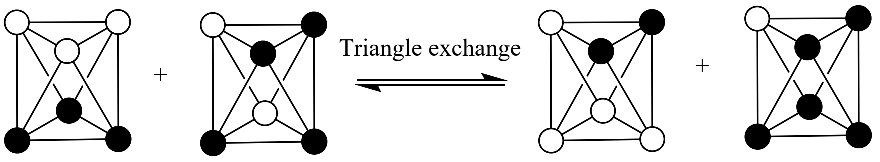

3-(Pt_3)(Ni_3) + 5-(Pt_2Ni)(Pt_2Ni) ⇌ 8-(Ni_3)(Pt_2Ni) + 9-(Pt_3)(Pt_2Ni)

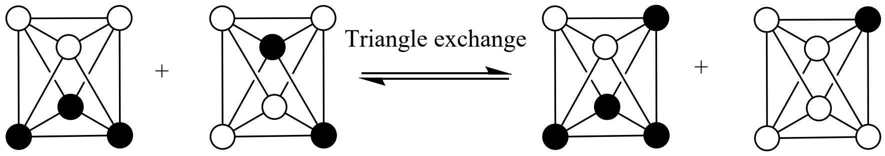

3-(Pt_3)(Ni_3) + 6-($PtNi_2$)($PtNi_2$) ⇌ 7-(Pt_3)($PtNi_2$) + 10-(Ni_3)($PtNi_2$)

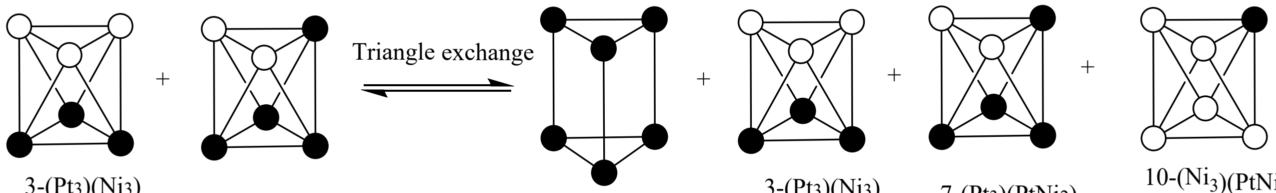

3-(Pt_3)(Ni_3) + 7-(Pt_3)($PtNi_2$) ⇌ 2-(Pt_3)(Pt_3) + 3-(Pt_3)(Ni_3) + 7-(Pt_3)($PtNi_2$) + 10-(Ni_3)($PtNi_2$)

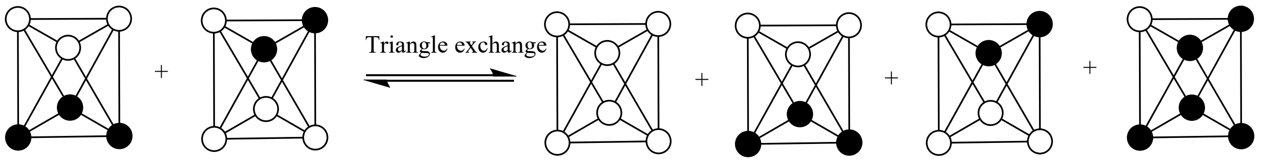

3-(Pt_3)(Ni_3) + 8-(Ni_3)(Pt_2Ni) ⇌ 1-(Ni_3)(Ni_3) + 3-(Pt_3)(Ni_3) + 8-(Ni_3)(Pt_2Ni) + 9-(Pt_3)(Pt_2Ni)

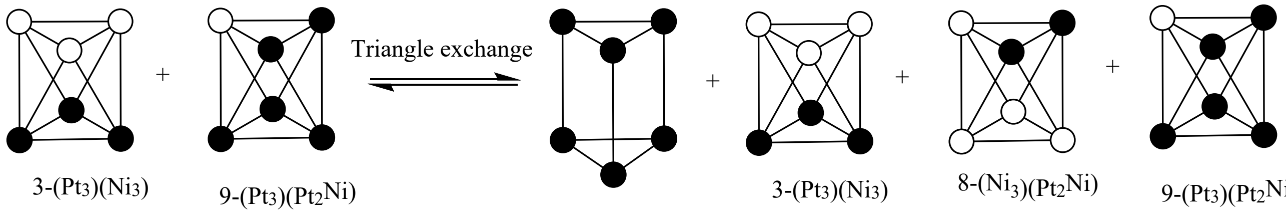

3-(Pt_3)(Ni_3) + 9-(Pt_3)(Pt_2Ni) ⇌ 2-(Pt_3)(Pt_3) + 3-(Pt_3)(Ni_3) + 8-(Ni_3)(Pt_2Ni) + 9-(Pt_3)(Pt_2Ni)

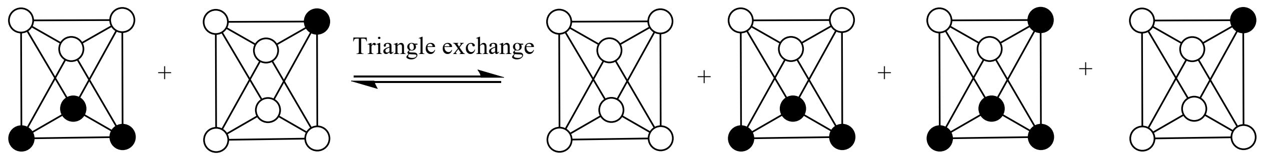

3-(Pt_3)(Ni_3) + 10-(Ni_3)($PtNi_2$) ⇌ 1-(Ni_3)(Ni_3) + 3-(Pt_3)(Ni_3) + 7-(Pt_3)($PtNi_2$) + 10-(Ni_3)($PtNi_2$)

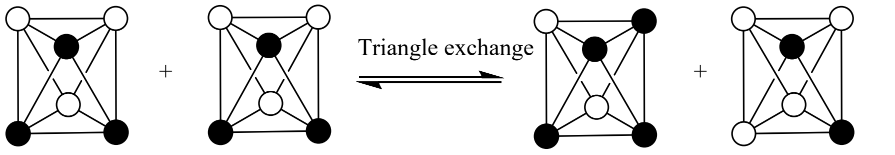

4-(Pt_2Ni)($PtNi_2$) + 4-(Pt_2Ni)($PtNi_2$) ⇌ 5-(Pt_2Ni)(Pt_2Ni) + 6-($PtNi_2$)($PtNi_2$)

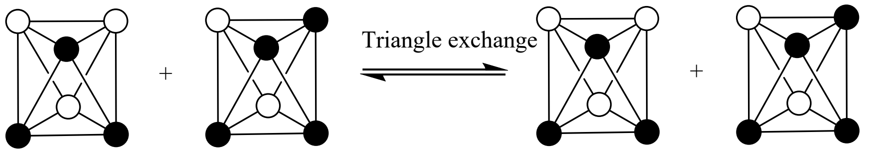

4-(Pt_2Ni)($PtNi_2$) + 5-(Pt_2Ni)(Pt_2Ni) ⇌ 4-(Pt_2Ni)($PtNi_2$) + 5-(Pt_2Ni)(Pt_2Ni)

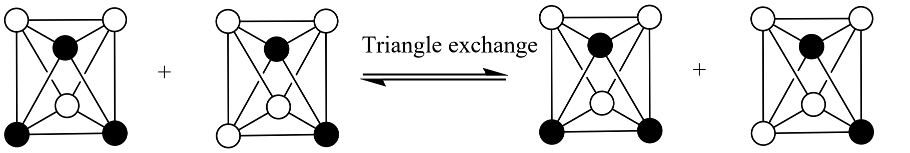

4-(Pt_2Ni)($PtNi_2$) + 6-($PtNi_2$)($PtNi_2$) ⇌ 4-(Pt_2Ni)($PtNi_2$) + 6-($PtNi_2$)($PtNi_2$)

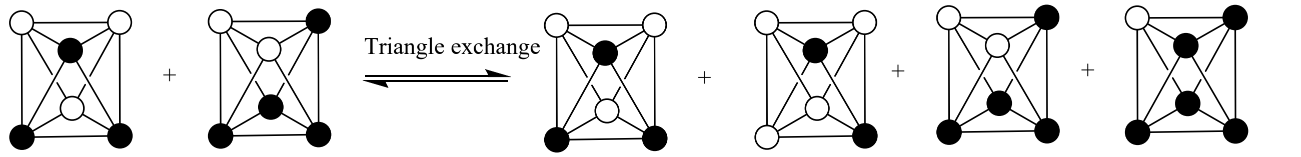

4-(Pt_2Ni)($PtNi_2$) + 7-(Pt_3)($PtNi_2$) ⇌ 4-(Pt_2Ni)($PtNi_2$) + 6-($PtNi_2$)($PtNi_2$) + 7-(Pt_3)($PtNi_2$) + 9-(Pt_3)(Pt_2Ni)

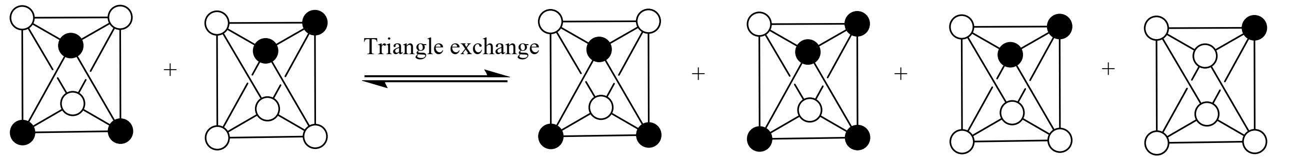

4-(Pt_2Ni)($PtNi_2$) + 8-(Ni_3)(Pt_2Ni) ⇌ 4-(Pt_2Ni)($PtNi_2$) + 5-(Pt_2Ni)(Pt_2Ni) + 8-(Ni_3)(Pt_2Ni) + 10-(Ni_3)($PtNi_2$)

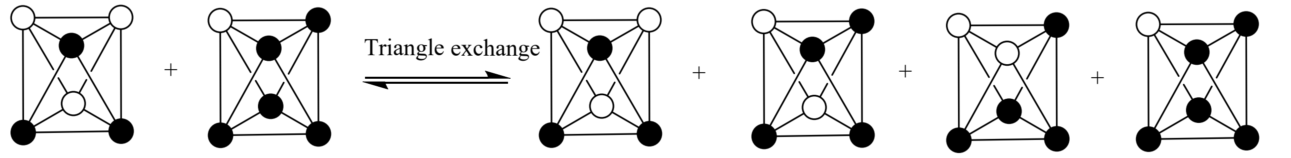

4-(Pt_2Ni)($PtNi_2$) + 9-(Pt_3)(Pt_2Ni) ⇌ 4-(Pt_2Ni)($PtNi_2$) + 5-(Pt_2Ni)(Pt_2Ni) + 7-(Pt_3)($PtNi_2$) + 9-(Pt_3)(Pt_2Ni)

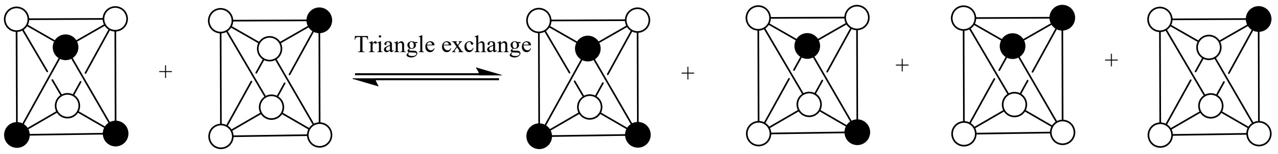

4-(Pt_2Ni)($PtNi_2$) + 10-(Ni_3)($PtNi_2$) ⇌ 4-(Pt_2Ni)($PtNi_2$) + 6-($PtNi_2$)($PtNi_2$) + 8-(Ni_3)(Pt_2Ni) + 10-(Ni_3)($PtNi_2$)

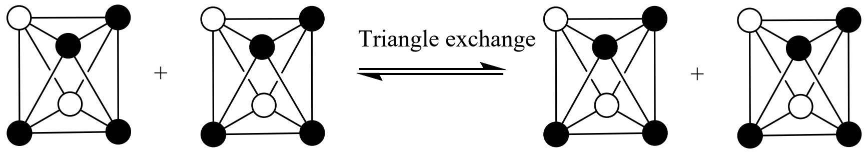

5-(Pt_2Ni)(Pt_2Ni) + 5-(Pt_2Ni)(Pt_2Ni) ⇌ 5-(Pt_2Ni)(Pt_2Ni) + 5-(Pt_2Ni)(Pt_2Ni)

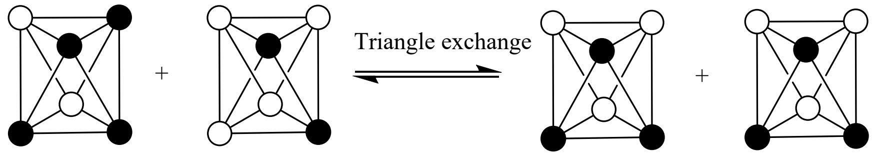

5-(Pt_2Ni)(Pt_2Ni) + 6-($PtNi_2$)($PtNi_2$) ⇌ 4-(Pt_2Ni)($PtNi_2$) + 4-(Pt_2Ni)($PtNi_2$)

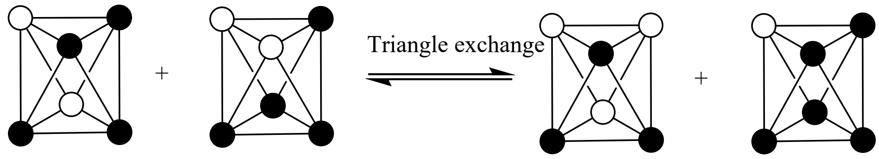

5-(Pt_2Ni)(Pt_2Ni) + 7-(Pt_3)($PtNi_2$) ⇌ 4-(Pt_2Ni)($PtNi_2$) + 9-(Pt_3)(Pt_2Ni)

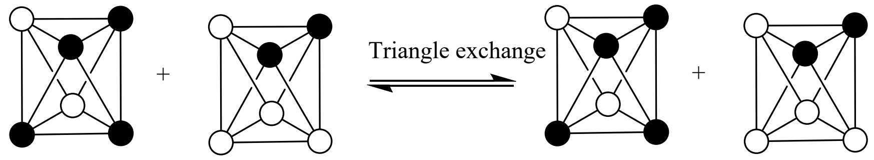

5-(Pt_2Ni)(Pt_2Ni) + 8-(Ni_3)(Pt_2Ni) ⇌ 5-(Pt_2Ni)(Pt_2Ni) + 8-(Ni_3)(Pt_2Ni)

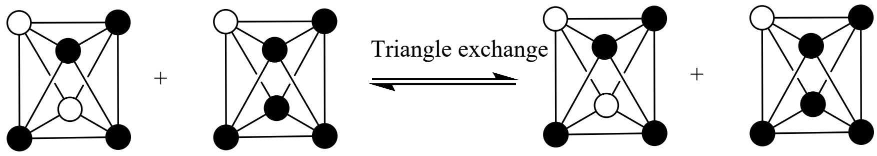

5-(Pt_2Ni)(Pt_2Ni) + 9-(Pt_3)(Pt_2Ni) ⇌ 5-(Pt_2Ni)(Pt_2Ni) + 9-(Pt_3)(Pt_2Ni)

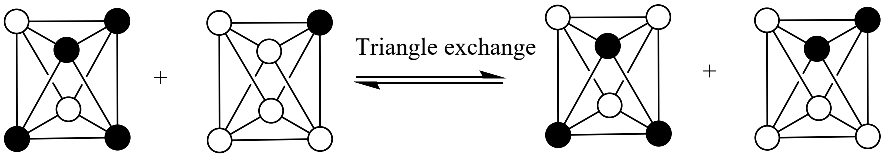

5-$(Pt_2Ni)(Pt_2Ni)$ + 10-$(Ni_3)(PtNi_2)$ ⇌ 4-$(Pt_2Ni)(PtNi_2)$ + 8-$(Ni_3)(Pt_2Ni)$

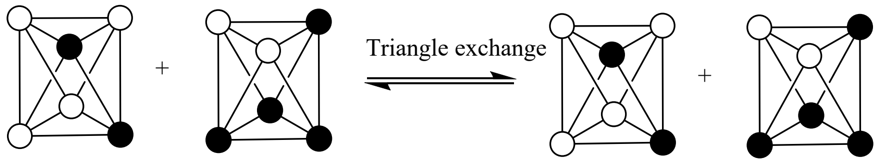

6-$(PtNi_2)(PtNi_2)$ + 7-$(Pt_3)(PtNi_2)$ ⇌ 6-$(PtNi_2)(PtNi_2)$ + 7-$(Pt_3)(PtNi_2)$

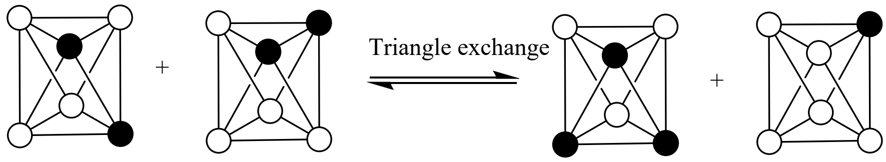

6-$(PtNi_2)(PtNi_2)$ + 8-$(Ni_3)(Pt_2Ni)$ ⇌ 4-$(Pt_2Ni)(PtNi_2)$ + 10-$(Ni_3)(PtNi_2)$

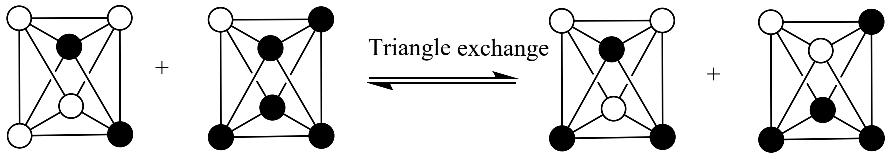

6-$(PtNi_2)(PtNi_2)$ + 9-$(Pt_3)(Pt_2Ni)$ ⇌ 4-$(Pt_2Ni)(PtNi_2)$ + 7-$(Pt_3)(PtNi_2)$

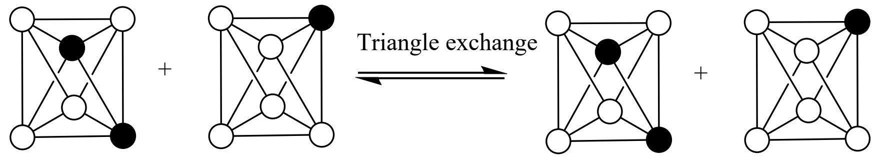

6-$(PtNi_2)(PtNi_2)$ + 10-$(Ni_3)(PtNi_2)$ ⇌ 6-$(PtNi_2)(PtNi_2)$ + 10-$(Ni_3)(PtNi_2)$

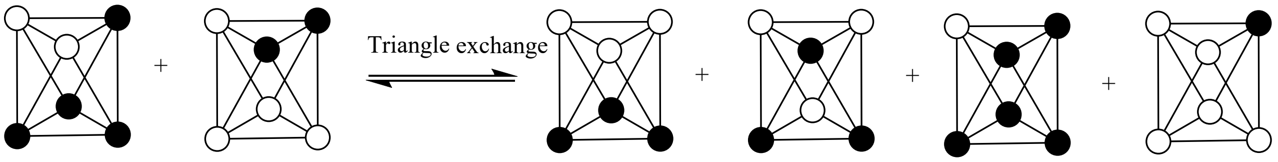

7-$(Pt_3)(PtNi_2)$ + 8-$(Ni_3)(Pt_2Ni)$ ⇌ 3-$(Pt_3)(Ni_3)$ + 4-$(Pt_2Ni)(PtNi_2)$ + 9-$(Pt_3)(Pt_2Ni)$ + 10-$(Ni_3)(PtNi_2)$

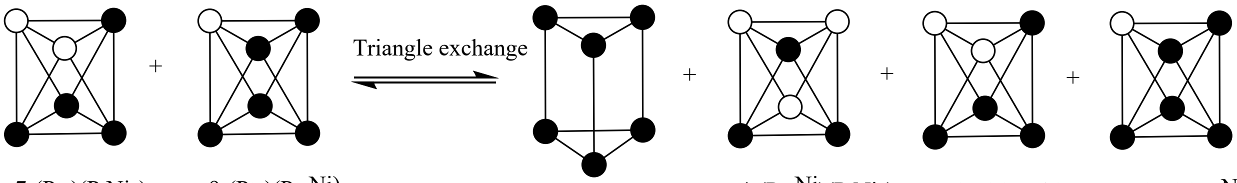

7-(Pt$_3$)(PtNi$_2$) + 9-(Pt$_3$)(Pt$_2$Ni) ⇌ 2-(Pt$_3$)(Pt$_3$) + 4-(Pt$_2$Ni)(PtNi$_2$) + 7-(Pt$_3$)(PtNi$_2$) + 9-(Pt$_3$)(Pt$_2$Ni)

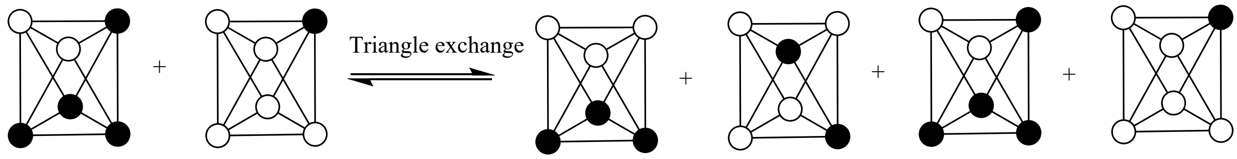

7-(Pt$_3$)(PtNi$_2$) + 10-(Ni$_3$)(PtNi$_2$) ⇌ 3-(Pt$_3$)(Ni$_3$) + 6-(PtNi$_2$)(PtNi$_2$) + 7-(Pt$_3$)(PtNi$_2$) + 10-(Ni$_3$)(PtNi$_2$)

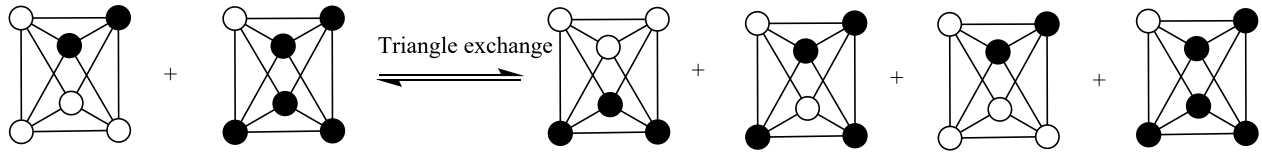

8-(Ni$_3$)(Pt$_2$Ni) + 9-(Pt$_3$)(Pt$_2$Ni) ⇌ 3-(Pt$_3$)(Ni$_3$) + 5-(Pt$_2$Ni)(Pt$_2$Ni) + 8-(Ni$_3$)(Pt$_2$Ni) + 9-(Pt$_3$)(Pt$_2$Ni)

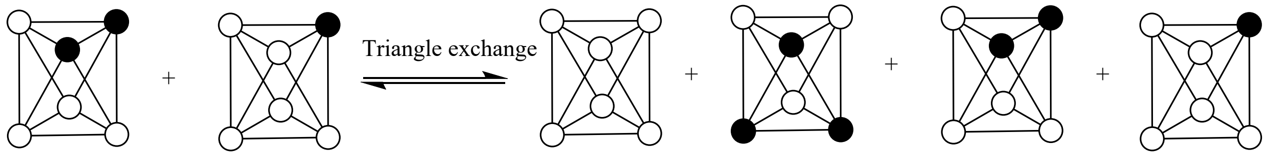

8-(Ni$_3$)(Pt$_2$Ni) + 10-(Ni$_3$)(PtNi$_2$) ⇌ 1-(Ni$_3$)(Ni$_3$) + 4-(Pt$_2$Ni)(PtNi$_2$) + 8-(Ni$_3$)(Pt$_2$Ni) + 10-(Ni$_3$)(PtNi$_2$)

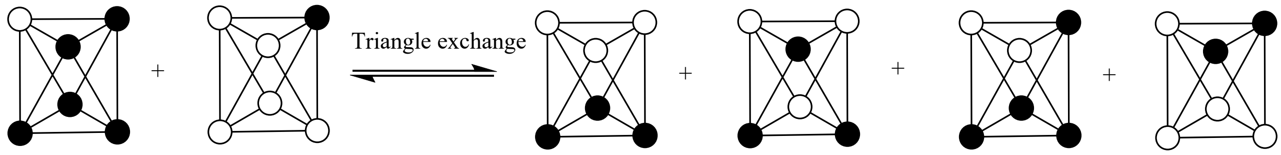

9-(Pt$_3$)(Pt$_2$Ni) + 10-(Ni$_3$)(PtNi$_2$) ⇌ 3-(Pt$_3$)(Ni$_3$) + 4-(Pt$_2$Ni)(PtNi$_2$) + 7-(Pt$_3$)(PtNi$_2$) + 8-(Ni$_3$)(Pt$_2$Ni)

Figure S53

Isomerization by CO migration of $[Pt_{6-x}Ni_x(CO)_{12}]^{2-}$ (x = 2 – 4): General mechanism for $[Pt_3Ni_3(CO)_{12}]^{2-}$, $[Pt_4Ni_2(CO)_{12}]^{2-}$ and $[Pt_2Ni_4(CO)_{12}]^{2-}$(only μ-CO are represented).

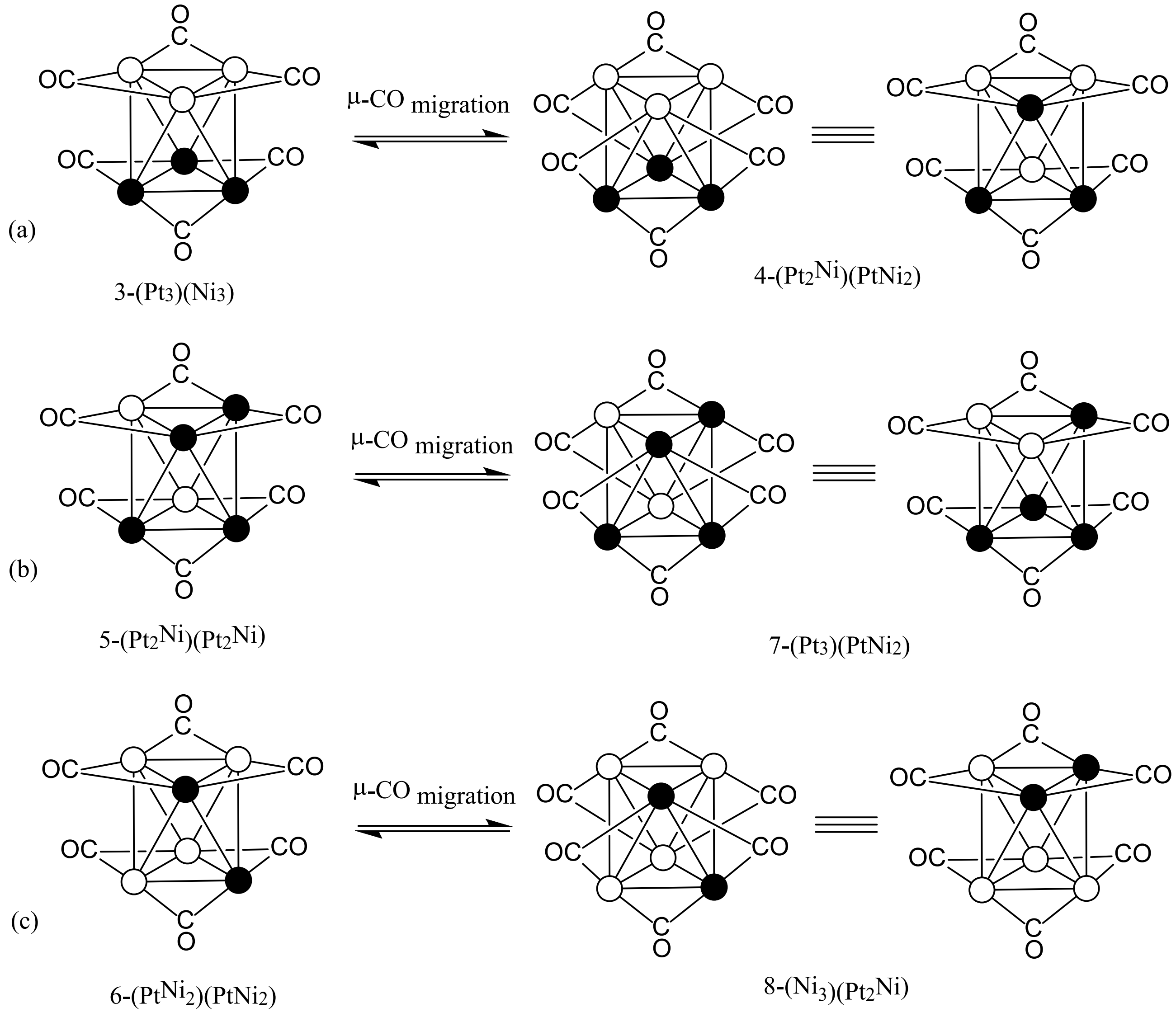

Figure S54

Isomerization by CO migration of $[Pt_{6-x}Ni_x(CO)_{12}]^{2-}$ (x = 2 – 4): Schematic representation for $[Pt_3Ni_3(CO)_{12}]^{2-}$, $[Pt_4Ni_2(CO)_{12}]^{2-}$ and $[Pt_2Ni_4(CO)_{12}]^{2-}$ (all CO ligands are omitted).

(a)

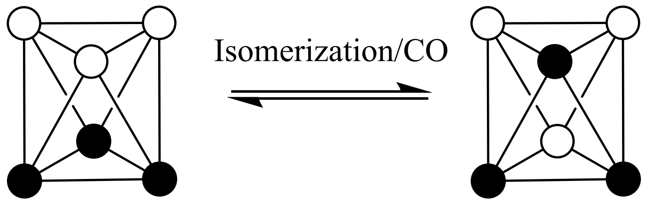

3-(Pt3)(Ni3) 4-(Pt2Ni)(PtNi2)

(b)

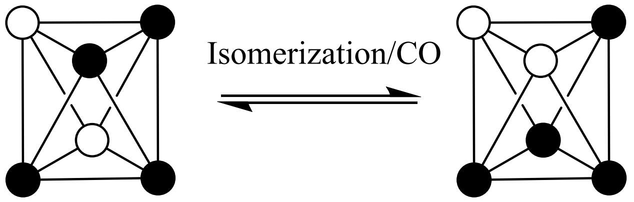

5-(Pt2Ni)(Pt2Ni) 7-(Pt3)(PtNi2)

(c)

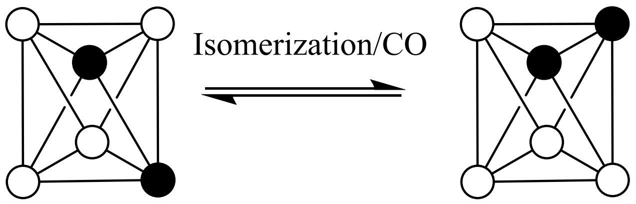

6-(PtNi2)(PtNi2) 8-(Ni3)(Pt2Ni)

Figure S55

Isomerization by triangle rotation of $[Pt_{6-x}Ni_x(CO)_{12}]^{2-}$ (x = 2 – 4).

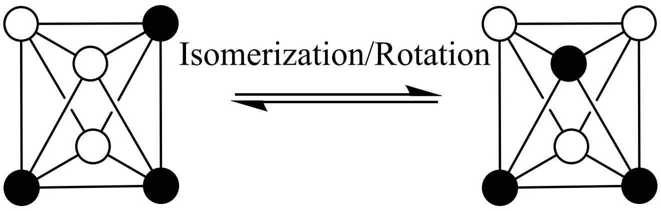

4'-(Pt_2Ni)($PtNi_2$)   4-(Pt_2Ni)($PtNi_2$)

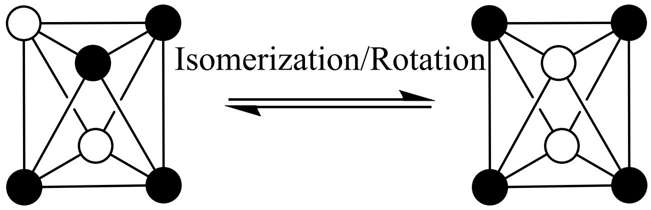

5-(Pt_2Ni)(Pt_2Ni)   5'-(Pt_2Ni)(Pt_2Ni)

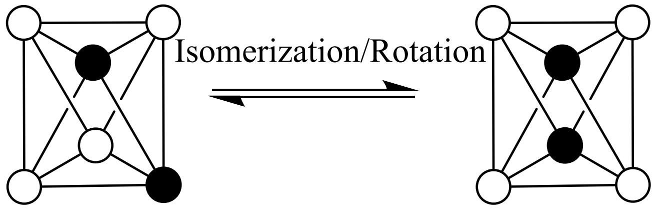

6-($PtNi_2$)($PtNi_2$)   6'-($PtNi_2$)($PtNi_2$)

X-ray Crystallographic Study

Crystal data and collection details for $[NBu_4]_2[Pt_{6-x}Ni_x(CO)_{12}]$ (x = 1.25), $[NBu_4]_2[Pt_{6-x}Ni_x(CO)_{12}]$ (x = 3.24), $[NBu_4]_2[Pt_{6-x}Ni_x(CO)_{12}]$ (x = 4.15), $[NBu_4]_2[Pt_{6-x}Ni_x(CO)_{12}]$ (x = 4.16), $[NBu_4]_2[Pt_{6-x}Ni_x(CO)_{12}]$ (x = 4.41), $[NBu_4]_2[Pt_{6-x}Ni_x(CO)_{12}]$ (x = 5.78), $[NBu_4]_2[Pt_{6-x}Ni_x(CO)_{12}]$ (x = 5.90), $[NBu_4]_4[Pt_{6-x}Ni_x(CO)_{12}][Cl_{1.77}Br_{0.23}]$ (x = 2.53), $[NBu_4]_2[Pt_9(CO)_{18}]$·thf, and $[NBu_4]_2[Pt_6(CO)_{12}]$ are reported in Table S5. The diffraction experiments were carried out on a Bruker APEX II diffractometer equipped with a PHOTON2 detector using Mo–Kα radiation. Data were corrected for Lorentz polarization and absorption effects (empirical absorption correction SADABS).[1] Structures were solved by direct methods and refined by full-matrix least-squares based on all data using F^2.[2] Hydrogen atoms were fixed at calculated positions and refined by a riding model. All non-hydrogen atoms were refined with anisotropic displacement parameters, unless otherwise stated.

$[NBu_4]_2[Pt_{6-x}Ni_x(CO)_{12}]$ (x = 1.25): The asymmetric unit of the unit cell contains half of a cluster anion (located on an inversion centre) and one $[NBu_4]^+$ cation (located on a general position). The positions occupied by M(1), M(2) and M(3) are disordered Pt/Ni. These have been refined applying dummy atoms constraints (EADP and EXYZ lines in SHLEXL) resulting in the following refined occupancy factors: M(1) = 0.839(4) Pt and 0.161(4) Ni; M(2) = 0.810(4) Pt and 0.190(4) Ni; M(3) = 0.723(4) Pt and 0.277(4) Ni.

$[NBu_4]_2[Pt_{6-x}Ni_x(CO)_{12}]$ (x = 3.24): The asymmetric unit of the unit cell contains half of a cluster anion (located on an inversion centre) and one $[NBu_4]^+$ cation (located on a general position). The positions occupied by M(1), M(2) and M(3) are disordered Pt/Ni. These have been refined applying dummy atoms constraints (EADP and EXYZ lines in SHLEXL) resulting in the following refined occupancy factors: M(1) = 0.532(4) Pt and 0.468(4) Ni; M(2) = 0.484(4) Pt and 0.516(4) Ni; M(3) = 0.367(4) Pt and 0.633(4) Ni.

$[NBu_4]_2[Pt_{6-x}Ni_x(CO)_{12}]$ (x = 4.15): The asymmetric unit of the unit cell contains half of a cluster anion (located on an inversion centre) and one $[NBu_4]^+$ cation (located on a general position). The positions occupied by M(1), M(2) and M(3) are disordered Pt/Ni. These have been refined applying dummy atoms constraints (EADP and EXYZ lines in SHLEXL) resulting in the following refined occupancy factors: M(1) = 0.3724(16) Pt and 0.6276(16) Ni; M(2) = 0.3563(16) Pt and 0.6437(16) Ni; M(3) = 0.1972(4) Pt and 0.8028(14) Ni.

$[NBu_4]_2[Pt_{6-x}Ni_x(CO)_{12}]$ (x = 4.16): The asymmetric unit of the unit cell contains half of a cluster anion (located on an inversion centre) and one $[NBu_4]^+$ cation (located on a general position). The positions occupied by M(1), M(2) and M(3) are disordered Pt/Ni. These have been refined applying dummy atoms constraints (EADP and EXYZ lines in SHLEXL) resulting in the following refined

occupancy factors: M(1) = 0.3670(17) Pt and 0.6330(17) Ni; M(2) = 0.3482(18) Pt and 0.6518(18) Ni; M(3) = 0.2039(15) Pt and 0.7961(15) Ni.

$[NBu_4]_2[Pt_{6-x}Ni_x(CO)_{12}]$ (x = 4.41): The asymmetric unit of the unit cell contains half of a cluster anion (located on an inversion centre) and one $[NBu_4]^+$ cation (located on a general position). The positions occupied by M(1), M(2) and M(3) are disordered Pt/Ni. These have been refined applying dummy atoms constraints (EADP and EXYZ lines in SHLEXL) resulting in the following refined occupancy factors: M(1) = 0.331(4) Pt and 0.669(4) Ni; M(2) = 0.315(4) Pt and 0.685(4) Ni; M(3) = 0.165(4) Pt and 0.835(4) Ni.

$[NBu_4]_2[Pt_{6-x}Ni_x(CO)_{12}]$ (x = 5.78): The asymmetric unit of the unit cell contains half of a cluster anion (located on an inversion centre) and one $[NBu_4]^+$ cation (located on a general position). The positions occupied by M(1), M(2) and M(3) are disordered Pt/Ni. These have been refined applying dummy atoms constraints (EADP and EXYZ lines in SHLEXL) resulting in the following refined occupancy factors: M(1) = 0.063(14) Pt and 0.9370(14) Ni; M(2) = 0.0385(14) Pt and 0.9615(13) Ni; M(3) = 0.0049(13) Pt and 0.9951(13) Ni.

$[NBu_4]_2[Pt_{6-x}Ni_x(CO)_{12}]$ (x = 5.90): The asymmetric unit of the unit cell contains half of a cluster anion (located on an inversion centre) and one $[NBu_4]^+$ cation (located on a general position). The positions occupied by M(1) and M(2) are disordered Pt/Ni. These have been refined applying dummy atoms constraints (EADP and EXYZ lines in SHLEXL) resulting in the following refined occupancy factors: M(1) = 0.028(7) Pt and 0.9720(7) Ni; M(2) = 0.0179(7) Pt and 0.9821(7) Ni.

$[NBu_4]_4[Pt_{6-x}Ni_x(CO)_{12}][Cl_{1.77}Br_{0.23}]$ (x = 2.53): The asymmetric unit of the unit cell contains half of a cluster anion (located on an inversion centre), two $[NBu_4]^+$ cations and one X^- anion (located on a general position). The positions occupied by M(1), M(2) and M(3) are disordered Pt/Ni, and the position occupied by X^- is disordered Br/Cl. These have been refined applying dummy atoms constraints (EADP and EXYZ lines in SHLEXL) resulting in the following refined occupancy factor: M(1) = 0.661(3) Pt and 0.339(3) Ni; M(2) = 0.584(3) Pt and 0.416(3) Ni; M(3) = 0.492(3) Pt and 0.508(3) Ni; X = 0.112(4) Br and 0.888(4) Cl. All C, O and N atoms have been restrained to have similar *U* parameters (SIMU line in SHELXL, s.u. 0.005) and isotropic like behaviour (ISOR line in SHELXL, s.u. 0.005).

$[NBu_4]_2[Pt_9(CO)_{18}]\cdot$thf: The asymmetric unit of the unit cell contains one cluster anion, two $[NBu_4]^+$ cations and one thf molecule all located on general positions. Since this compound was obtained by the reaction of $[NBu_4]_2[Pt_{12}(CO)_{24}]$ with $[NBu_4]_2[Ni_6(CO)_{12}]$, during the initial refinement all the M sites were tested for the possible presence of Pt/Ni disorder. Nonetheless, for all positions, the refinement pointed out the exclusive presence of Pt. Thus, in the final refinement, only Pt-atoms were included in the cluster. One $[NBu_4]^+$ cation and one Bu-group in the other

cation are disordered and, therefore, they have been split into two positions and refined anisotropically employing one occupancy factor per disordered group. The $[NBu_4]^+$ cations have been restrained to have similar thermal parameters (SIMU line in SHELXL, s.u. 0.01). The thf molecule has been restrained to isotropic behaviour (ISOR line in SHELXL, s.u. 0.01). Restraints to bond distances were applied as follow (s.u. 0.02): 1.47 Å for C–N and 1.53 Å for C–C in $[NBu_4]^+$.

$[NBu_4]_2[Pt_6(CO)_{12}]$: The asymmetric unit of the unit cell contains half of a cluster anion (located on a 2-fold axis), and one $[NBu_4]^+$ cation (located on a general position).

Table S5

Crystal data and experimental details for $[NBu_4]_2[Pt_{6-x}Ni_x(CO)_{12}]$ (x = 1.25), $[NBu_4]_2[Pt_{6-x}Ni_x(CO)_{12}]$ (x = 3.24), $[NBu_4]_2[Pt_{6-x}Ni_x(CO)_{12}]$ (x = 4.15), $[NBu_4]_2[Pt_{6-x}Ni_x(CO)_{12}]$ (x = 4.16), $[NBu_4]_2[Pt_{6-x}Ni_x(CO)_{12}]$ (x = 4.41),$[NBu_4]_2[Pt_{6-x}Ni_x(CO)_{12}]$ (x = 5.78),$[NBu_4]_2[Pt_{6-x}Ni_x(CO)_{12}]$ (x = 5.90), $[NBu_4]_4[Pt_{6-x}Ni_x(CO)_{12}][Cl_{1.77}Br_{0.23}]$ (x = 2.53), $[NBu_4]_2[Pt_9(CO)_{18}]$·thf, and $[NBu_4]_2[Pt_6(CO)_{12}]$

	**$[NBu_4]_2[Pt_{6-x}Ni_x(CO)_{12}]$ (x = 1.25)**	**$[NBu_4]_2[Pt_{6-x}Ni_x(CO)_{12}]$ (x = 3.24)**	**$[NBu_4]_2[Pt_{6-x}Ni_x(CO)_{12}]$ (x = 4.15)**	**$[NBu_4]_2[Pt_{6-x}Ni_x(CO)_{12}]$ (x = 4.16)**
Formula	$C_{44}H_{72}N_2Ni_{1.25}O_{12}Pt_{4.75}$	$C_{44}H_{72}N_2Ni_{3.24}O_{12}Pt_{2.76}$	$C_{44}H_{72}N_2Ni_{4.15}O_{12}Pt_{1.85}$	$C_{44}H_{72}N_2Ni_{4.16}O_{12}Pt_{1.84}$
Fw	1820.41	1550.38	1425.59	1424.23
T, K	100(2)	100(2)	100(2)	100(2)
λ, Å	0.71073	0.71073	0.71073	0.71073
Crystal system	Monoclinic	Monoclinic	Triclinic	Triclinic
Space Group	$P2_1/n$	$P2_1/n$	$P\bar{1}$	$P\bar{1}$
a, Å	12.625(5)	12.5005(59)	11.0155(4)	11.0219(3)
b, Å	11.982(4)	11.9264(8)	11.8405(4)	11.8581(3)
c, Å	17.790(7)	17.6604(13)	11.9056(4)	11.9028(3)
α, °	90	90	89.9930(10)	89.9820(10)
β, °	91.427(18)	91.724(3)	64.9450(10)	64.9860(10)
γ, °	90	90	70.9870(10)	70.9560(10)
Cell Volume, $Å^3$	2690.4(17)	2631.7(3)	1312.64(8)	1315.29(6)
Z	2	2	1	1
D_c, g cm^{-3}	2.247	1.956	1.803	1.798
μ, mm^{-1}	12.775	8.510	6.424	6.388
F(000)	1702	1504	706	706
Crystal size, mm	0.18×0.15×0.12	0.18×0.16×0.14	0.21×0.18×0.15	0.19×0.16×0.14
θ limits, °	2.049–26.999	1.968–25.997	2.298–27.000	1.841–26.999

Index ranges	$-16 \le h \le 16$<br>$-15 \le k \le 15$<br>$-22 \le l \le 22$	$-15 \le h \le 15$<br>$-14 \le k \le 14$<br>$-21 \le l \le 21$	$-14 \le h \le 14$<br>$-15 \le k \le 15$<br>$-15 \le l \le 15$	$-14 \le h \le 14$<br>$-15 \le k \le 15$<br>$-15 \le l \le 15$
Reflections collected	36640	25908	20102	19229
Independent reflections	5871 [R_{int} = 0.0371]	5179 [R_{int} = 0.0553]	5720 [R_{int} = 0.0374]	5737 [R_{int} = 0.0331]
Completeness to θ max	99.9%	100.0%	99.9%	100.0%
Data / restraints / parameters	5871 / 0 / 305	5179 / 0 / 305	5720/ 1 / 305	5737 / 1 / 305
Goodness on fit on F^2	1.262	1.184	1.079	1.167
R_1 ($I > 2\sigma(I)$)	0.0238	0.0411	0.0219	0.0275
wR_2 (all data)	0.0517	0.0840	0.0506	0.0535
Largest diff. peak and hole, e $Å^{-3}$	1.199 / –0.959	1.371 / –1.057	0.658 / –0.845	0.704 / –1.016

	**$[NBu_4]_2[Pt_{6-x}Ni_x(CO)_{12}]$ (x = 4.41)**	**$[NBu_4]_2[Pt_{6-x}Ni_x(CO)_{12}]$ (x = 5.78)**	**$[NBu_4]_2[Pt_{6-x}Ni_x(CO)_{12}]$ (x = 5.90)**	**$[NBu_4]_4[Pt_{6-x}Ni_x(CO)_{12}][Cl_{1.77}Br_{0.23}]$ (x = 2.53)**
Formula	$C_{44}H_{72}N_2Ni_{4.41}O_{12}Pt_{1.59}$	$C_{44}H_{72}N_2Ni_{5.78}O_{12}Pt_{0.22}$	$C_{44}H_{72}N_2Ni_{5.90}O_{12}Pt_{0.10}$	$C_{76}H_{144}Br_{0.23}Cl_{1.77}N_4Ni_{2.53}O_{12}Pt_{3.48}$
Fw	1390.14	1203.35	1186.93	2213.03
T, K	100(2)	100(2)	100(2)	100(2)
λ, Å	0.71073	0.71073	0.71073	0.71073
Crystal system	Triclinic	Triclinic	Triclinic	Monoclinic
Space Group	$P\bar{1}$	$P\bar{1}$	$P\bar{1}$	$P2_1/n$
a, Å	11.015(5)	10.9784(8)	11.0218(7)	12.0740(8)
b, Å	11.819(5)	11.7985(9)	11.8443(7)	31.835(2)
c, Å	11.916(6)	11.8483(9)	11.8632(7)	12.3924(8)
α, °	89.82(2)	89.202(3)	89.085(2)	90
β, °	64.944(19)	64.938(2)	64.843(2)	108.155(2)
γ, °	70.764(18)	70.309(2)	70.214(2)	90
Cell Volume, $Å^3$	1309.9(11)	1293.90(17)	1304.07(14)	4526.2(5)
Z	1	1	1	2

D_c, g cm^{-3}	1.762	1.544	1.511	1.624
μ, mm^{-1}	5.837	2.709	2.410	6.069
F(000)	694	625	619	2208
Crystal size, mm	0.15×0.13×0.10	0.16×0.13×0.11	0.18×0.16×0.14	0.22×0.16×0.14
θ limits, °	1.849–26.000	1.919–26.999	1.848–26.999	1.844–27.999
Index ranges	-13 ≤ h ≤ 13<br>-14 ≤ k ≤ 14<br>-14 ≤ l ≤ 14	-14 ≤ h ≤ 14<br>-15 ≤ k ≤ 15<br>-15 ≤ l ≤ 15	-14 ≤ h ≤ 14<br>-15 ≤ k ≤ 15<br>-15 ≤ l ≤15	-15 ≤ h ≤ 15<br>-42 ≤ k ≤ 42<br>-16 ≤ l ≤16
Reflections collected	11021	17671	19609	73113
Independent reflections	5132 [R_{int} = 0.0592]	5629 [R_{int} = 0.0414]	5693 [R_{int} = 0.0266]	10914 [R_{int} = 0.0502]
Completeness to θ max	99.7%	99.9%	100.0%	100.0%
Data / restraints / parameters	5132 / 1 / 305	5629 / 1 / 296	5693/ 1 / 295	10914 / 504 / 455
Goodness on fit on F^2	1.088	1.128	1.142	1.223
R_1 (I > 2σ(I))	0.0565	0.0334	0.0234	0.0350
wR_2 (all data)	0.1109	0.0764	0.0525	0.0734
Largest diff. peak and hole, e $Å^{-3}$	1.291 / –1.295	0.621 / –0.695	0.357 / –0.395	1.758 / –1.545

	**$[NBu_4]_2[Pt_9(CO)_{18}]$·thf**	**$[NBu_4]_2[Pt_6(CO)_{12}]$**
Formula	$C_{54}H_{80}N_2O_{19}Pt_9$	$C_{44}H_{72}N_2O_{12}Pt_6$
Fw	2817.01	1991.57
T, K	100(2)	100(2)
λ, Å	0.71073	0.71073
Crystal system	Orthorhombic	Tetragonal
Space Group	*Pbca*	$I4_1/a$
a, Å	18.4985(14)	17.5967(7)
b, Å	19.5823(13)	17.5967(7)
c, Å	37.554(3)	35.3226(15)

α, °	90	90
β, °	90	90
γ, °	90	90
Cell Volume, Å^3	13603.8(17)	10937.4(10)
Z	8	8
D_c, g cm^{-3}	2.751	2.419
μ, mm^{-1}	18.496	15.344
F(000)	10176	7312
Crystal size, mm	0.16×0.13×0.11	0.18×0.16×0.08
θ limits, °	1.545–26.000	2.002–26.615
Index ranges	$-22 \le h \le 22$<br>$-24 \le k \le 24$<br>$-46 \le l \le 46$	$-22 \le h \le 22$<br>$-22 \le k \le 22$<br>$-44 \le l \le 44$
Reflections collected	175114	105705
Independent reflections	13352 [R_{int} = 0.1292]	5748 [R_{int} = 0.1628]
Completeness to θ max	99.9%	100.0%
Data / restraints / parameters	13352 / 752 / 813	5748 / 0 / 293
Goodness on fit on F^2	1.202	1.072
R_1 ($I > 2\sigma(I)$)	0.0671	0.0382
wR_2 (all data)	0.1322	0.0678
Largest diff. peak and hole, e Å^{-3}	2.240 / –2.345	1.804 / –1.369

Computational details with figures and tables

Full geometry optimizations, optimizations with selected constrained internal coordinates and single-point calculations were carried out *in vacuo* using the hybrid meta-GGA DFT functional TPSS0, with 25% HF exchange,[3] in combination with Ahlrichs' def-2 TZVP basis set, with relativistic ECP for Pt.[4] The "restricted" approach was used in all the cases. Calculations were performed with the ORCA 4.2.0 software.[5] Cartesian coordinates of the DFT-optimized structures are collected in a separated .xyz file.

Figure S56

DFT-optimized (TPSS0/def2-TZVP) trigonal prismatic geometry of $[Pt_6(CO)_{12}]^{2-}$ (a) and octahedral geometry of $[Ni_6(CO)_{12}]^{2-}$ (b).

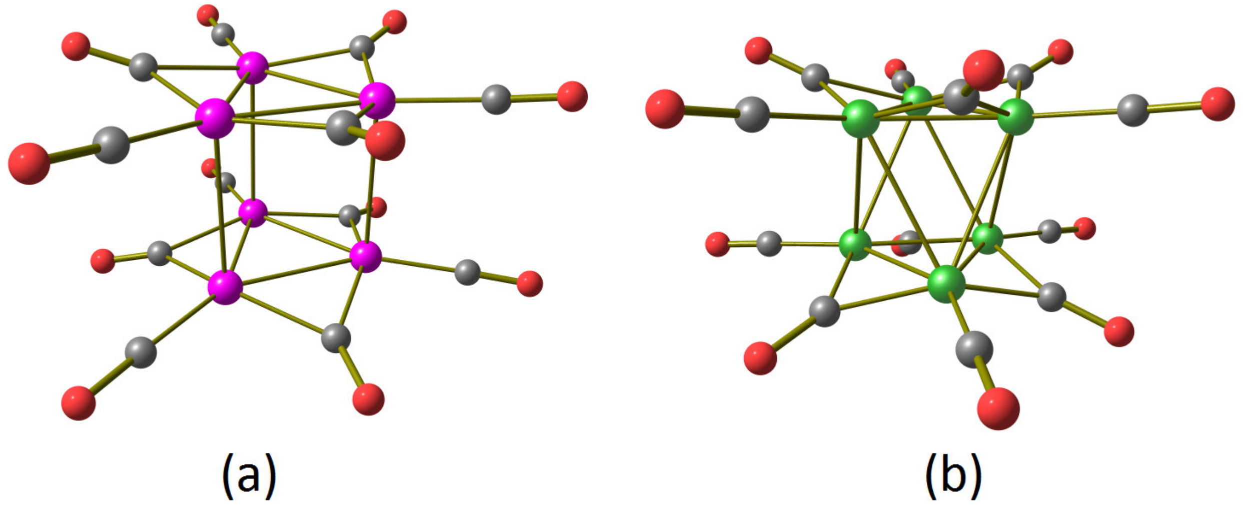

Figure S57

Relative energy variations of clusters 1 and 2 on changing the dihedral angle defining the relative position of the two {M_3} triangles. Solid line: single point calculations. Dashed line: geometry optimizations with constrained inter-triangular dihedral angles.

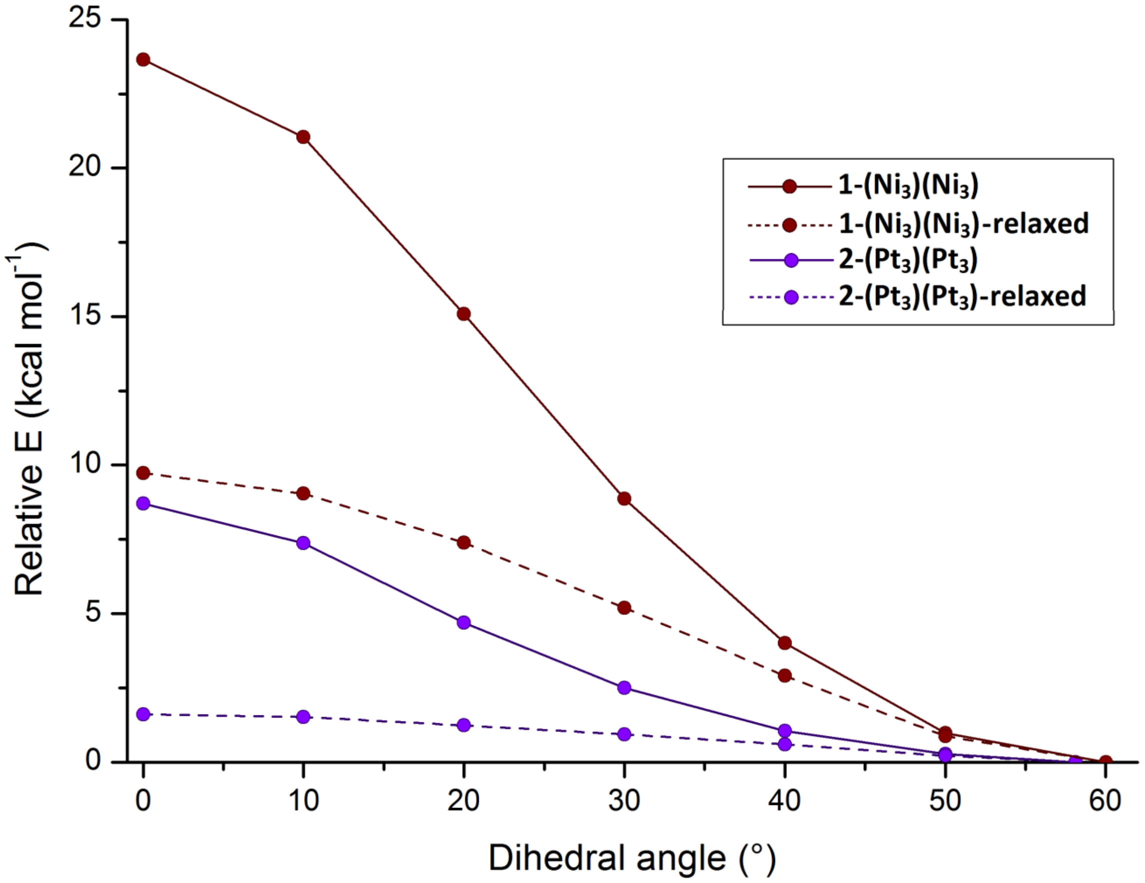

Figure S58

HOMOs of clusters **1-10**, DFT-optimized octahedral structures. Surface isovalue = 0.025 a.u.

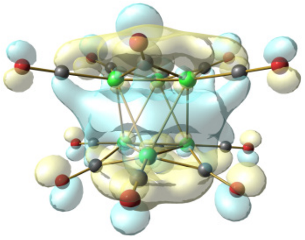

1-$(Ni_3)(Ni_3)$

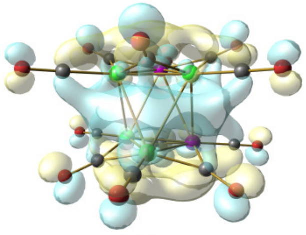

6-$(PtNi_2)(PtNi_2)$

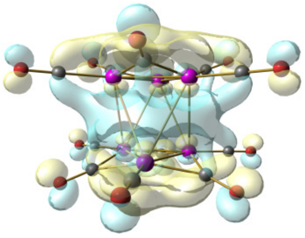

2-$(Pt_3)(Pt_3)$

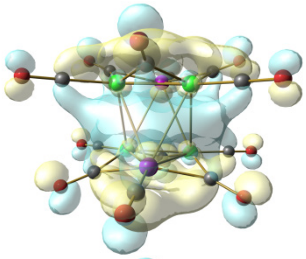

6’-$(PtNi_2)(PtNi_2)$

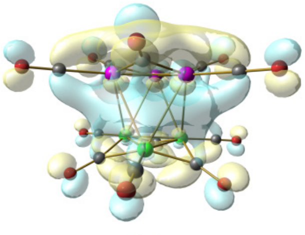

3-$(Pt_3)(Ni_3)$

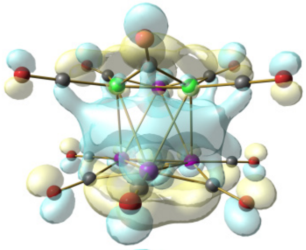

7-$(Pt_3)(PtNi_2)$

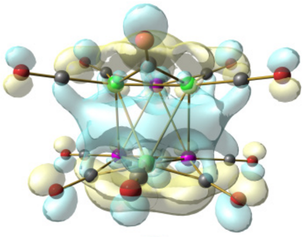

4-$(Pt_2Ni)(PtNi_2)$

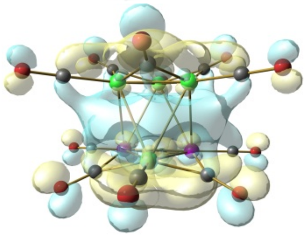

8-$(Ni_3)(Pt_2Ni)$

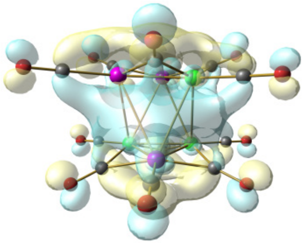

4’-$(Pt_2Ni)(PtNi_2)$

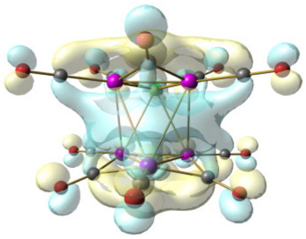

9-$(Pt_3)(Pt_2Ni)$

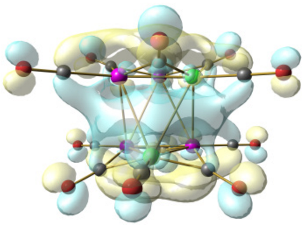

5-$(Pt_2Ni)(Pt_2Ni)$

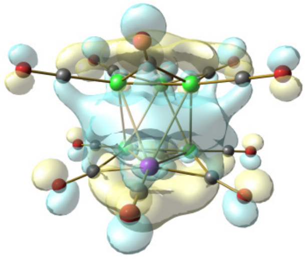

10-$(Ni_3)(PtNi_2)$

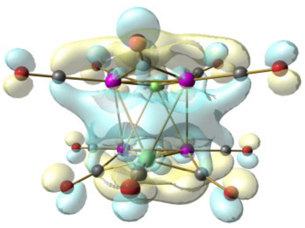

5’-$(Pt_2Ni)(Pt_2Ni)$

Figure S59

HOMOs of clusters **1-10**, single point calculations on trigonal prismatic conformations. Surface isovalue = 0.025 a.u.

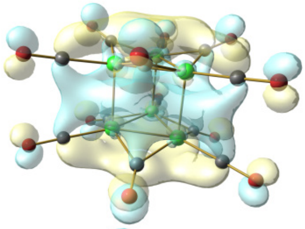

1-(Ni_3)(Ni_3)

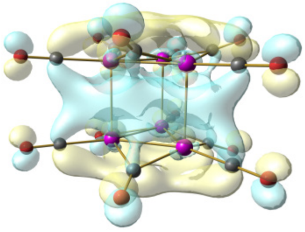

2-(Pt_3)(Pt_3)

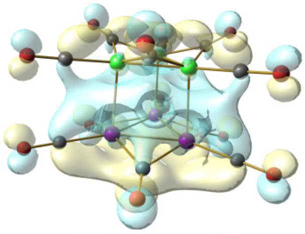

3-(Pt_3)(Ni_3)

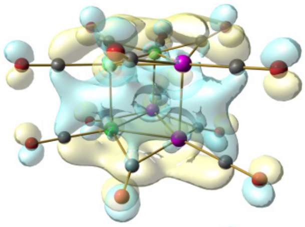

4-(Pt_2Ni)($PtNi_2$)

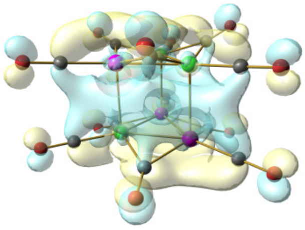

4'-(Pt_2Ni)($PtNi_2$)

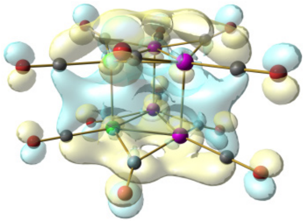

5-(Pt_2Ni)(Pt_2Ni)

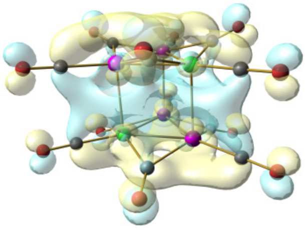

5'-(Pt_2Ni)(Pt_2Ni)

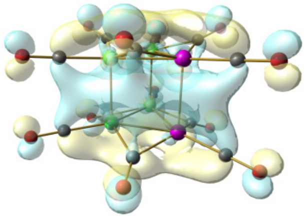

6-($PtNi_2$)($PtNi_2$)

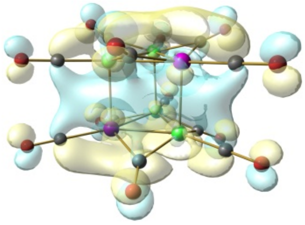

6'-($PtNi_2$)($PtNi_2$)

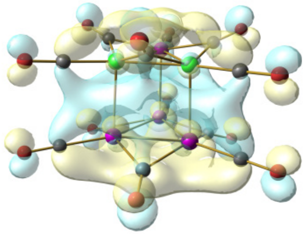

7-(Pt_3)($PtNi_2$)

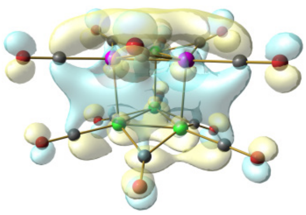

8-(Ni_3)(Pt_2Ni)

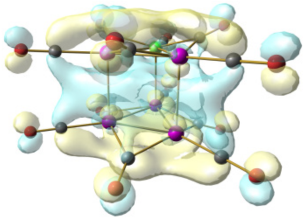

9-(Pt_3)(Pt_2Ni)

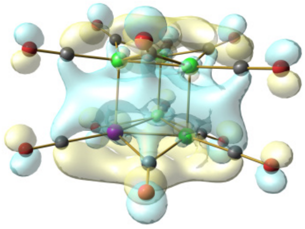

10-(Ni_3)($PtNi_2$)

Figure S60

HOMO-1 and HOMO-2 orbitals of $[Ni_6(CO)_{12}]^{2-}$ and $[Pt_6(CO)_{12}]^{2-}$, DFT-optimized octahedral geometry. Surface isovalue = 0.025 a.u.

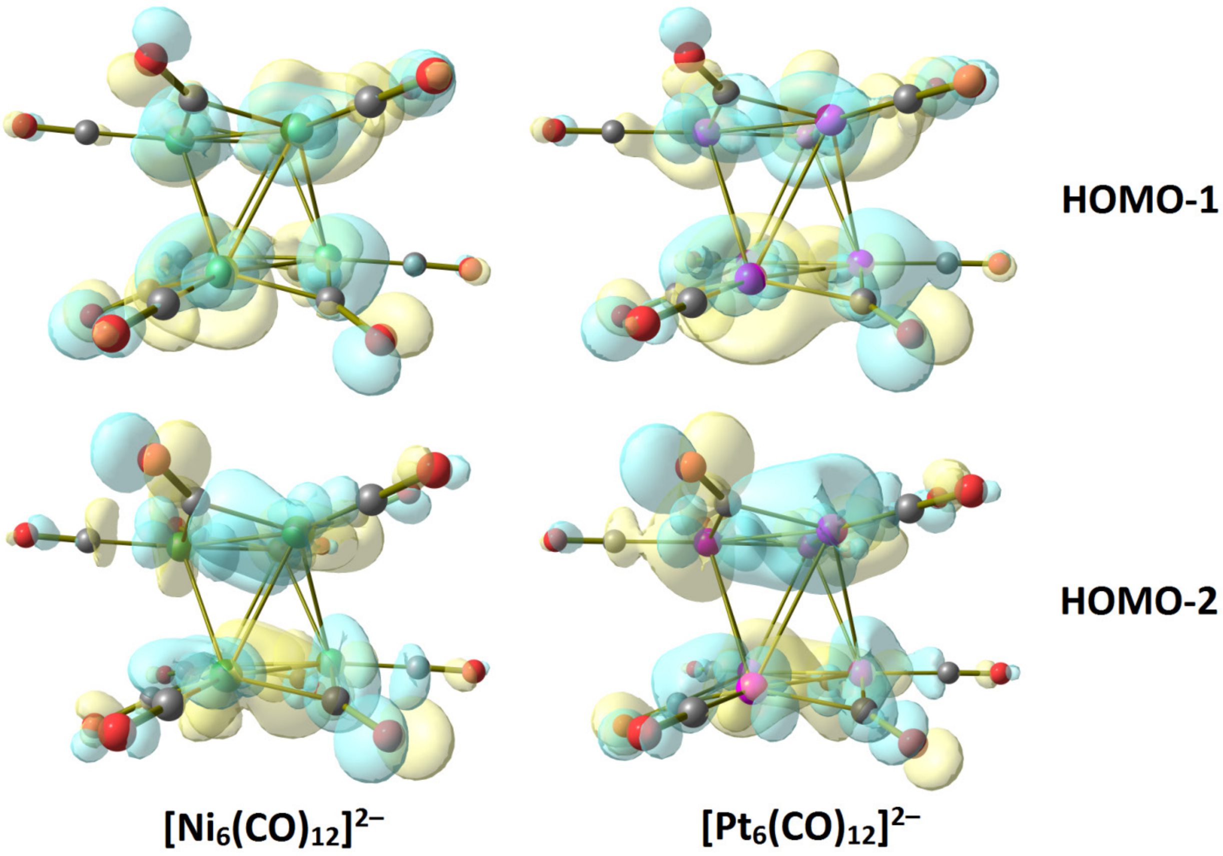

References

(1) Sheldrick, G. M. *SADABS-2008/1 - Bruker AXS Area Detector Scaling and Absorption Correction*; Bruker AXS: Madison, WI, **2008**.

(2) Sheldrick, G. M. Crystal Structure Refinement with SHELXL. *Acta Crystallogr., Sect. C: Struct. Chem.* **2015**, *C71*, 3-8.

(3) Staroverov, V. N.; Scuseria, E.; Tao, J.; Perdew, J. P. Comparative assessment of a new nonempirical density functional: Molecules and hydrogen-bonded complexes. *J. Chem. Phys.* **2003**, *119*, 12129-12137.

(4) (a) Weigend, F.; Ahlrichs, R. Balanced basis sets of split valence, triple zeta valence and quadruple zeta valence quality for H to Rn: Design and assessment of accuracy. *Phys. Chem. Chem. Phys.* **2005**, *7*, 3297-3305. (b) Andrae, D.; Häußermann, U.; Dolg, M.; Stoll, H.; Preuß, H. Energy-adjusted *ab initio* pseudopotentials for the second and third row transition elements. *Theor. Chim. Acta* **1990**, *77*, 123-141.

(5) (a) Neese, F. The ORCA Program System. *WIREs Comput. Mol. Sci.* **2012**, *2*, 73-78. (b) Neese, F. Software update: the ORCA program system, version 4.0. *WIREs Comput. Mol. Sci.* **2018**, *8*, e1327.
